# Impact of charges on the hybridization kinetics and thermal stability of PNA duplexes†

**DOI:** 10.1039/d4ob00887a

**Published:** 2024-06-19

**Authors:** Miguel López-Tena, Nicolas Winssinger

**Affiliations:** a Department of Organic Chemistry, NCCR Chemical Biology, Faculty of Science, University of Geneva 1211 Geneva Switzerland nicolas.winssinger@unige.ch

## Abstract

Peptide nucleic acid (PNA) is a prominent artificial nucleic acid mimetic and modifications at the γ-position of the peptidic backbone are known to further enhance the desirable properties of PNA in terms of duplex stability. Here, we leveraged a propargyl ether modification at this position for late stage functionalization of PNA to obtain positively charged (cationic amino and guanidinium groups), negatively charged (anionic carboxylate and alkyl phosphonate groups) and neutral (PEG) PNAs to assess the impact of these charges on DNA : PNA and PNA : PNA duplex formation. Thermal stability analysis findings concurred with prior studies showing PNA : DNA duplexes are moderately more stable with cationic PNAs than anionic PNAs at physiological salt concentrations. We show that this effect is derived predominantly from differences in the association kinetics. For PNA : PNA duplexes, anionic PNAs were found to form the most stable duplexes, more stable than neutral PNA : PNA duplexes.

## Abstract 2

Clicking-on side chains to assess the impact of charges on hybridization properties of PNA.

## Graphical abstract

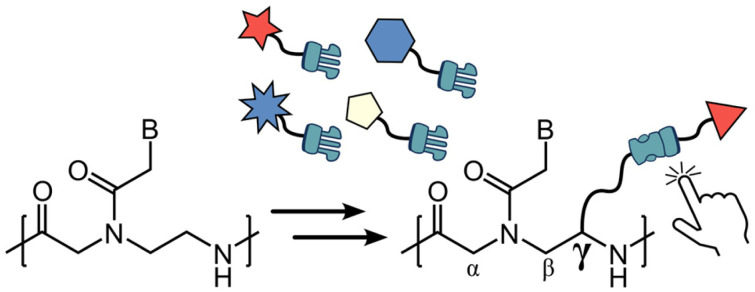

## Introduction

**Fig. 1:**
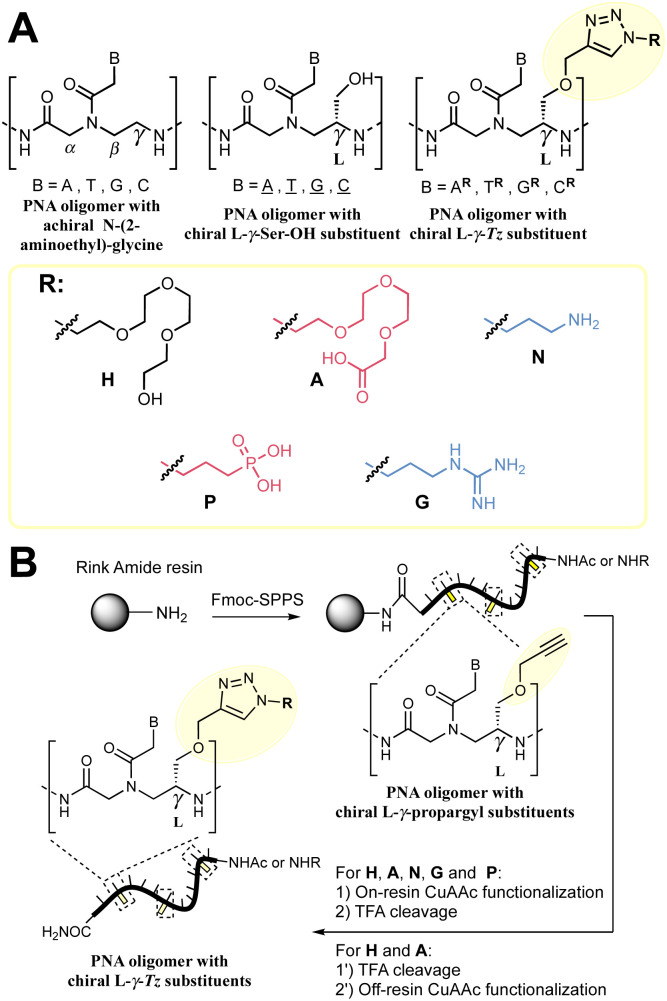
(A) Summary of the nomenclature of PNA structures used in this work together with the γ-*Tz* modifications. (B) Workflow followed for the synthesis γ-*Tz* PNAs on or off-resin.

Peptide nucleic acid (PNA) was first reported three decades ago^1,2^ and the field continues to draw attention from the community by virtue of its exceptional properties (for recent examples, see ref. 3–16). It is an artificial mimetic of DNA or RNA wherein the phosphoribosyl backbone has been replaced by a peptidic backbone. The high stability of PNA : DNA duplexes was attributed to the lack of repulsive interactions between the negatively charged DNA and the neutral PNA, compared to a DNA : DNA duplex.^1^ The higher stability of the PNA : DNA or PNA : RNA duplexes relative to DNA or RNA homoduplexes has inspired many biomedical applications including in diagnostics and therapeutics^17,18^ in addition to their use as encoding or biosupramolecular tags for assembly and hybridization circuitry.^19–21^ Extensive SAR studies have been performed to further optimise PNA properties.^22^ Notably, alternative nucleobases^23–30^ have been advanced for improved affinity and cellular uptake; conformationally restricted backbones^31–35^ have been shown to enhance duplex stability significantly; and substitution of the achiral 2-aminoethyl glycine has been used to tune and improve the properties of PNAs. In particular, substitution at the γ-position of the 2-aminoethyl glycine backbone has been found to enhance hybridization to DNA by chirality-induced preorganization (Fig. 1A).^36,37^ Examples with a broad array of substituents have been reported.^38–51^

We recently reported the synthesis of a γ-modified PNA with a propargyl ether side chain to facilitate late-stage functionalization of PNA oligomers by CuAAC (γ-*Tz*, Fig. 1B);^52^ however, the impact of the ensuing triazole functionality on the hybridization properties was not studied in detail. Herein, we report this analysis and revisit the impact of charges on duplex stability with a comparison of PNAs bearing cationic, anionic, and neutral modifications using thermal stability and kinetics of hybridization for both PNA : DNA and PNA : PNA duplexes.^52^

## Results and discussion

**Fig. 2:**
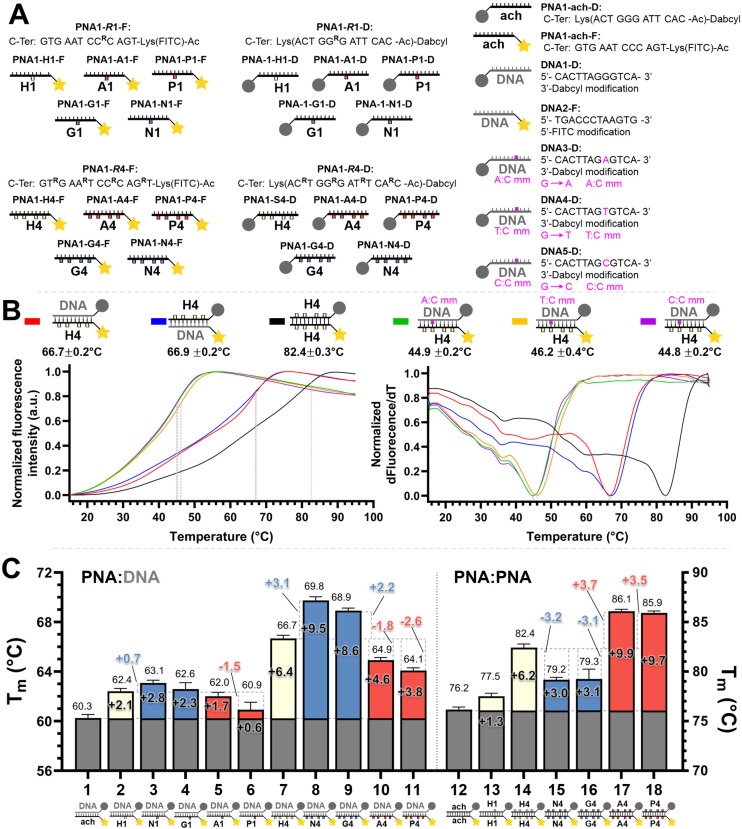
(A) Summary of the oligonucleotide sequences used for FRET melting temperature measurements. (B) Example of normalized melting profiles for the PNA1-H4-F and PNA1-H4-D series, including the normalized first derivative of the melting profiles *versus* temperature. Dotted lines indicate the inflexion points of the curves taken as the melting temperature (*T*_m_) for each duplex. The colour legend is indicated next to the duplex. Conditions: 0.5 μM each oligonucleotide strand in 1× PBS pH 7.4 buffer. (C) PNA : DNA and PNA : PNA duplex melting temperatures. The colours in the bars denote the charge with the following legend: white: neutral, blue: positive, red: negative, as in Fig. 1. Conditions: 0.5 μM each oligonucleotide strand in 1× PBS pH 7.4 buffer. Further details of the explicit structures and protocols are available in the ESI.† Data presented as the average of the individual *T*_m_ values obtained from 2 independent samples measured in triplicate (*n* = 6) and presented as the mean ± 95% CI (*z* = 1.96).

We set out to compare the affinity of PNAs bearing one to four γ-modifications covering different physicochemical space (Fig. 1A), a PEG chain as a hydrophilic neutral modification (H), the same PEG chain terminating with a carboxylic acid (A) rather than a hydroxyl group, a phosphonic acid (P), an amino group (N) and a guanidino group (G). The different PNAs were synthesised by traditional Fmoc-based SPPS and functionalized by CuAAc prior to cleavage from the resin (Fig. 1B, the sequence of each PNA is shown in the figure it is reported in, see Fig S1 for the explicit structure of each PNA†). Alternatively, the functionalisation could also be performed after cleavage from the resin (performed for H and A, Fig. 1B). Both approaches afforded the desired product as a single major product. Traditionally, the melting temperatures of hybridization duplexes are measured *via* the change in absorption related to π → π* transitions of the nucleobases (260 nm) which shifts upon duplex formation due to the hydrogen-bond network. However, in our experience, this technique does yield inflection points that are as sharp with PNA as with DNA. We opted to include a fluorophore (FITC) or quencher (Dabcyl) on the PNA (introduced during the SPPS, see the ESI for full synthetic details†) to make use of FRET to measure the melting temperature (Fig. 2). This allows multiple measurements to be performed in parallel using a qPCR instrument and facilitates *T*_m_ measurements at lower concentrations. As shown in Fig. 2B, this approach yielded sharp inflection points for *T*_m_ assignment. Comparison between *T*_m_ measurements with the fluorophore on the PNA and quencher on the DNA or *vice versa* afforded nearly identical curves suggesting that nonspecific interactions between the fluorophore or quencher and the strand it is attached to are negligible (Fig. 2B). Comparison of *T*_m_ measurements in PBS pH 7.4 (NaCl 137 mM, KCl 2.7 mM, NaHPO_4_ 10 mM, KH_2_PO_4_ 1.8 mM) across different modifications (H, N, G, A, P) showed that all modifications afforded higher duplex stability relative to an unmodified (achiral) PNA, corroborating the generality of the benefits of γ-modifications^36,37^ (Fig. 2C). The effect was additive and PNAs bearing four γ-modifications afforded higher duplex stability than PNAs bearing a single γ-modification. The PNAs with cationic side chains (N, G) had the largest benefit and the PNAs with negative side chains (A, P) had the smallest gain. These results corroborate the prior study reported by Heemstra and coworkers^46^ comparing the impact of three types of modification at the γ-position using PNA synthetically derived from alanine, lysine and aspartic acid. The data reported herein shows that the nature of the cation or anion does not have a profound impact (N *vs.* G or A *vs.* P) on this trend. Importantly, a thorough analysis of the impact of a mismatch showed that the gain in duplex stability does not come at the detriment of selectivity; the penalty for the single base pair mismatch (Δ*T*_m_) ranged from −24.0 to −24.2 °C in achiral PNA, −24.0 to −26 °C for H1 (1 sidechain modification) and −24.8 to −25.4 °C for G1 (Fig. S2†). Collectively, the data shows that while a negative charge on the PNA is detrimental to the DNA : PNA duplex stability, as could be expected on the basis of electrostatic repulsion,^1,46^ the effect is inferior to the benefit conferred by the preorganisation afforded by the γ-modification. However, the impact of charges had not been investigated for PNA : PNA duplexes. As expected based on the chirality-induced preorganization of γ-modified PNAs, higher duplex stability was observed with increasing numbers of modifications (Fig. 2C). Surprisingly, the largest benefit was observed for the anionic PNAs (A and P), with the duplex bearing four carboxylate groups (A4) on each strand gaining 9.9 °C in duplex stability relative to the achiral PNA and 3.7 °C relative to the PEG-modified PNA (H4, Fig. 2C). While this observation does not mirror what is seen with DNA : PNA, the density of charges on PNA is significantly lower that on DNA where an anion is present on every position of the oligonucleotide *vs.* four positions out of twelve in the PNA duplexes studied here.

**Fig. 3:**
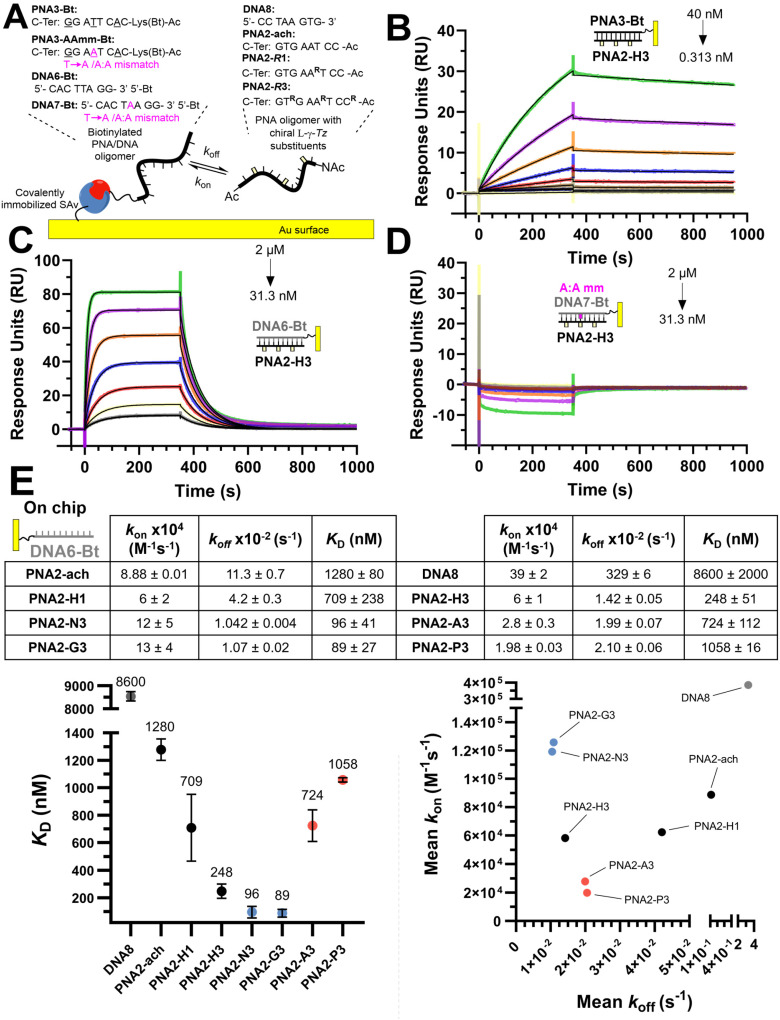
(A) Illustration of the experimental SPR set-up used for the oligonucleotide hybridization experiments. (B) SPR association and dissociation curves (coloured) with curves fitted to a 1 : 1 binding model (black lines) for PNA2-H3 and PNA3-Bt immobilized on the chip in a 2-fold dilution series from 40 nM to 0.313 nM. (C) SPR association and dissociation curves (coloured) with curves fitted to a 1 : 1 binding model (black lines) for PNA2-H3 and DNA6-Bt immobilized on the chip in a 2-fold dilution from 2 μM to 3.13 nM. (D) SPR association and dissociation curves (coloured) with curves fitted to a 1 : 1 binding model (black lines) for PNA2-H3 and DNA7-Bt (Tamm) immobilized on the chip in a 2-fold dilution from 2 μM to 3.13 nM. (E) SPR dissociation equilibrium constants (*K*_D_), association rates (*k*_a_) and dissociation rates (*k*_d_) for PNAs for DNA6-Bt immobilized on the chip. Data presented as the average of duplicate measurements (*n* = 2) and reported as the mean ± 95% CI (*z* = 1.96). Plotted error bars represent the standard deviation (SD).

We next investigated the kinetics of hybridization which is an important consideration in the designed hybridization-based circuitry^13,21,53–58^ and had not been thoroughly examined with γ-modified PNAs. As shown in Fig. 3, the affinities calculated from the kinetic data follow the same trends as observed for the *T*_m_, namely a PNA with three γ-modified residues forms a more stable duplex with DNA than the corresponding PNA with a single modification (H3 *vs.* H1); cationic PNAs form more stable duplexes with DNA than anionic PNAs (N3 or G3 *vs.* A3 or P3). A PNA with a single nucleotide mismatch had no measurable affinity under these assay conditions. Despite the overall higher duplex stability of PNA : DNA duplexes relative to DNA : DNA duplexes, the rate of hybridization (*k*_on_) is overall slower. Surprisingly, the benefit of γ-modification in duplex stability is not reflected in the rate of association (*k*_on_) as could have been anticipated based on the chirality-induced preorganization that it confers (PNA2-ach *vs.* PNA2-H1 & H3) but rather it results in a slower rate of dissociation (*k*_off_). However, when comparing the effect of charges at the γ-position on the hybridization kinetics, the charge on the PNA impacts the association kinetics more (6-fold difference between P3 *vs.* G3) than the dissociation (2-fold difference between P3 *vs.* G3). The faster *k*_on_ of cationic PNA is in-line with the findings of Windsor and co-workers^29^ but a direct comparison should be treated with caution because the charges were incorporated on the nucleobase (achiral backbone) in this prior study rather than at the γ-position of the backbone as in the present study.

**Fig. 4:**
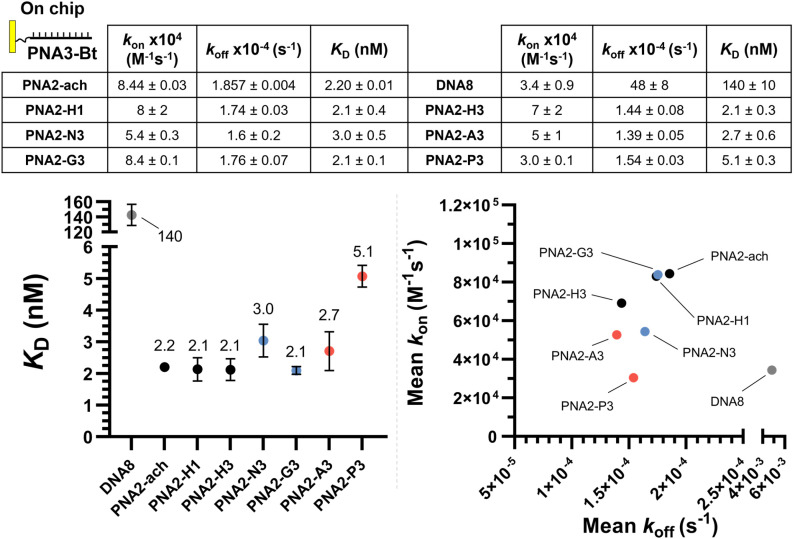
SPR dissociation equilibrium constants (*K*_D_), association rates (*k*_a_) and dissociation rates (*k*_d_) for PNAs for PNA3-Bt immobilized on the chip. Data presented as the average of duplicate measurements (*n* = 2) and reported as the mean ± 95% CI (*z* = 1.96). Plotted error bars represent the standard deviation (SD).

The same kinetic analysis for PNA : PNA duplexes (Fig. 4) showed that the rate of hybridization (*k*_on_) for the formation of a PNA : PNA duplex is comparable to that of a PNA : DNA duplex with a neutral achiral PNA (8 × 10^4^ M^−1^ s^−1^ at 25 °C). The better duplex stability of PNA : PNA *vs.* PNA : DNA arises from the slower dissociation kinetics (1.74 × 10^−4^ s^−1^*vs.* 11.3 × 10^−2^ s^−1^). However, the presence of charges on one strand has negligible impact on hybridization when the other complementary strand is neutral. This comparison further supports the assertion that the differences in the kinetics of hybridization of charged PNAs to DNA is due to electrostatic interactions between the oligomers (*i.e.* slower *k*_on_ for anionic PNA *vs.* cationic PNA). While γ-modifications improve the duplex stability of both PNA : DNA and PNA : PNA duplexes, it is important to note that the DNA : DNA duplex has the fastest association kinetics, despite the inherent repulsive interaction of the negative charges present in this duplex. This suggests that, while γ-modifications on PNA are beneficial, they do not preorganize the single strand oligomer in the duplex conformation as well as the phosphoryl ribose backbone does.

We next compared the hybridization kinetics of PNA containing pseudo-complementary C–G (G-clamp and *N*-7 methyl guanine)^28^ in combination with γ-modifications (Fig. S4†). The pseudo-complementary nucleobases are cationic, thus their inclusion on the achiral PNA backbone without γ-modifications results in a cationic PNA. Concurring with our previous report,^28^ the combination of these nucleobases significantly improves the stability of a PNA : DNA duplex (*K*_D_ 48.1 nM *vs.* 1280 nM). This improvement comes from both a faster association kinetics (4-fold) and slower dissociation kinetics (6-fold). Addition of γ-modifications resulted in the same affinity trend as before (neutral modifications lead to better duplex stability; negative charges slow down the association kinetics). It is interesting to note that in this case, the γ-modification with a neutral group (PNA2-pc-H3) led to slower association kinetics than the achiral backbone (PNA2-pc-ach).

## Conclusions

The data reported provides a detailed landscape of the impact of modifications at the γ-position of PNA oligomers on hybridization stability and kinetics. This analysis was facilitated by the use of late-stage functionalization of the γ-position (γ-*Tz* modifications) with various side chains enabling a comparison across different charge statuses of PNA oligomers in their hybridization to DNA or PNA. All tested γ-*Tz* modifications were beneficial in duplex stability for both PNA : DNA and PNA : PNA duplexes. While a negatively charged PNA forms a less stable duplex with DNA than a positively charged PNA due to electrostatic interactions, the detriment of the negative charges is outweighed by the benefit of γ-modifications. The lower affinity of negatively charged PNA towards DNA comes predominantly from slower association kinetics. The higher stability of duplexes involving PNA comes from slower dissociation kinetics relative to the DNA : DNA duplex. The fact that γ-modified PNA hybridizes to DNA slower than DNA despite the lack of electrostatic repulsion suggests that further improvements in preorganization are possible.

## Experimental section

### General methods

All reagents and solvents for the organic synthesis were purchased from commercial sources and were used without further purification. Fmoc-Rink-Amide PEG AM resin for peptide synthesis was obtained from Iris Biotech. DNA oligonucleotides were bought from Eurogentec with the desired modifications and used without any further purification. HPLC purification was performed with an Agilent Technologies 1260 infinity HPLC using a ZORBAX 300SB-C_18_ column (9.4 × 250 mm). LC-MS spectra were recorded on a DIONEX Ultimate 3000 UHPLC (conditions for elution gradient: 0 min, A : B = 100 : 0; 4 min, A : B = 10 : 90; solution A: 0.01% aqueous TFA solution; solution B, 0.01% TFA in HPLC grade acetonitrile; flow rate: 0.750 mL min^−1^) with a Thermo LCQ Fleet Mass Spectrometer System using a PINNACLE DB C_18_ column (1.9 μm, 50 × 2.1 mm) operated in positive mode. All the LC-MS spectra were measured by electrospray ionization (ESI), linear gradient 0 to 100%. MALDI-TOF mass spectra were measured using a Bruker Daltonics Autoflex spectrometer operated in positive mode. The samples were analysed using a 2,5-dihydroxybenzoic acid (DHB) matrix. NMR spectra were acquired at the University of Geneva NMR platform (https://www.unige.ch/sciences/chiorg/nmr/) using either a 500 MHz Avance III Bruker NMR spectrometer equipped with a helium-cooled cryogenic 5 mm DCH ^13^C–^1^H/D Bruker probe, a 400 MHz Avance III HD NanoBay spectrometer equipped with a N_2_ Prodigy cryogenic 5 mm CPP BB(F)–H–D probe or a 300 MHz Avance III, HD NanoBay spectrometer, equipped with a 5 mm PA BBO, BB(F)–H–D probe. All ^1^H and ^13^C ^59^ experiments were internally referenced with respect to either DMSO-d_6_ or CDCl_3_ solvent signals and acquired at 298 K. Retention times (RT) are given in minutes. Thin layer chromatography (TLC) was performed on plates of silica precoated with 0.25 mm Kieselgel 60 F254 from Merck. Flash chromatography was performed using silica gel SiliaFlash® P60 (230–400 mesh) from Silicycle. Automated solid-phase synthesis was carried out on an Intavis AG Multipep RS instrument. Concentrations of the PNA and DNA stocks were measured with a NanoDrop^RM^ 2000c at *λ* = 260 nm. High-resolution mass spectra (HRMS) were obtained on a Xevo G2 Tof spectrometer (ionization mode: ESI positive polarity; mobile phase: MeOH 100 μl min^−1^). All statistical data were calculated using GraphPad Prism and are presented as the mean value ± SD unless otherwise noted.

### Surface plasmon resonance (SPR) measurements

SPR experiments were performed on a Biacore T200 instrument (GE Healthcare) at 25 °C in PBS-P+ buffer (10× stock from Cytiva Life Sciences, 28995084). Streptavidin was chemically immobilized *via* NHS/EDC coupling on a CM5 sensor chip (Cytiva Life Sciences, 29104988). Immobilization was performed following the standard Cytiva protocol (amine coupling of ligand to Biacore sensor chips) using streptavidin at 20 μg mL^−1^, contact time of 600 s and flow rate of 10 μL min^−1^. Subsequently, PNA3-Bt/PNA3-AAmm-Bt/DNA6-Bt/DNA7-Bt were independently immobilized on the previous streptavidin coated chip. Prior to immobilization, the two channels were conditioned with 1 M NaCl in 50 mM NaOH. After stabilization, the biotinylated compound was flowed over one of the flow cells of the sensor chip at a concentration of 50 nM solution in PBS-P+ at a flow rate of 10 μL min^−1^ with a response unit (RU) target of 100. The system (not including the flow cells) was washed with 50% isopropanol in 1 M NaCl and 50 mM NaOH after each ligand injection. Kinetic measurements consisted of injections (association 350 seconds, dissociation 650 seconds, flow rate: 30 μL min^−1^) of decreasing concentrations of PNAs (2-fold cascade dilutions from the starting concentration). The chip was regenerated between cycles by one injection of regeneration solution (50 mM NaOH) for 10 seconds at a flow rate of 20 μL min^−1^, followed by a 10 second stabilization period. Binding was measured as resonance units over time after blank subtraction, and the data interpreted using the Biacore T200 software, version 3.2. All measurements were repeated in duplicate for PNAs *versus* PNA3-Bt/DNA6-Bt and once for PNAs *vs.* PNA3-AAmm-Bt/DNA7-Bt. The *K*_D_ values were calculated based on steady-state affinity (1 : 1 binding).

### FRET melting temperature measurements

FRET melting temperature measurements were performed in a parallel fashion in Hard-Shell® 96-Well PCR Plates, low profile, thin wall, skirted, black/white (BioRad, #HSP9665) using a CFX Connect Real-Time PCR Detection System. 50 μL 0.5 μM of each oligomer in 1× PBS (137 mM NaCl, 2.7 mM KCl, 10 mM Na_2_HPO_4_, 1.8 mM KH_2_PO_4_) was placed in each well. Each individual sample was annealed to 95 °C for 3 min before gradual cooling to 15 °C over the course of 2 h, then gradual heating up to 95 °C at a rate of 0.5 °C per min while monitoring the FITC fluorescence emission signal (excitation: 450–490 nm, emission: 515–530 nm). Then, samples were kept at 95 °C for 3 min before again gradual cooling to 15 °C over the course of 2 h, followed by gradual heating up to 95 °C at a rate of 0.5 °C per min while monitoring the FITC fluorescence emission signal. This iteration was repeated once more with a total of three measurements per individual sample, each sample was done in duplicate. For each individual curve, the first derivative of the fluorescence emission of FITC *versus* temperature was calculated, and the temperature with the minimum value of the first derivative was assigned as the melting temperature (*T*_m_), being the inflexion point of the melting curve. Data were presented as the average of the 6 individual *T*_m_ values obtained from 2 independent samples measured in triplicate and presented as the mean ± 95% CI (*z* = 1.96).

### Click on/off-resin after SPPS

#### Click on-resin general protocol for Tz-H, A, N, G and P

The Cu-click mixture was prepared as follows: 50 μL THPTA 200 mM (10 μmol) in H_2_O was mixed with 75 μL CuSO_4_ 134 mM (10 μmol) in H_2_O, producing a deep blue solution. Then, 20 μL NaAsc 1 M (20 μmol) in H_2_O was added, to give a pale yellow solution. Next, 100 μL of 100 mM in DMSO of Az-H, A, N, G or P (10 μmol) was added to give the Cu-click mixture as a yellow solution. After Fmoc-based SPPS, the above fresh Cu-click mixture was added to 5.0 mg of Fmoc-Rink-Amide PEG AM resin (0.33 mmol g^−1^, 1.65 μmol) containing the crude PNAs. The reaction was left overnight at room temperature. After completion, the resin was washed with MeOH, H_2_O, DMF and DCM (10 × volume of resin) before cleavage with TFA. After TFA cleavage for 2 h at room temperature (for Az-P, to drive the debenzylation to completion, the TFA cleavage was done at 60 °C for 2 h), cold ether was added followed by centrifugation, the ethereal layer was discarded, and the resulting pellet was air dried before proceeding to HPLC purification of the crude oligomers.

#### Click off-resin general protocol for Tz-H and A

The Cu-click mixture was prepared as follows: 22 μL THPTA 90 mM (2 μmol) in H_2_O was mixed with 15 μL CuSO_4_ 134 mM (2 μmol) in H_2_O, giving a deep blue solution. Then, 4 μL NaAsc 1 M (4 μmol) in H_2_O was added, to give a pale yellow solution, with a total volume of 41 μL.

After Fmoc-SPPS, the Fmoc-Rink-Amide PEG AM resin (0.33 mmol g^−1^, 1.65 μmol) was cleaved with TFA for 2 h at room temperature. The filtrates were added to cold ether and following centrifugation, the ethereal was layer discarded. The resulting pellet was air dried. Then, it was dissolved in DMSO/H_2_O 1 : 1 and the crude propargyl PNA concentration was assessed by absorption. Typically, 100 μL 2 mM crude PNA in DMSO/H_2_O 1 : 1 (0.2 μmol), was diluted with 57 μL DMSO. Then, 41 μL of the above Cu-click mixture [2 μmol THPTA, 2 μmol CuSO_4_, 4 μmol NaAsc] was added and vortexed before adding 2 μL 100 mM in DMSO of either Az-H or Az-A (2 μmol) making a total volume of 200 μL. The mixture was left overnight at room temperature before HPLC purification by direct injection of the crude oligomers.

### Synthesis protocols for compounds Az-H/A/N/G/P

#### 2-[2-[2-(2-Azidoethoxy)ethoxy]ethoxy]ethanol (Az-H)

Azido alcohol (Az-H) was synthesized according to the previously reported protocols.^60^

^1^H NMR (400 MHz, CDCl_3_): *δ* 3.87 (s, 1H), 3.70–3.66 (m, 2H), 3.64–3.60 (m, 12H), 3.60–3.53 (m, 2H), 3.35 (t, *J* = 5.1, 5.1 Hz, 2H).

#### 2-[2-[2-(2-Azidoethoxy)ethoxy]ethoxy]acetic acid (Az-A)

Azido acetic acid (Az-A) was synthesized according to previously reported protocols.^60^

^1^H NMR (400 MHz, CDCl_3_): *δ* 9.69 (br. s, 1H), 4.15 (s, 2H), 3.73–3.61 (m, 10H), 3.35 (t, *J* = 5.2, 5.2 Hz, 2H).

#### *tert*-Butyl (3-azidopropyl)carbamate (Az-N)

To a stirred solution of 3-azidopropan-1-amine (400 mg, 4 mmol, 1 eq.) in CH_2_Cl_2_ (5 mL) at 0 °C, triethylamine (556 μL, 4 mmol, 1 eq.) and 4-dimethylaminopyridine (24 mg, 0.2 mmol, 0.05 eq.) were added. Then, di-*tert*-butyl dicarbonate (872 mg, 4 mmol, 1 eq.) was added in one portion and the reaction mixture was left to warm to room temperature overnight. The reaction mixture was partitioned between Et_2_O (60 mL) and NH_4_Cl (sat.) (20 mL). The organic layer was separated, washed again with NH_4_Cl (sat.) (2 × 20 mL), and brine, dried over anhydrous Na_2_SO_4_, filtered, and concentrated *in vacuo*. The resulting crude residue was purified by column chromatography (10–30% EtOAc/pentane) to give Az-N. Yield: 611 mg (76%). Isolated as a pale yellow oil. Spectroscopic data in accordance with previous reports.^60^

*R*_f_: 0.70 in 1/3 EtOAc/pentane. UV inactive and stains yellow with KMnO_4_ stain. HR-MS/TOF-MS-ES^+^: *m*/*z* expected for [M + Na]^+^: 223.1171, *m*/*z* found: 223.1181. ^1^H NMR (300 MHz, CDCl_3_): *δ* 4.66 (br. s, 1H), 3.35 (t, *J* = 6.7 Hz, 2H), 3.26–3.15 (m, 2H), 1.76 (p, *J* = 6.7 Hz, 2H), 1.44 (s, 9H). ^13^C NMR (75 MHz, CDCl_3_): *δ* 156.1, 79.6, 49.3, 38.2, 29.4, 28.5.

#### *N* ^2^,*N*^3^-Bis(*tert*-butoxycarbonyl)-*N*^1^-(3-azidopropyl)guanidine (Az-G)

To a stirred solution of *N,N*′-di-Boc-*S*-methylisothiourea (300 mg, 1.03 mmol, 1 eq.) in THF (3.2 mL) at room temperature, a solution of 3-azidopropan-1-amine (258 mg, 2.58 mmol, 2.5 eq.) and triethylamine (430 μL, 3.09 mmol, 3 eq.) in THF (2 mL) was added. The reaction mixture was left to stir at 40 °C overnight. Then, it was concentrated *in vacuo* and the resulting crude residue was purified by column chromatography (10% EtOAc/pentane) to give Az-G. Yield: 272 mg (89%). Isolated as a white solid. *R*_f_: 0.30 in 10% EtOAc/pentane. UV inactive and stains yellow with KMnO_4_ stain. HR-MS/TOF-MS-ES^+^: *m*/*z* expected for [M + Na]^+^: 365.1913, *m*/*z* found: 365.1920. ^1^H NMR (300 MHz, CDCl_3_): *δ* 11.48 (br. s, 1H), 8.42 (br. s, 1H), 3.51 (m, 2H), 3.38 (t, *J* = 6.8 Hz, 2H), 1.86 (p, *J* = 6.8 Hz, 2H), 1.49 (m, 18H). ^13^C NMR (75 MHz, CDCl_3_): *δ* 163.7, 156.4, 153.4, 83.4, 79.5, 49.3, 38.3, 28.6, 28.4, 28.2.

#### Dibenzyl (3-azidopropyl)phosphonate (Az-P)

To a stirred solution of diethyl (3-azidopropyl)phosphonate (300 mg, 1.4 mmol, 1 eq.) in CH_2_Cl_2_ (1 mL) at room temperature, TMS-Br (648 μL, 4.8 mmol, 3.5 eq.) was added and stirred under a N_2_ atmosphere for 4 h. Then, the excess of TMS-Br was removed by CH_2_Cl_2_ co-evaporation (2 × 5 mL). The residual yellow oil was redissolved in CH_2_Cl_2_ (1.5 mL) with 2 drops of DMF. The solution was cooled to 0 °C and (COCl)_2_ (908 μL, 10.1 mmol, 7.4 eq.) was added dropwise under an inert atmosphere (strong bubbling). The mixture was left to warm to room temperature for 2 h before removing the excess (COCl)_2_ by CH_2_Cl_2_ co-evaporation (2 × 5 mL). The residual orange oil was redissolved in CH_2_Cl_2_ (1 mL) and cooled to 0°, then a solution of benzyl alcohol (560 μL, 5.4 mmol, 4 eq.), 4-dimethylaminopyridine (17 mg, 0.1 mmol, 0.1 eq.) and triethylamine (1.14 mL, 8.2 mmol, 6 eq.) in CH_2_Cl_2_ (1.5 mL) was added dropwise under an inert atmosphere (strongly exothermic). The reaction mixture was left to warm to room temperature for 30 min, and then partitioned between Et_2_O (60 mL) and NH_4_Cl (sat.) (20 mL). The organic layer was separated, washed again with NH_4_Cl (sat.) (2 × 20 mL), water (1 × 20 mL), 1 M NaOH (3 × 20 mL), and brine, dried over anhydrous Na_2_SO_4_, filtered, and concentrated *in vacuo*. The resulting crude residue was purified by column chromatography (10–30% acetone/pentane) to give Az-P. Yield: 121 mg (24%). Isolated as a pale yellow oil. *R*_f_: 0.60 in 30% acetone/pentane. UV active and stains yellow with KMnO_4_ stain. HR-MS/TOF-MS-ES^+^: *m*/*z* expected for [M + Na]^+^: 368.1140, *m*/*z* found: 368.1147. ^1^H NMR (400 MHz, CDCl_3_): *δ* 7.42–7.29 (m, 10H), 5.10–4.92 (m, 4H), 3.43–3.19 (m, 2H), 2.24–1.67 (m, 4H). ^13^C NMR (101 MHz, CDCl_3_): *δ* 136.4, 136.4, 128.8, 128.7, 128.1, 67.5, 67.4, 51.6, 51.4, 33.7, 33.5, 26.0, 25.9, 25.7, 24.3, 24.2, 22.7, 22.5, 22.4. ^31^P NMR (121 MHz, CDCl_3_): *δ* 34.8, 32.0 (major), 31.7.

## Conflicts of interest

There are no conflicts to declare.

## Supplementary Material

OB-022-D4OB00887A-s001

## Data Availability

The authors confirm that the data supporting the findings of this study are available within the article and in the ESI.† The raw data have been deposited on Zenodo (https://doi.org/10.5281/zenodo.11257281).
