# Supplementary material for: Impact of charges on the hybridization kinetics and thermal stability of PNA duplexes
Source: Org Biomol Chem. 2024 Jun 19;22(28):5759–67. doi: 10.1039/d4ob00887a (PMC11253249; doi:10.1039/d4ob00887a)
Supplement: OB-022-D4OB00887A-s001 [file OB-022-D4OB00887A-s001.pdf]

Electronic Supplementary Material (ESI) for Organic & Biomolecular Chemistry.
This journal is © The Royal Society of Chemistry 2024

SUPPORTING INFORMATION

Impact of charges on the hybridization kinetics and thermal duplex stability of PNA

Miguel López-Tena[a] and Nicolas Winssinger*[a]

nicolas.winssinger@unige.ch

Table of Contents

Figures S1 to S4 2

Abbreviations 7

Synthesis of PNA oligomers 8

Protocols for the synthesis of compounds Az-A/H/N/G/P 8

Characterisation of PNA oligomers 10

Copies of the SPR response curves and the 1:1 binding model fitting curves 46

Copies of the ^{1}H and ^{13}C NMR spectra for compounds Az-A/H/N/G/P 46

References 60

Figures S1 to S4

PNA1-ach-F: C-Ter: GTG AAT CCC AGT-Lys(FITC)-Ac

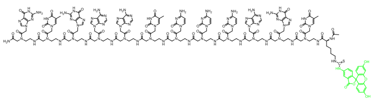

PNA1-H1-F: C-Ter: GTG AAT CC^{H}C AGT-Lys(FITC)-Ac

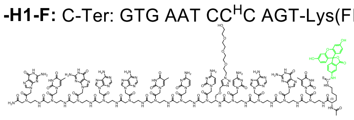

PNA1-H4-F: C-Ter: GT^{H}G AA^{H}T CC^{H}C AG^{H}T-Lys(FITC)-Ac

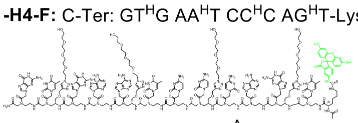

PNA1-A1-F: C-Ter: GTG AAT CC^{A}C AGT-Lys(FITC)-Ac

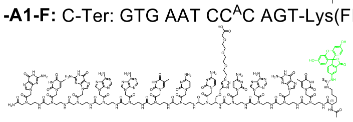

PNA1-A4-F: C-Ter: GT^{A}G AA^{A}T CC^{A}C AG^{A}T-Lys(FITC)-Ac

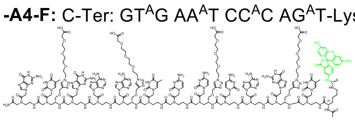

PNA1-N1-F: C-Ter: GTG AAT CC^{N}C AGT-Lys(FITC)-Ac

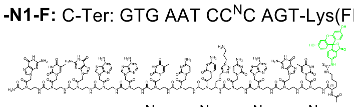

PNA1-N4-F: C-Ter: GT^{N}G AA^{N}T CC^{N}C AG^{N}T-Lys(FITC)-Ac

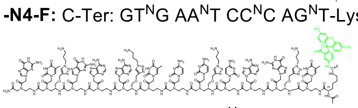

PNA1-H1-D: C-Ter: Lys(ACT GG^{H}G ATT CAC -Ac)-Dabcyl

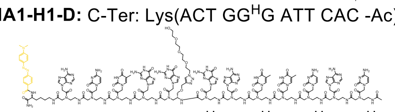

PNA1-H4-D: C-Ter: Lys(AC^{H}T GG^{H}G AT^{H}T CA^{H}C -Ac)-Dabcyl

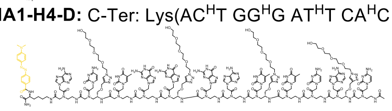

PNA1-A1-D: C-Ter: Lys(ACT GG^{A}G ATT CAC -Ac)-Dabcyl

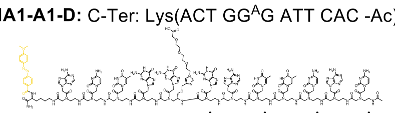

PNA1-A4-D: C-Ter: Lys(AC^{A}T GG^{A}G AT^{A}T CA^{A}C -Ac)-Dabcyl

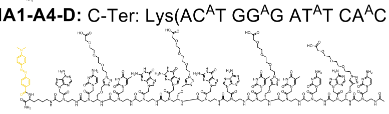

PNA1-ach-D: C-Ter: Lys(ACT GGG ATT CAC -Ac)-Dabcyl

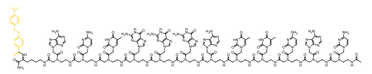

PNA1-G1-F: C-Ter: GTG AAT CC^{G}C AGT-Lys(FITC)-Ac

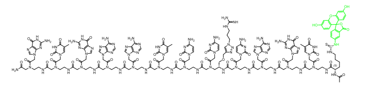

PNA1-G4-F: C-Ter: GT^{G}G AA^{G}T CC^{G}C AG^{G}T-Lys(FITC)-Ac

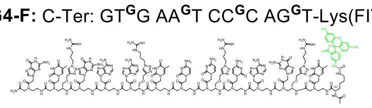

PNA-P1-F: C-Ter: GTG AAT CC^{P}C AGT-Lys(FITC)-Ac

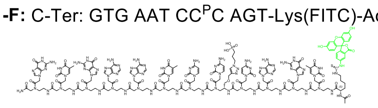

PNA1-P4-F: C-Ter: GT^{P}G AA^{P}T CC^{P}C AG^{P}T-Lys(FITC)-Ac

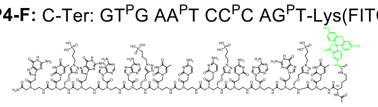

PNA1-N1-D: C-Ter: Lys(ACT GG^{N}G ATT CAC -Ac)-Dabcyl

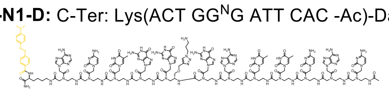

PNA1-N4-D: C-Ter: Lys(AC^{N}T GG^{N}G AT^{N}T CA^{N}C -Ac)-Dabcyl

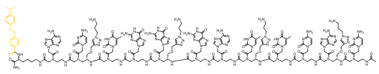

PNA1-G1-D: C-Ter: Lys(ACT GG^{G}G ATT CAC -Ac)-Dabcyl

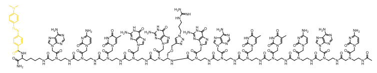

PNA1-G4-D: C-Ter: Lys(AC^{G}T GG^{G}G AT^{G}T CA^{G}C -Ac)-Dabcyl

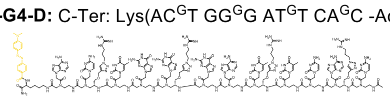

PNA1-P1-D: C-Ter: Lys(ACT GG^{P}G ATT CAC -Ac)-Dabcyl

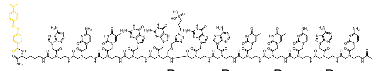

PNA1-P4-D: C-Ter: Lys(AC^{P}T GG^{P}G AT^{P}T CA^{P}C -Ac)-Dabcyl

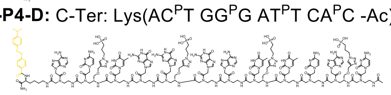

DNA1-D: 5'- CACTTAGGGTCA- 3' 3'-Dabcyl modification

DNA2-F: 5'- TGACCCTAAGTG -3' 5'-FITC modification

DNA3-D: 5'- CACTTAGAGTCA- 3' 3'-Dabcyl modification / A:C mismatch

DNA4-D: 5'- CACTTAGTGTCA- 3' 3'-Dabcyl modification / T:C mismatch

DNA5-D: 5'- CACTTAGCGTCA- 3' 3'-Dabcyl modification / C:C mismatch

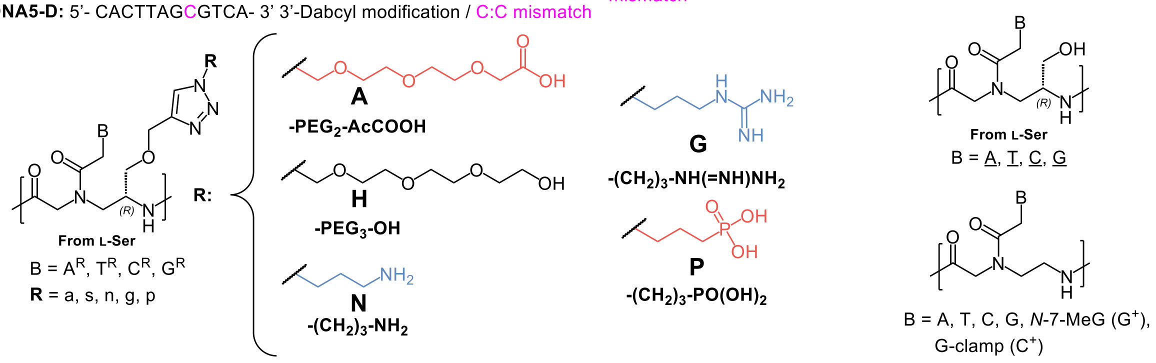

Figure S1. Detailed structures of all PNA/DNA oligomers used in this work for FRET T_m and K_D measurements.

PNA2-H1: C-Ter: GTG AA^{H}T CC -Ac

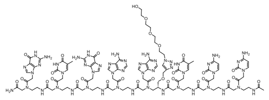

PNA2-H3: C-Ter: GT^{H}G AA^{H}T CC^{H} -Ac

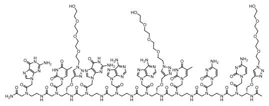

PNA2-A3: C-Ter: GT^{A}G AA^{A}T CC^{A} -Ac

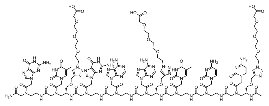

PNA2-N3: C-Ter: GT^{N}G AA^{N}T CC^{N} -Ac

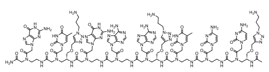

DNA6-Bt: 5'- CAC TTA GG- 3' 5'-Biotin modification

PNA2-pc-ach: C-Ter: GTG^{+} AAT C^{+}C -Ac

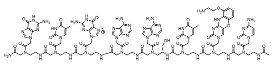

PNA2-pc-H3: C-Ter: GT^{H}G^{+} AA^{H}T C^{+}C^{H} -Ac

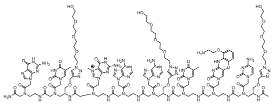

PNA3-Bt: C-Ter: GG ATT CAC-Lys(Bt)-Ac

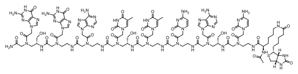

PNA3-AAmm-Bt: C-Ter: GG AAT CAC-Lys(Bt)-Ac
A:A mismatch

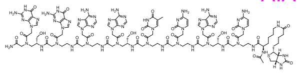

PNA2-G3: C-Ter: GT^{G}G AA^{G}T CC^{G} -Ac

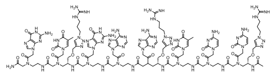

PNA2-P3: C-Ter: GT^{P}G AA^{P}T CC^{P} -Ac

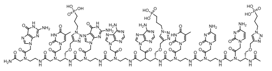

DNA7-Bt: 5'- CAC TAA GG- 3' 5'-Biotin modification /
A:A mismatch

DNA-8: 5'- CC TAA GTG- 3'

PNA2-pc-A1: C-Ter: GTG^{+} AA^{A}T C^{+}C -Ac

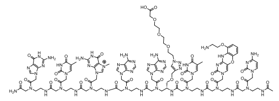

PNA2-pc-A3: C-Ter: GT^{A}G^{+} AA^{A}T C^{+}C^{A} -Ac

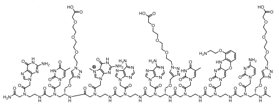

Figure S1 continued. Detailed structures of all PNA/DNA oligomers used in this work for FRET T_m and K_D measurements.

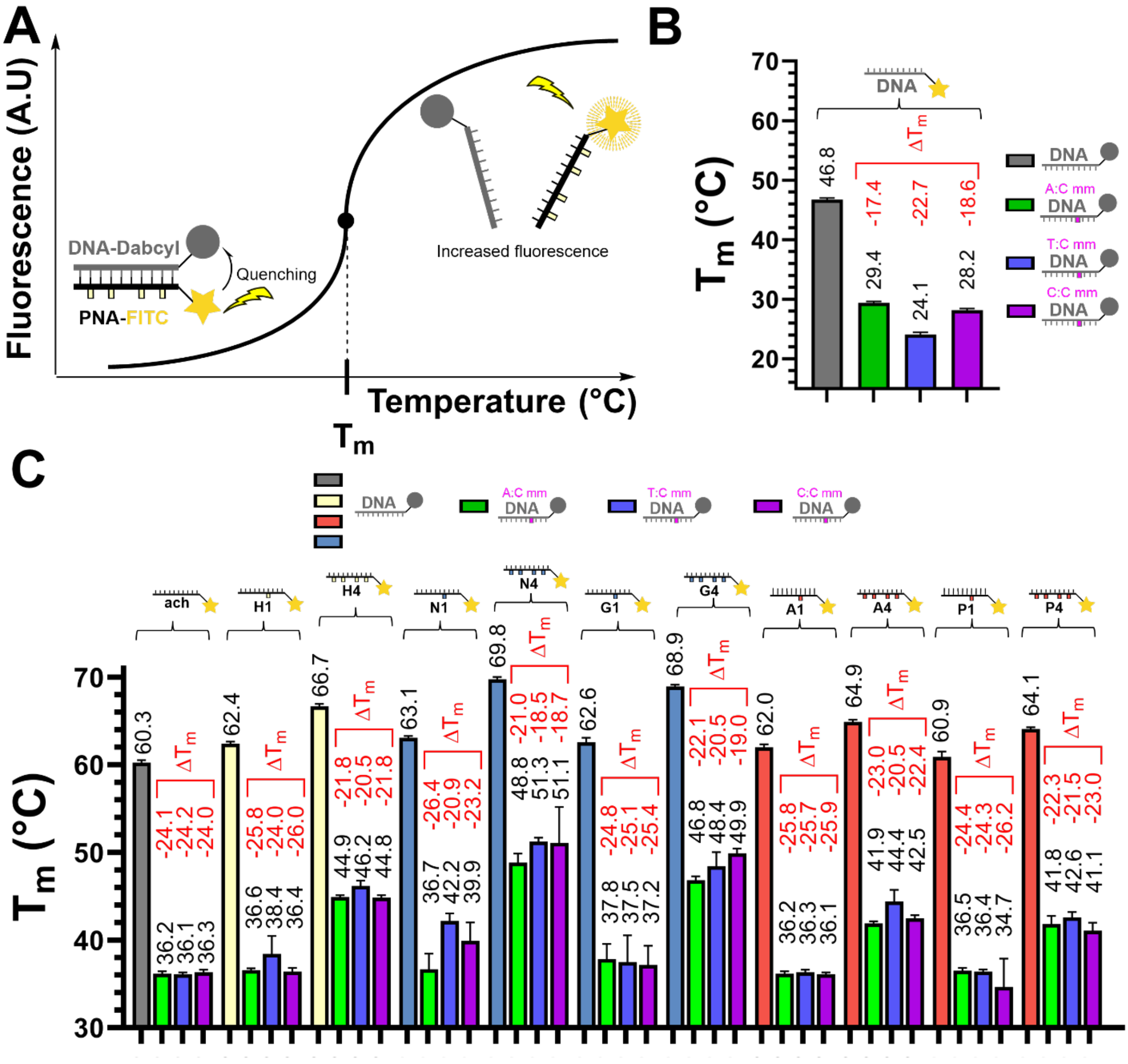

Figure S2. A) Scheme of the FRET melting temperature analysis curves for DNA labelled with Dabcyl quencher and PNA with FITC. B) Melting temperatures for DNA-DNA duplexes, perfect matched sequence versus A:Cmm, T:Cmm and C:Cmm. C) Melting temperatures for PNA-DNA duplexes, perfect matched sequence versus A:Cmm, T:Cmm and C:Cmm.

On chip

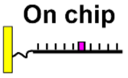

PNA3-AAmm-Bt A:A mm	k_{on} x10^4 ($M^{-1}s^{-1}$)	k_{off} x10^{-3} (s^{-1})	K_D (nM)		k_{on} x10^4 ($M^{-1}s^{-1}$)	k_{off} x10^{-3} (s^{-1})	K_D (nM)
**PNA2-ach**	3.0	2.1	71	**DNA8**	4.9	258.2	5225
**PNA2-H1**	1.4	2.0	147	**PNA2-H3**	1.8	1.2	65
**PNA2-N3**	1.4	1.2	80	**PNA2-A3**	1.1	1.1	98
**PNA2-G3**	2.6	1.2	44	**PNA2-P3**	2.8	2.3	155

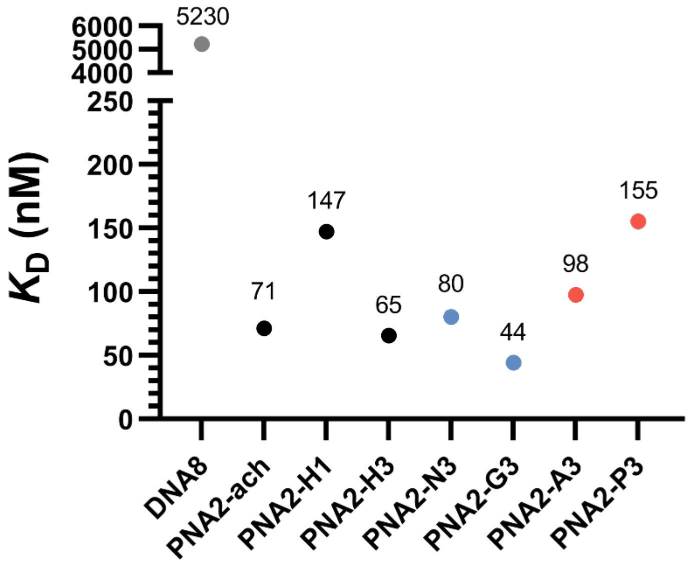

Figure S3. SPR dissociation equilibrium constants (K_D), association rates (k_{on}) and dissociation rates (k_{off}) for PNAs for **PNA3-AAmm-Bt** immobilized on the chip.

DNA6-Bt: 5'- CAC TTA GG- 3' 5'-Bt
DNA-8: 5'- CC TAA GTG- 3'
PNA2-ach: C-Ter: GTG AAT CC -Ac

PNA2-pc-ach: C-Ter: GTG$^+$ AAT CC$^+$ -Ac
PNA2-pc-A1: C-Ter: GTG$^+$ AA^{A}T CC$^+$ -Ac
PNA2-pc-A3: C-Ter: GT^{A}G$^+$ AA^{A}T C^{A}C$^+$ -Ac
PNA2-pc-H3: C-Ter: GT^{H}G$^+$ AA^{H}T C^{H}C$^+$ -Ac

N-7-MeG (G$^+$), G-clamp (C$^+$)

On chip DNA6-Bt	k_{on} x10^4 (M^{-1}s^{-1})	k_{off} x10^{-3} (s^{-1})	K_D (nM)		k_{on} x10^4 (M^{-1}s^{-1})	k_{off} x10^{-3} (s^{-1})	K_D (nM)
**DNA8**	39 ± 2	329 ± 6	8600 ± 2000	**PNA2-pc-H3**	7.9 ± 0.5	2.79 ± 0.06	35 ± 2
**PNA2-ach**	8.88 ± 0.01	113 ± 7	1280 ± 80	**PNA2-pc-A1**	4.5 ± 0.2	5.99 ± 0.01	135 ± 6
**PNA2-pc-ach**	38.8 ± 0.3	18.68 ± 0.08	48.1 ± 0.6	**PNA2-pc-A3**	2.12 ± 0.09	2.55 ± 0.06	121 ± 5

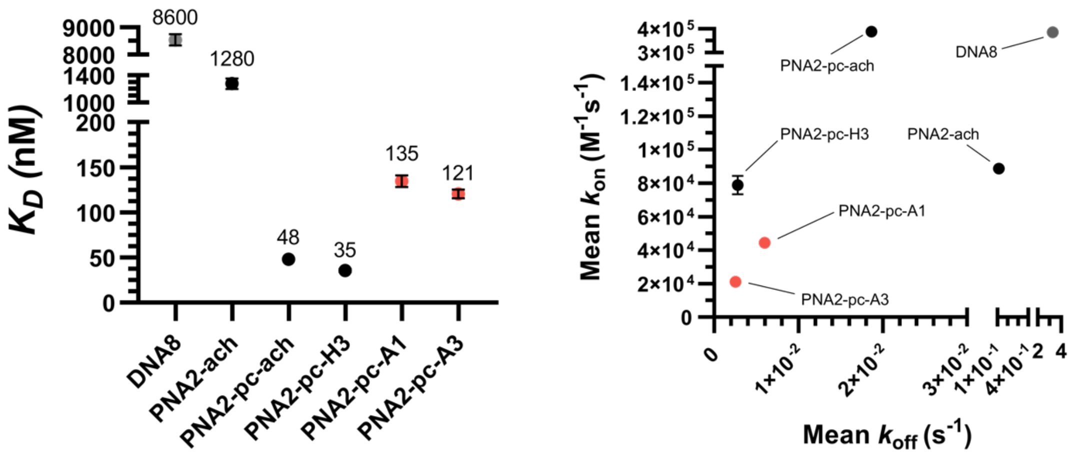

Figure S4. SPR dissociation equilibrium constants (K_D), association rates (k_{on}) and dissociation rates (k_{off}) for pc-PNAs for **DNA6-Bt** immobilized on the chip. Data presented as the average of duplicate measurements (n = 2) and reported as the mean ± 95% CI (z = 1.96). Plotted error bars represent the standard deviation (SD).

Abbreviations

AcN	Acetonitrile
Boc	Tert-butyloxycarbonyl
DCM	Dichloromethane
DHB	2,5-Dihydroxybenzoic acid
DIPEA	N,N-Diisopropylethylamine
DMF	Dimethylformamide
DMSO	Dimethyl sulfoxide
ESI	Electrospray ionization
EtOAc	Ethyl acetate
FITC	Fluorescein isothiocyanate
Fmoc	Fluorenylmethoxycarbonyl
HATU	Hexafluorophosphate Azabenzotriazole Tetramethyl Uronium
HPLC	High performance liquid chromatography
LC-MS	Liquid chromatography-mass spectrometry
MALDI	Matrix-assisted laser desorption/ionization
MS	Mass spectrometry
PEG	Polyethylene glycol
PNA	Peptide Nucleic Acid
TEA	Triethyl amine
TFA	Trifluoroacetic acid
THF	Tetrahydrofuran
THPTA	Tris(3-hydroxypropyltriazolylmethyl)amine

Synthesis of PNA oligomers

Synthesis of the reported PNA oligomers was performed starting from 5.0 mg of Fmoc-Rink-Amide PEG AM resin (0.33mmol/g, IrisBiotech) by iterative cycles of solid phase peptide synthesis following our previous reports. [1, 2]

Protocols for the synthesis of compounds Az-A/H/N/G/P

2-[2-[2-(2-Azidoethoxy)ethoxy]ethoxy]ethanol (Az-H)

Azido alcohol (**Az-H**) was synthesized according to the previous reported protocols.[3]

^{1}H NMR (400 MHz, $CDCl_3$): δ 3.87 (s, 1H), 3.70 – 3.66 (m, 2H), 3.64 – 3.60 (m, 12H), 3.60 – 3.53 (m, 2H), 3.35 (t, J = 5.1, 5.1 Hz, 2H).

2-[2-[2-(2-Azidoethoxy)ethoxy]ethoxy]acetic acid (Az-A)

Azido acetic acid (**Az-A**) was synthesized according to previous reported protocols.[3]

^{1}H NMR (400 MHz, $CDCl_3$): δ 9.69 (br. s, 1H), 4.15 (s, 2H), 3.73 – 3.61 (m, 10H), 3.35 (t, J = 5.2, 5.2 Hz, 2H).

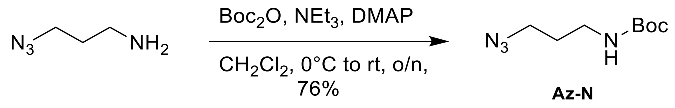

Tert-butyl (3-azidopropyl)carbamate (Az-N)

To a stirred solution of 3-azidopropan-1-amine (400mg, 4 mmol, 1 eq) in CH_2Cl_2 (5 mL) at 0°C, triethylamine (556 µL, 4 mmol, 1 eq) and 4-dimethylaminopyridine (24mg, 0.2 mmol, 0.05eq) were added. Then, di-tert-butyl dicarbonate (872 mg, 4 mmol, 1 eq) was added in one portion and the reaction mixture was left to warm to room temperature overnight. The reaction mixture was partitioned between Et_2O (60 mL) and NH_4Cl (sat.) (20 mL). The organic layer was separated, washed again with NH_4Cl (sat.) (2 x 20 mL), brine, dried over anhydrous Na_2SO_4, filtered, and concentrated in vacuo. The resulting crude residue was purified by column chromatography (10-30% EtOAc/pentane) to give **Az-N**.

Yield: 611mg (76%). Isolated as a pale yellow oil. Spectroscopic data in accordance with previous reports.[4]

R_f: 0.70 in 1/3 EtOAc/Pentane. UV inactive and stains yellow with $KMnO_4$ stain.

HR-MS/TOF-MS-ES+: *m/z* expected for $[M+Na]^+$: 223.1171, *m/z* found: 223.1181

^{1}H NMR (300 MHz, $CDCl_3$): δ 4.66 (br. s, 1H), 3.35 (t, J = 6.7 Hz, 2H), 3.26 – 3.15 (m, 2H), 1.76 (p, J = 6.7 Hz, 2H), 1.44 (s, 9H).

^{13}C NMR (75 MHz, $CDCl_3$): δ 156.1, 79.6, 49.3, 38.2, 29.4, 28.5.

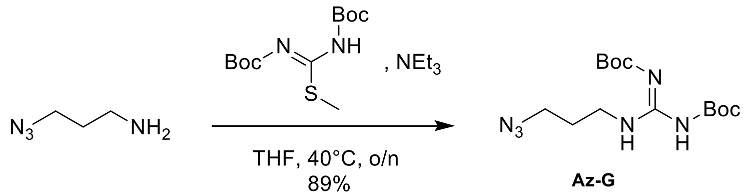

N^2, N^3-Bis(tert-butoxycarbonyl)-N^1-(3-azidopropyl)guanidine (Az-G)

To a stirred solution of N,N′-Di-Boc-*S*-methylisothiourea (300 mg, 1.03 mmol, 1 eq) in THF (3.2 mL) at room temperature, a solution of 3-azidopropan-1-amine (258 mg, 2.58 mmol, 2.5 eq) and triethylamine (430 µL, 3.09 mmol, 3 eq) in THF (2 mL) were added. The reaction mixture was left to stir at 40°C overnight. Then, concentrated in vacuo and the resulting crude residue was purified by column chromatography (10% EtOAc/pentane) to give **Az-G**.

Yield: 272mg (89%). Isolated as a white solid.

R_f: 0.30 in 10% EtOAc/Pentane. UV inactive and stains yellow with $KMnO_4$ stain.

HR-MS/TOF-MS-ES+: *m/z* expected for $[M+Na]^+$: 365.1913, *m/z* found: 365.1920

^{1}H NMR (300 MHz, $CDCl_3$): δ 11.48 (br. s, 1H), 8.42 (br. s, 1H), 3.51 (m, 2H), 3.38 (t, *J* = 6.8 Hz, 2H), 1.86 (p, *J* = 6.8 Hz, 2H), 1.49 (m, 18H).

^{13}C NMR (75 MHz, $CDCl_3$): δ 163.7, 156.4, 153.4, 83.4, 79.5, 49.3, 38.3, 28.6, 28.4, 28.2.

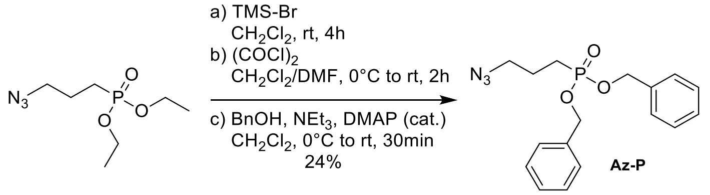

Dibenzyl (3-azidopropyl)phosphonate (Az-P)

To a stirred solution of diethyl (3-azidopropyl)phosphonate (300 mg, 1.4 mmol, 1 eq) in CH_2Cl_2 (1 mL) at room temperature, TMS-Br (648 µL, 4.8 mmol, 3.5 eq) were added and stirred under N_2 atmosphere for 4h. Then, excess of TMS-Br removed by CH_2Cl_2 co-evaporations (2 x 5 mL). The residual yellow oil was redissolved CH_2Cl_2 (1.5 mL) with 2 drops of DMF. Cooled to 0°C and $(COCl)_2$ (908 µL, 10.1 mmol, 7.4 eq) added dropwise under intert atmosphere (strong bubbling). The mixture was left to warm to room temperature for 2h before removing the excess $(COCl)_2$ by CH_2Cl_2 co-evaporations (2 x 5 mL). The residual orange oil was redissolved in CH_2Cl_2 (1 mL) and cooled to 0°, then a solution of benzyl alcohol (560 µL, 5.4 mmol, 4 eq), 4-dimethylaminopyridine (17 mg, 0.1 mmol, 0.1 eq) and triethylamine (1.14 mL, 8.2 mmol, 6 eq) in CH_2Cl_2 (1.5 mL) was dropwise added under inert atmosphere (strong exotherm). The reaction mixture was left to warm to room temperature for 30min, partitioned between Et_2O (60 mL) and NH_4Cl (sat.) (20 mL). The organic layer was separated, washed again with NH_4Cl (sat.) (2 x 20 mL), water (1 x 20 mL), 1M NaOH (3 x 20 mL), brine, dried over anhydrous Na_2SO_4, filtered, and concentrated in vacuo. The resulting crude residue was purified by column chromatography (10-30% acetone/pentane) to give **Az-p**.

Yield: 121mg (24%). Isolated as a pale yellow oil.

R_f: 0.60 in 30% acetone/pentane. UV active and stains yellow with $KMnO_4$ stain.

HR-MS/TOF-MS-ES+: *m/z* expected for $[M+Na]^+$: 368.1140, *m/z* found: 368.1147

^{1}H NMR (400 MHz, CDCl$_3$): δ 7.42 – 7.29 (m, 10H), 5.10 – 4.92 (m, 4H), 3.43 – 3.19 (m, 2H), 2.24 – 1.67 (m, 4H).

^{13}C NMR (101 MHz, CDCl$_3$): δ 136.4, 136.4, 128.8, 128.7, 128.1, 67.5, 67.4, 51.6, 51.4, 33.7, 33.5, 26.0, 25.9, 25.7, 24.3, 24.2, 22.7, 22.5, 22.4.

^{31}P NMR (121 MHz, CDCl$_3$): δ 34.8, 32.0 (major), 31.7.

Characterisation of PNA oligomers

PNA1- ach-D: C-Ter: Lys(ACT GGG ATT CAC -Ac)-Dabcyl

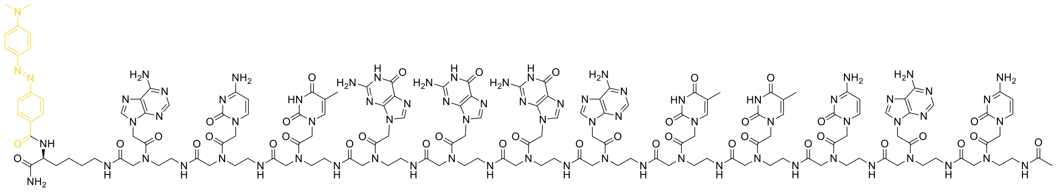

Chemical Formula: $C_{152}H_{189}N_{75}O_{39}$ **Exact Mass:** 3688.511 **Molecular Weight:** 3690.670

LC-MS-ESI+: *m/z* expected for $[M+6H]^{6+}$: 616.1, *m/z* found: 616.2; *m/z* expected for $[M+5H]^{5+}$: 739.1, *m/z* found: 739.1; *m/z* expected for $[M+4H]^{4+}$: 923.6, *m/z* found: 923.4; *m/z* expected for $[M+3H]^{3+}$: 1231.2, *m/z* found: 1231.0.

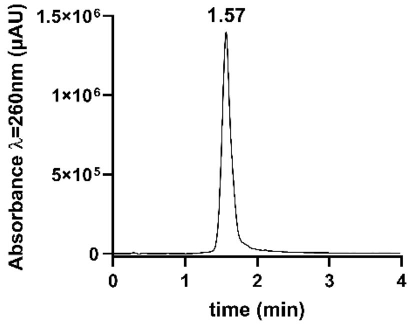

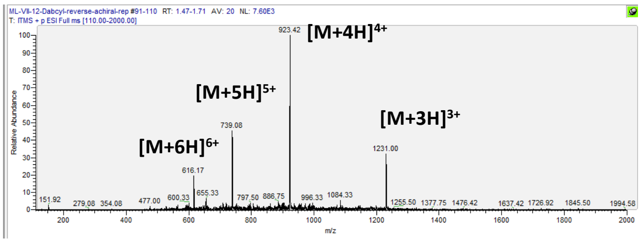

MALDI-TOF-MS: *m/z* expected for $[M+H]^{+}$: 3691.511, *m/z* found: 3692.187. *In all PNAs containing Dabcyl quencher have a secondary peak with a -132 m/z corresponding to a fragmentation of the quencher.*

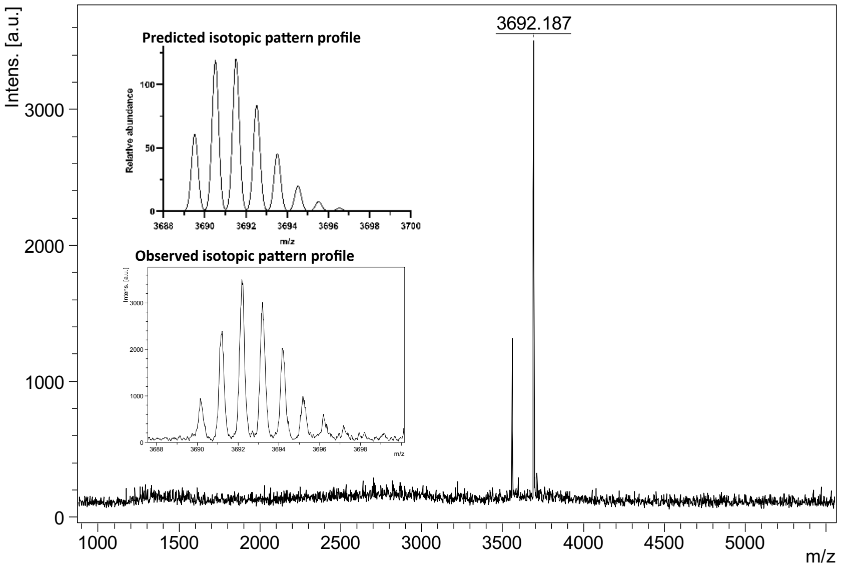

PNA1-ach-F: C-Ter: GTG AAT CCC AGT-Lys(FITC)-Ac

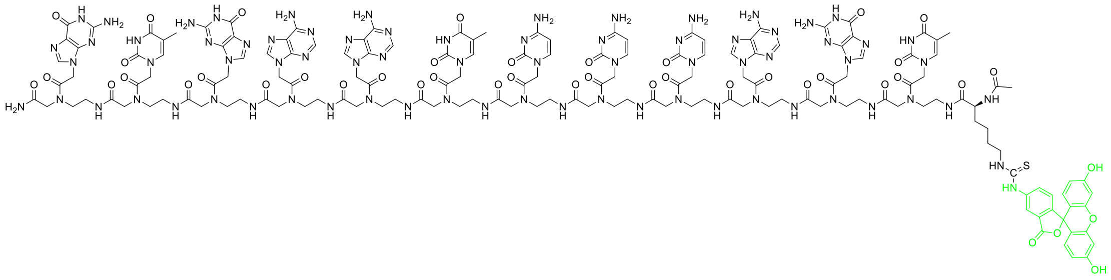

Chemical Formula: $C_{158}H_{187}N_{73}O_{43}S$ **Exact Mass:** 3826.441 **Molecular Weight:** 3828.762

LC-MS-ESI+: *m/z* expected for $[M+4H]^{4+}$: 958.1, *m/z* found: 958.3; *m/z* expected for $[M+3H]^{3+}$: 1277.2, *m/z* found: 1277.3; *m/z* expected for $[M+2H]^{2+}$: 1915.2, *m/z* found: 1914.7. (*0.1% aqueous TFA solution and 0.1% TFA in HPLC grade acetonitrile used as eluents.*)

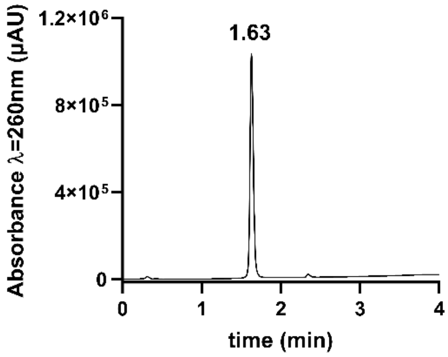

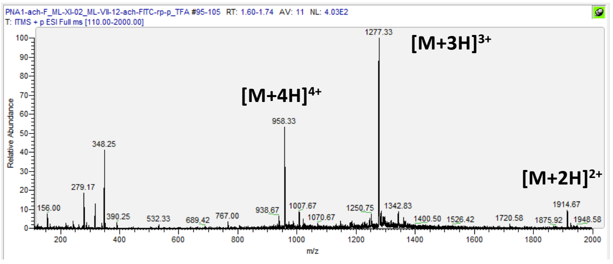

MALDI-TOF-MS: *m/z* expected for $[M+H]^+$: 3829.438, *m/z* found: 3830.111

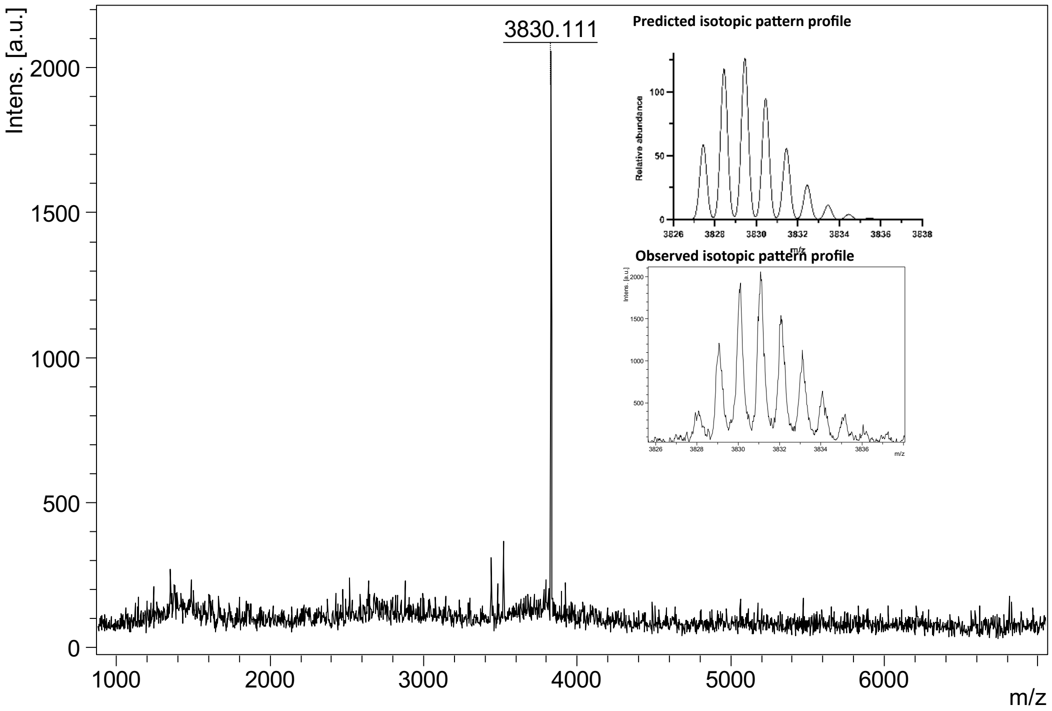

PNA1-H1-D: C-Ter: Lys(ACT GG^{H}G ATT CAC -Ac)-Dabcyl

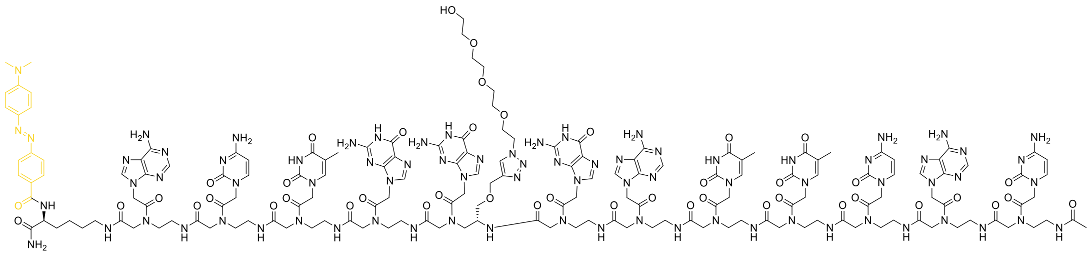

Chemical Formula: $C_{164}H_{210}N_{78}O_{44}$ **Exact Mass:** 3975.659 **Molecular Weight:** 3977.986

LC-MS-ESI+: *m/z* expected for $[M+6H]^{6+}$: 664.0, *m/z* found: 664.2; *m/z* expected for $[M+5H]^{5+}$: 796.5, *m/z* found: 796.7; *m/z* expected for $[M+4H]^{4+}$: 995.4, *m/z* found: 995.4; *m/z* expected for $[M+3H]^{3+}$: 1326.9, *m/z* found: 1326.9.

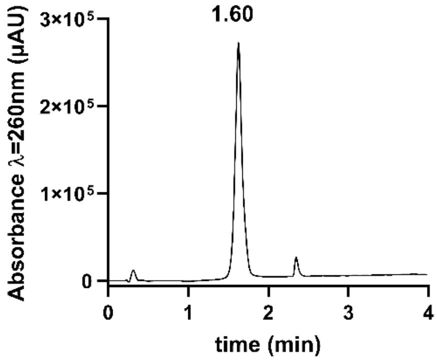

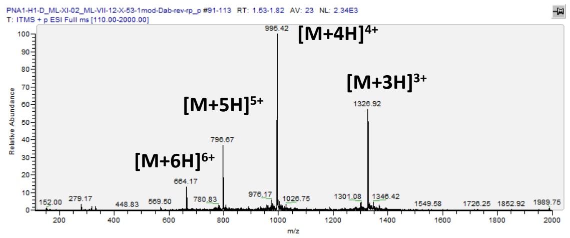

MALDI-TOF-MS: *m/z* expected for $[M+Na]^{+}$: 4000.656 *m/z* found: 4001.175. *In all PNAs containing Dabcyl quencher have a secondary peak with a -132 m/z corresponding to a fragmentation of the quencher.*

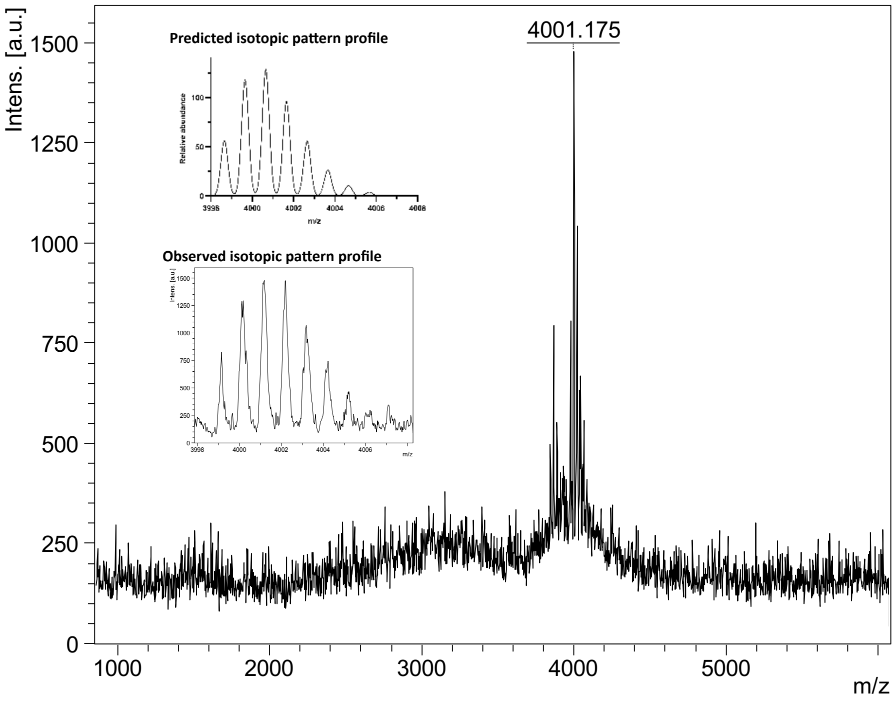

PNA1-H1-F: C-Ter: GTG AAT CC^{H}C AGT-Lys(FITC)-Ac

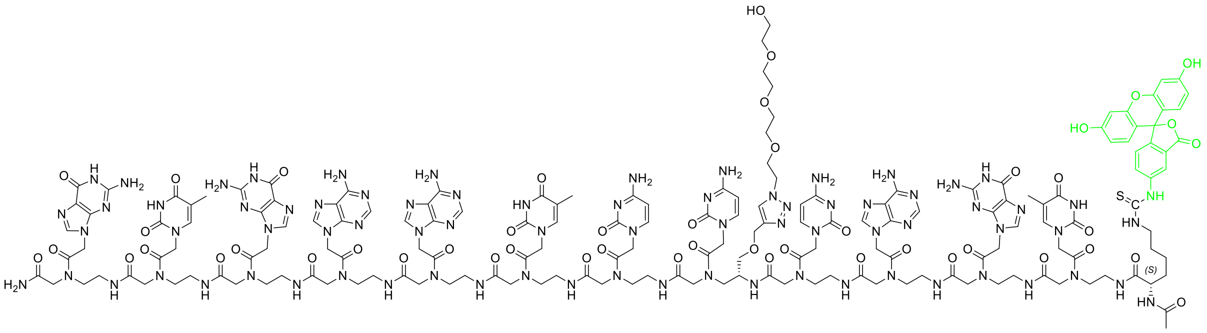

Chemical Formula: $C_{170}H_{208}N_{76}O_{48}S$ **Exact Mass:** 4113.589 **Molecular Weight:** 4116.078

LC-MS-ESI+: *m/z* expected for $[M+5H]^{5+}$: 824.1, *m/z* found: 824.7; *m/z* expected for $[M+4H]^{4+}$: 1029.9, *m/z* found: 1030.1; *m/z* expected for $[M+3H]^{3+}$: 1372.9, *m/z* found: 1372.9. (*0.1% aqueous TFA solution and 0.1% TFA in HPLC grade acetonitrile used as eluents.*)

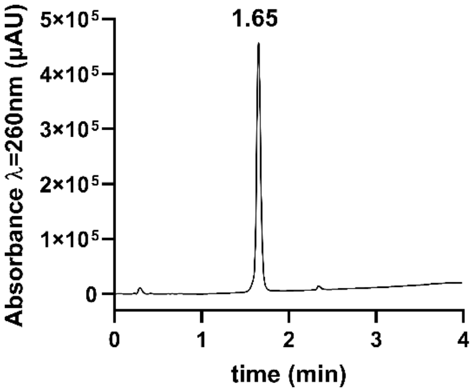

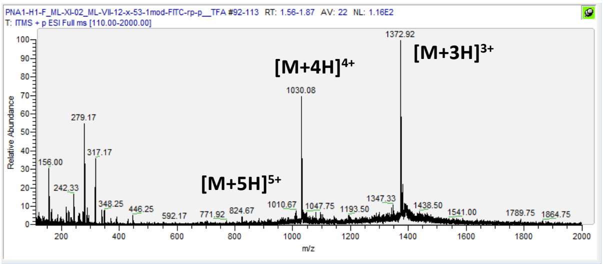

MALDI-TOF-MS: *m/z* expected for $[M+H]^{+}$: 4116.604, *m/z* found: 4116.239

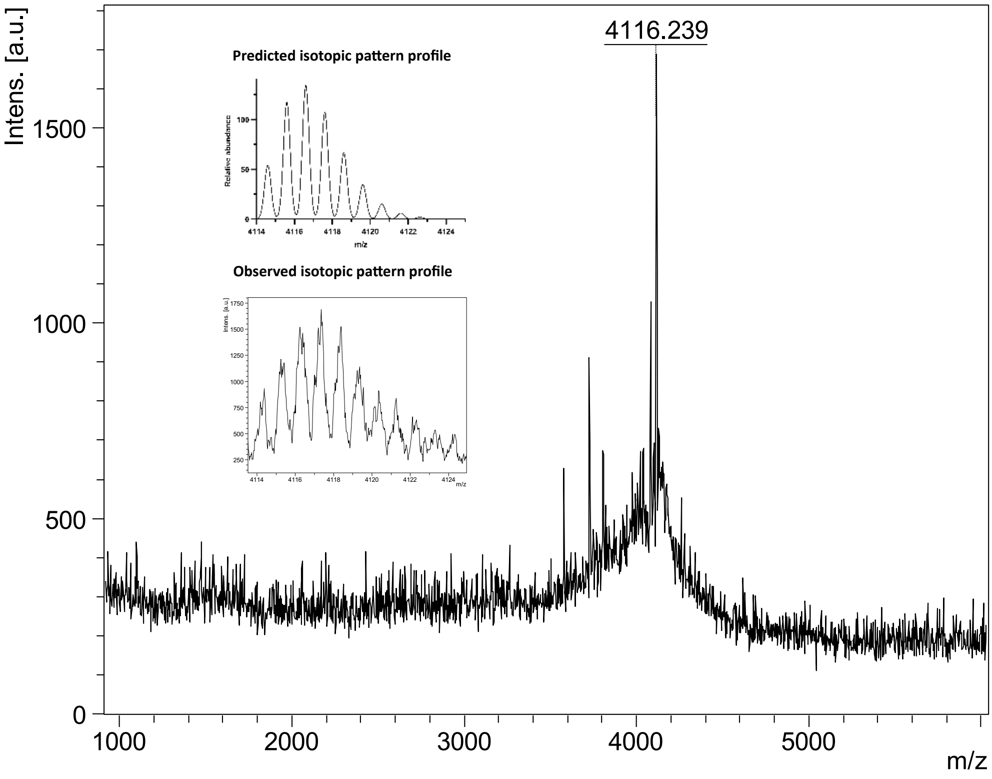

PNA1-H4-D: C-Ter: Lys(AC^{H}T GG^{H}G AT^{H}T CA^{H}C -Ac)-Dabcyl

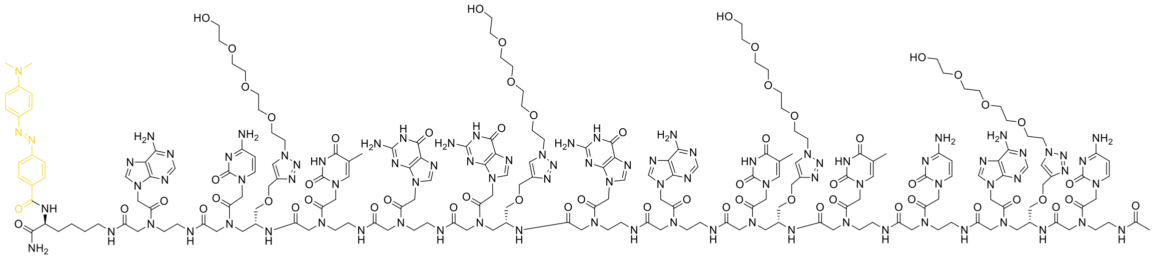

Chemical Formula: $C_{200}H_{273}N_{87}O_{59}$ **Exact Mass:** 4837.104 **Molecular Weight:** 4839.934

LC-MS-ESI+: *m/z* expected for $[M+6H]^{6+}$: 807.5, *m/z* found: 807.8 *m/z* expected for $[M+5H]^{5+}$: 968.8, *m/z* found: 968.9; *m/z* expected for $[M+4H]^{4+}$: 1210.8, *m/z* found: 1210.9; *m/z* expected for $[M+3H]^{3+}$: 1614.0, *m/z* found: 1613.9.

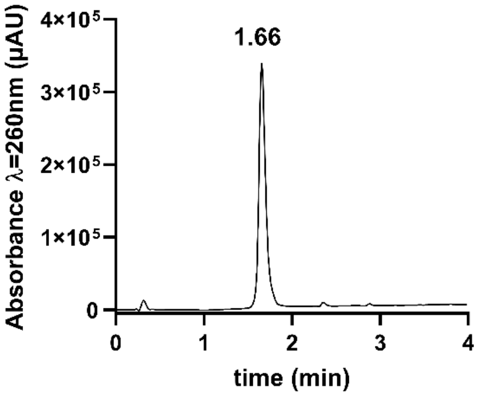

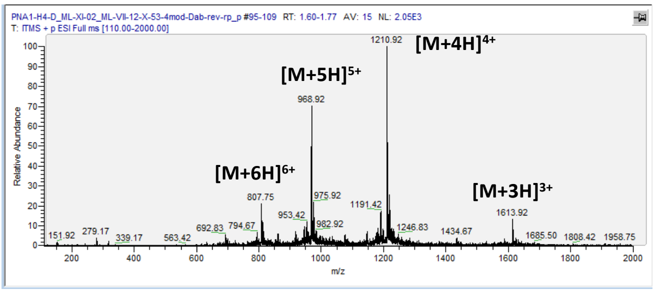

MALDI-TOF-MS: *m/z* expected for $[M+H]^{+}$: 4840.114, *m/z* found: 4840.054. *In all PNAs containing Dabcyl quencher have a secondary peak with a -132 m/z corresponding to a fragmentation of the quencher.*

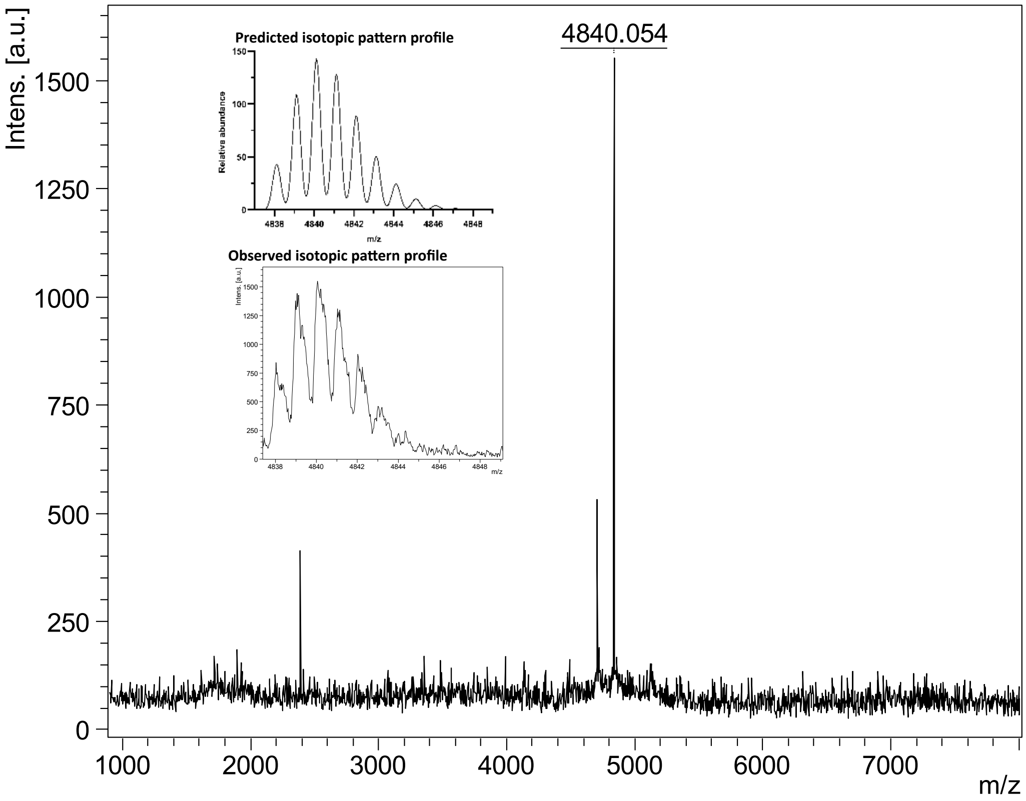

PNA1-H4-F: C-Ter: GT^{H}G AA^{H}T CC^{H}C AG^{H}T-Lys(FITC)-Ac

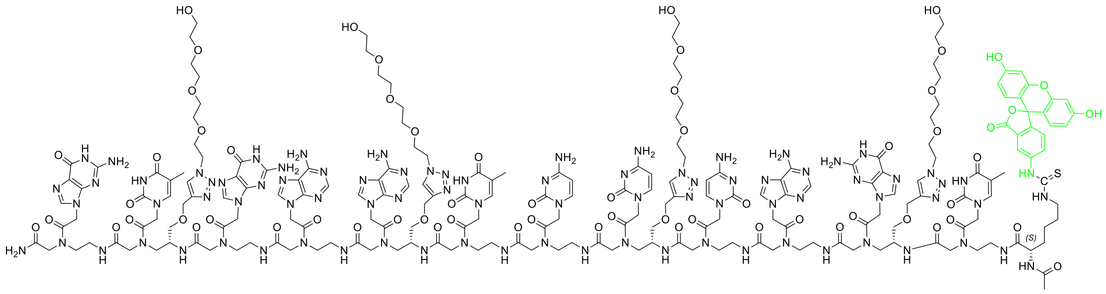

Chemical Formula: $C_{206}H_{271}N_{85}O_{63}S$ **Exact Mass:** 4975.034 **Molecular Weight:** 4978.026

LC-MS-ESI+: *m/z* expected for $[M+6H]^{6+}$: 830.5, *m/z* found: 830.5; *m/z* expected for $[M+5H]^{5+}$: 996.4, *m/z* found: 968.5; *m/z* expected for $[M+4H]^{4+}$: 1245.3, *m/z* found: 1245.4; *m/z* expected for $[M+3H]^{3+}$: 1660.0, *m/z* found: 1660.3.

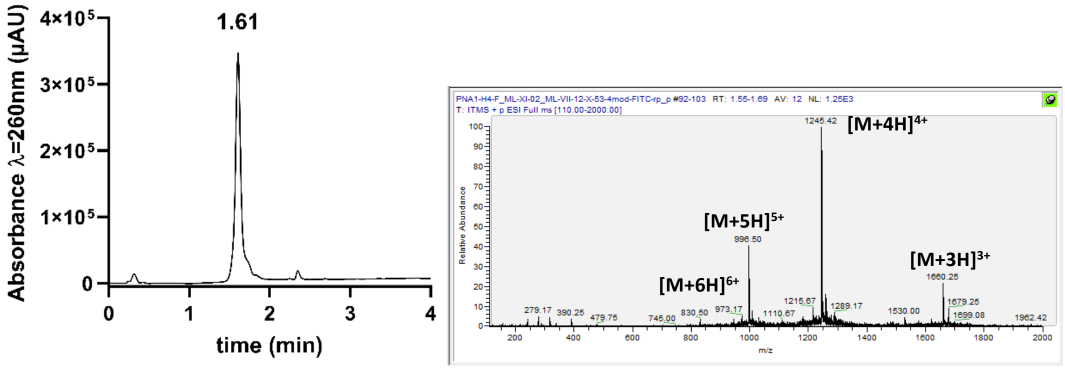

HR-MS/TOF-MS-ES+: *m/z* expected for $[M+3H]^{3+}$: 1660.0208, *m/z* found: 1660.0006; *m/z* expected for $[M+4H]^{4+}$: 1245.2675, *m/z* found: 1245.2483; *m/z* expected for $[M+5H]^{5+}$: 996.4156, *m/z* found: 996.6104

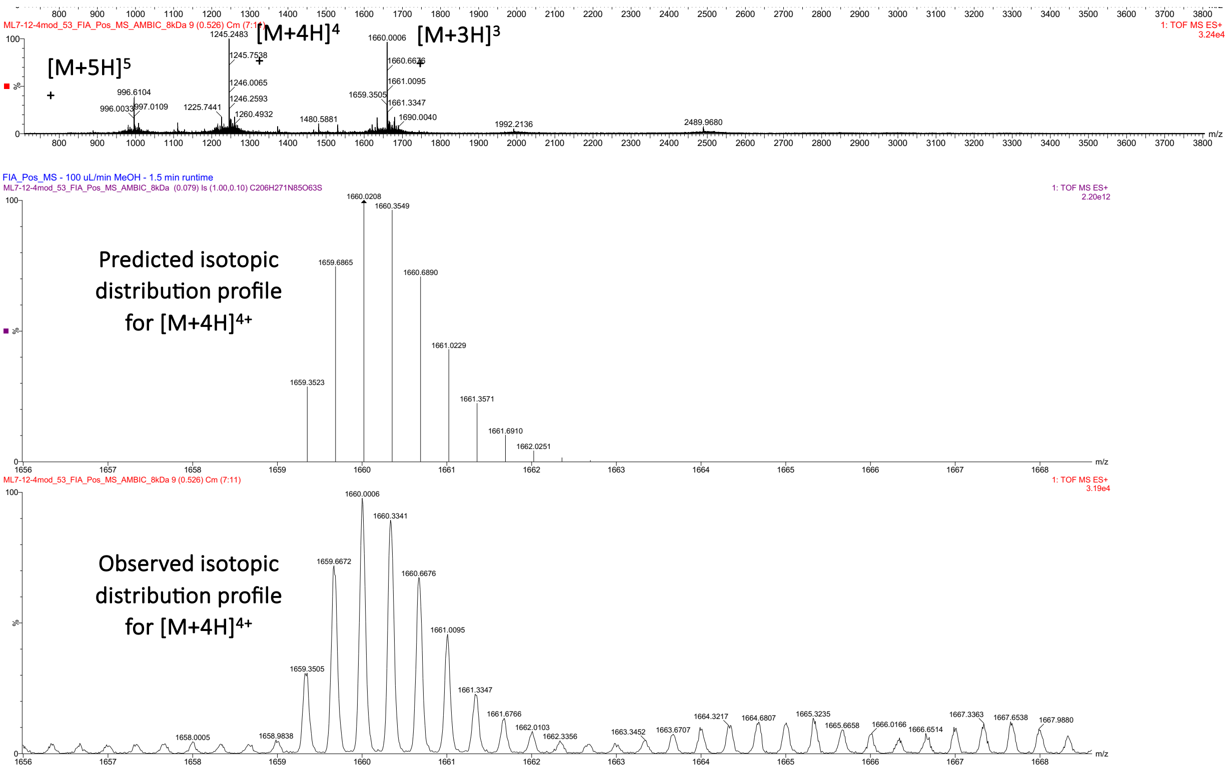

PNA1-A1-D: C-Ter: Lys(ACT GG^{A}G ATT CAC -Ac)-Dabcyl

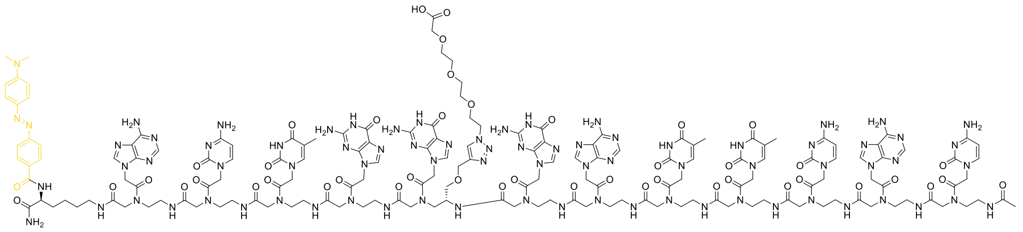

Chemical Formula: $C_{164}H_{208}N_{78}O_{45}$ **Exact Mass:** 3989.639 **Molecular Weight:** 3991.969

LC-MS-ESI+: *m/z* expected for $[M+6H]^{6+}$: 666.3, *m/z* found: 666.4; *m/z* expected for $[M+5H]^{5+}$: 799.3, *m/z* found: 799.5; *m/z* expected for $[M+4H]^{4+}$: 998.9, *m/z* found: 990.0; *m/z* expected for $[M+3H]^{3+}$: 1331.6, *m/z* found: 1331.5.

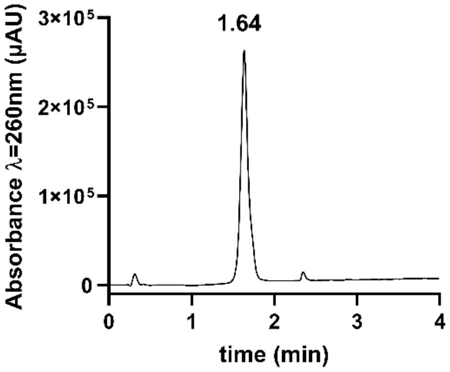

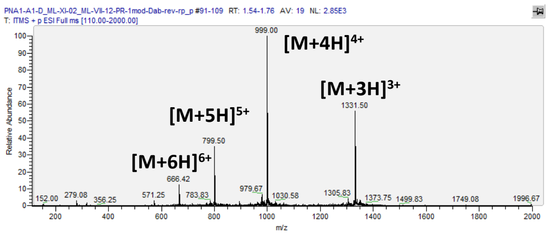

MALDI-TOF-MS: *m/z* expected for $[M+Na]^{+}$: 4014.641, *m/z* found: 4015.324. *In all PNAs containing Dabcyl quencher have a secondary peak with a -132 m/z corresponding to a fragmentation of the quencher.*

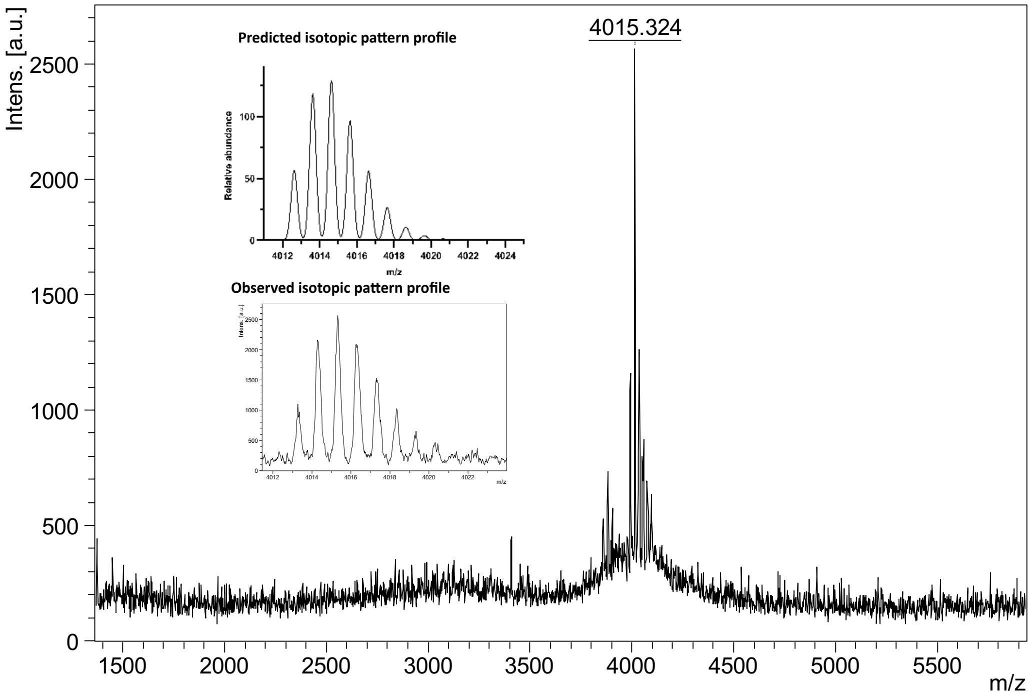

PNA1-A1-F: C-Ter: GTG AAT CC^{A}C AGT-Lys(FITC)-Ac

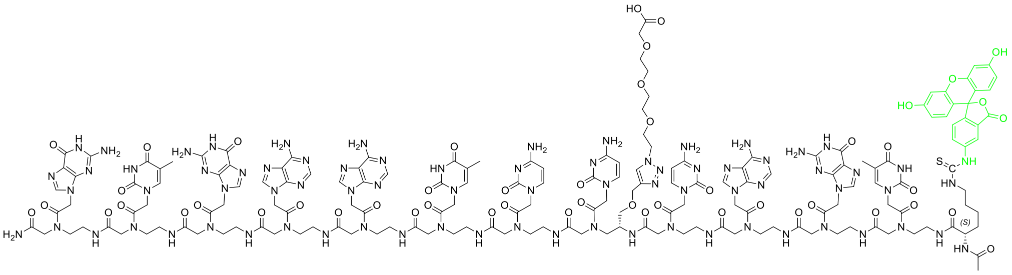

Chemical Formula: $C_{170}H_{206}N_{76}O_{49}S$ **Exact Mass:** 4127.568 **Molecular Weight:** 4130.061

LC-MS-ESI+: *m/z* expected for $[M+5H]^{5+}$: 826.9, *m/z* found: 827.0; *m/z* expected for $[M+4H]^{4+}$: 1033.4, *m/z* found: 1033.5; *m/z* expected for $[M+3H]^{3+}$: 1377.5, *m/z* found: 1377.7. (*0.1% aqueous TFA solution and 0.1% TFA in HPLC grade acetonitrile used as eluents.*)

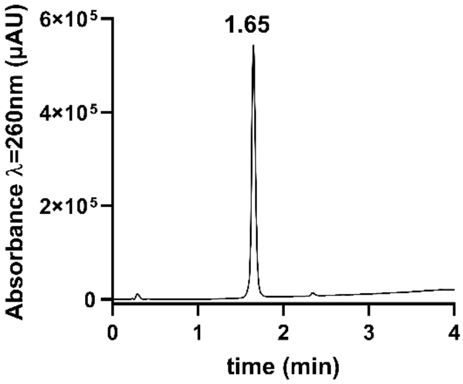

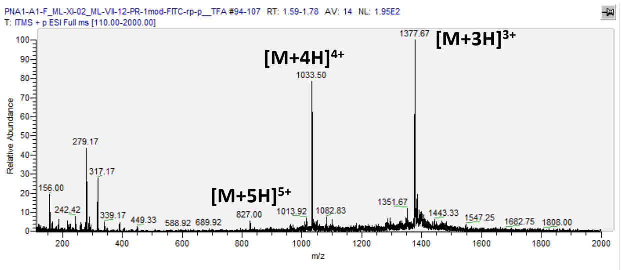

MALDI-TOF-MS: *m/z* expected for $[M+H]^{+}$: 4130.594, *m/z* found: 4130.669.

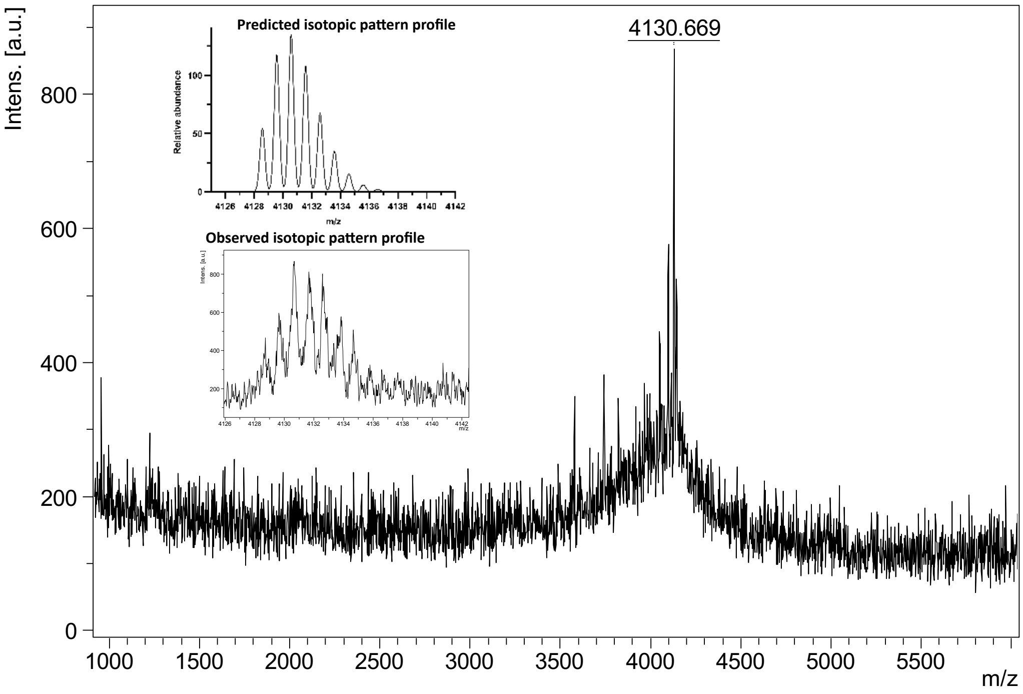

HR-MS/TOF-MS-ES+: *m/z* expected for $[M+4H]^{4+}$: 1033.4011, *m/z* found: 1033.3933

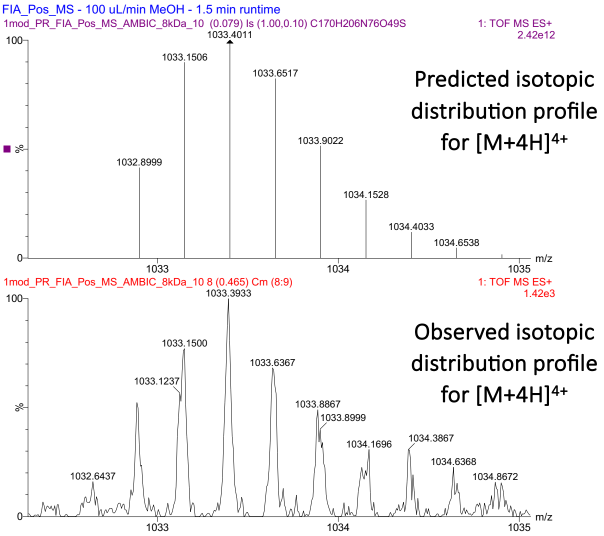

PNA1-A4-D: C-Ter: Lys(AC^{A}T GG^{A}G AT^{A}T CA^{A}C -Ac)-Dabcyl

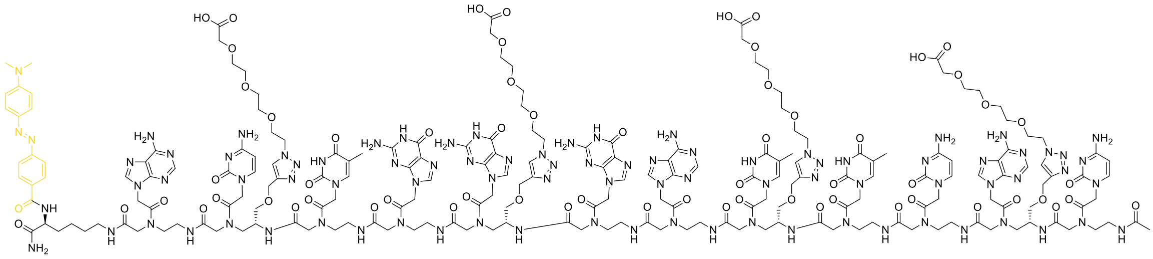

Chemical Formula: $C_{200}H_{265}N_{87}O_{63}$ **Exact Mass:** 4893.021 **Molecular Weight:** 4895.866

LC-MS-ESI+: *m/z* expected for $[M+6H]^{6+}$: 816.8, *m/z* found: 817.1; *m/z* expected for $[M+5H]^{5+}$: 980.0, *m/z* found: 980.3; *m/z* expected for $[M+4H]^{4+}$: 1224.8, *m/z* found: 1225.0; *m/z* expected for $[M+3H]^{3+}$: 1632.7, *m/z* found: 1632.9.

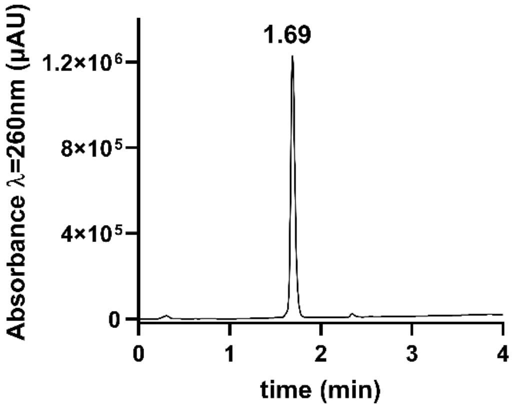

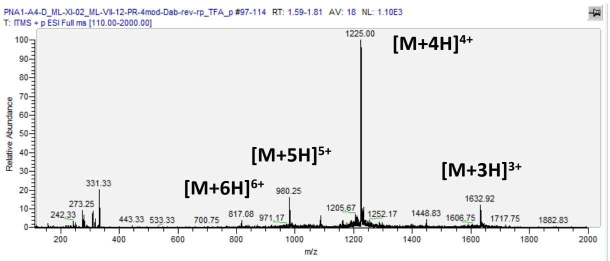

MALDI-TOF-MS: *m/z* expected for $[M+H]^+$: 4896.017, *m/z* found: 4896.514. *In all PNAs containing Dabcyl quencher have a secondary peak with a -132 m/z corresponding to a fragmentation of the quencher.*

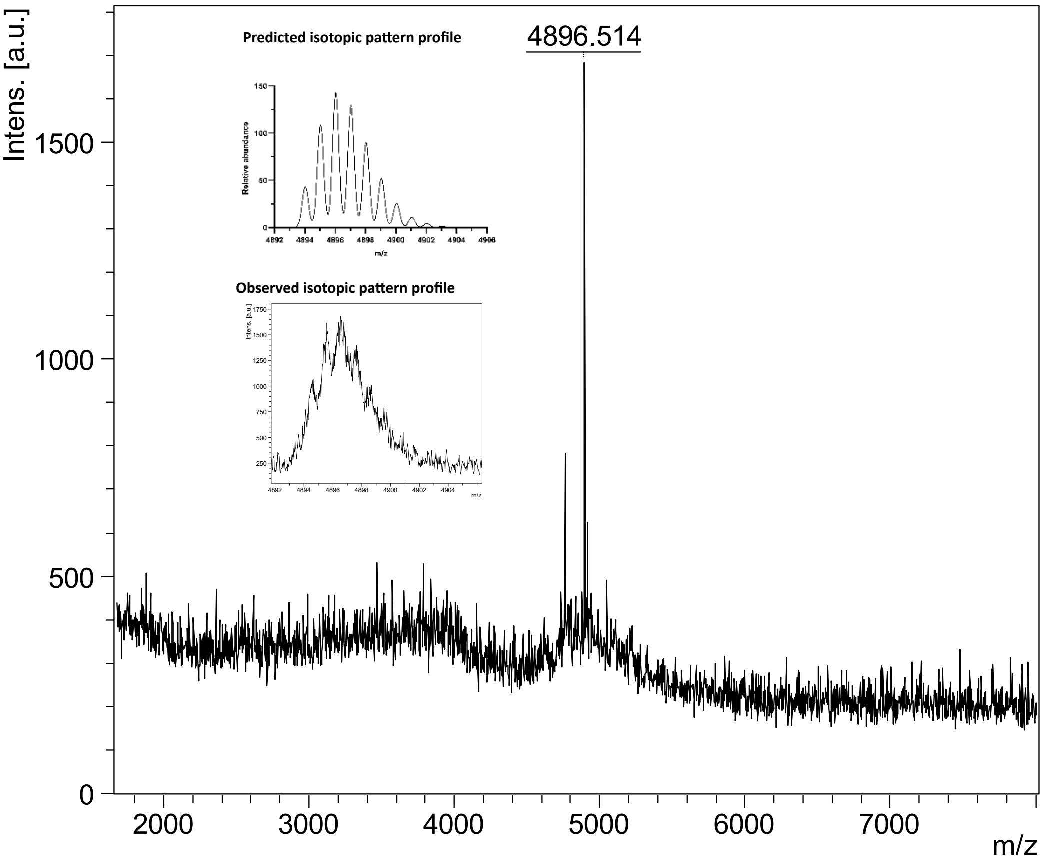

PNA1-A4-F: C-Ter: GT^{A}G AA^{A}T CC^{A}C AG^{A}T-Lys(FITC)-Ac

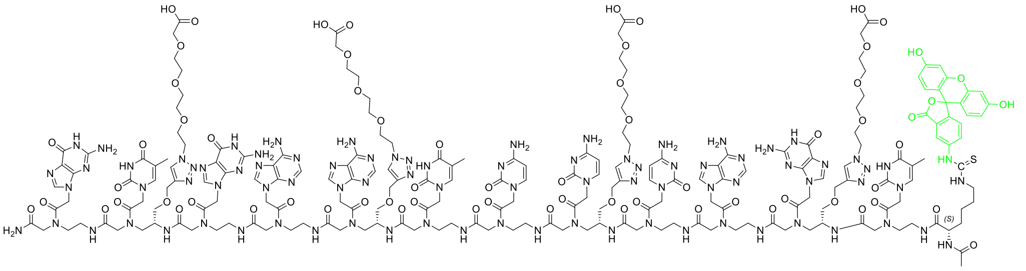

Chemical Formula: $C_{206}H_{263}N_{85}O_{67}S$ **Exact Mass:** 5030.951 **Molecular Weight:** 5033.958

LC-MS-ESI+: *m/z* expected for $[M+6H]^{6+}$: 839.8, *m/z* found: 840.0 *m/z* expected for $[M+5H]^{5+}$: 1007.6, *m/z* found: 1007.7; *m/z* expected for $[M+4H]^{4+}$: 1259.2, *m/z* found: 1259.3; *m/z* expected for $[M+3H]^{3+}$: 1678.7, *m/z* found: 1678.8. (*0.1% aqueous TFA solution and 0.1% TFA in HPLC grade acetonitrile used as eluents.*)

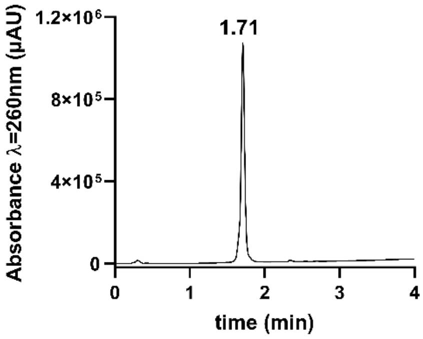

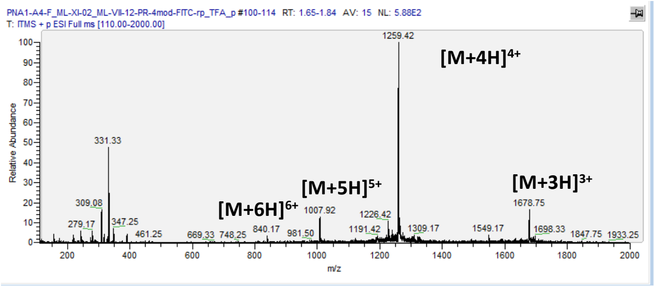

HR-MS/TOF-MS-ES+: *m/z* expected for $[M+3H]^{3+}$: 1678.6598, *m/z* found: 1678.6427; *m/z* expected for $[M+4H]^{4+}$: 1259.2467, *m/z* found: 1259.2367; *m/z* expected for $[M+5H]^{5+}$: 1007.5990, *m/z* found: 1007.5889

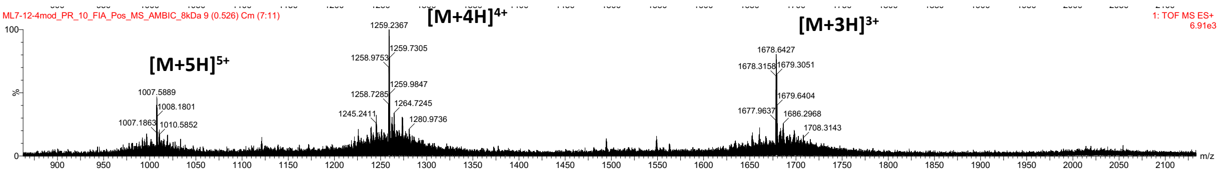

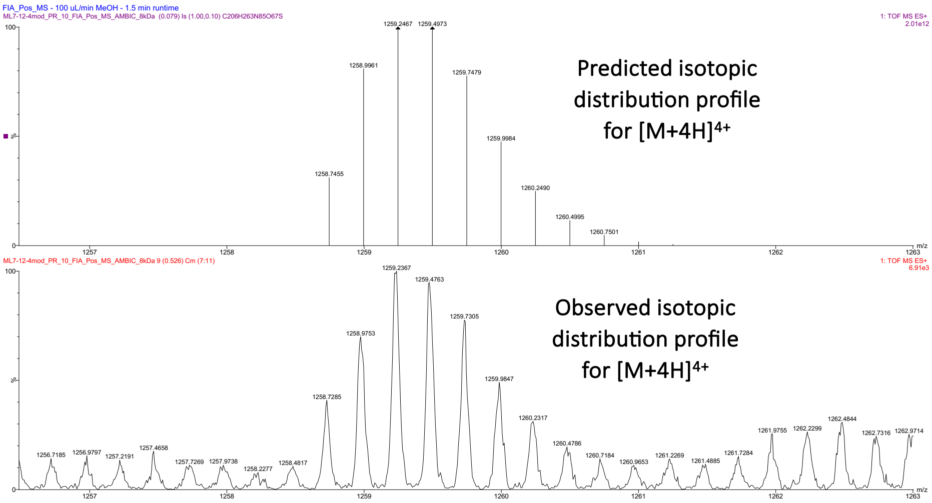

PNA1-N1-D: C-Ter: Lys(ACT GG^{N}G ATT CAC -Ac)-Dabcyl

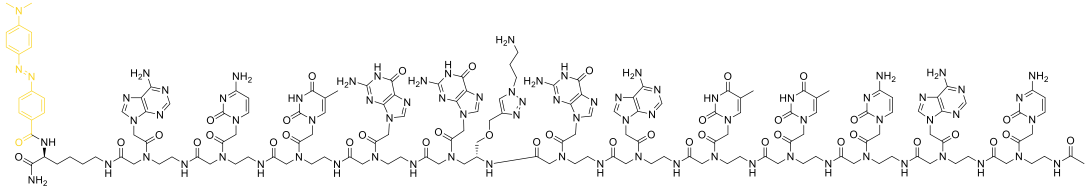

Chemical Formula: $C_{159}H_{201}N_{79}O_{40}$ **Exact Mass:** 3856.612 **Molecular Weight:** 3858.870

LC-MS-ESI+: *m/z* expected for $[M+6H]^{6+}$: 644.1, *m/z* found: 644.2; *m/z* expected for $[M+5H]^{5+}$: 772.7, *m/z* found: 772.6; *m/z* expected for $[M+4H]^{4+}$: 965.7, *m/z* found: 965.5; *m/z* expected for $[M+3H]^{3+}$: 1287.1, *m/z* found: 1267.1.

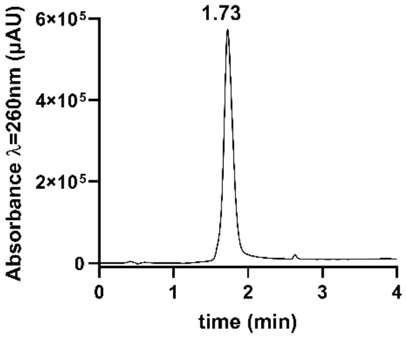

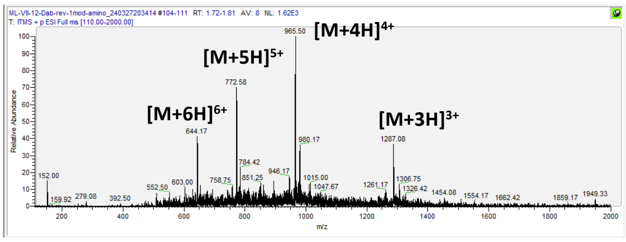

MALDI-TOF-MS: *m/z* expected for $[M+H]^{+}$: 3859.633, *m/z* found: 3860.161. *In all PNAs containing Dabcyl quencher have a secondary peak with a -132 m/z corresponding to a fragmentation of the quencher.*

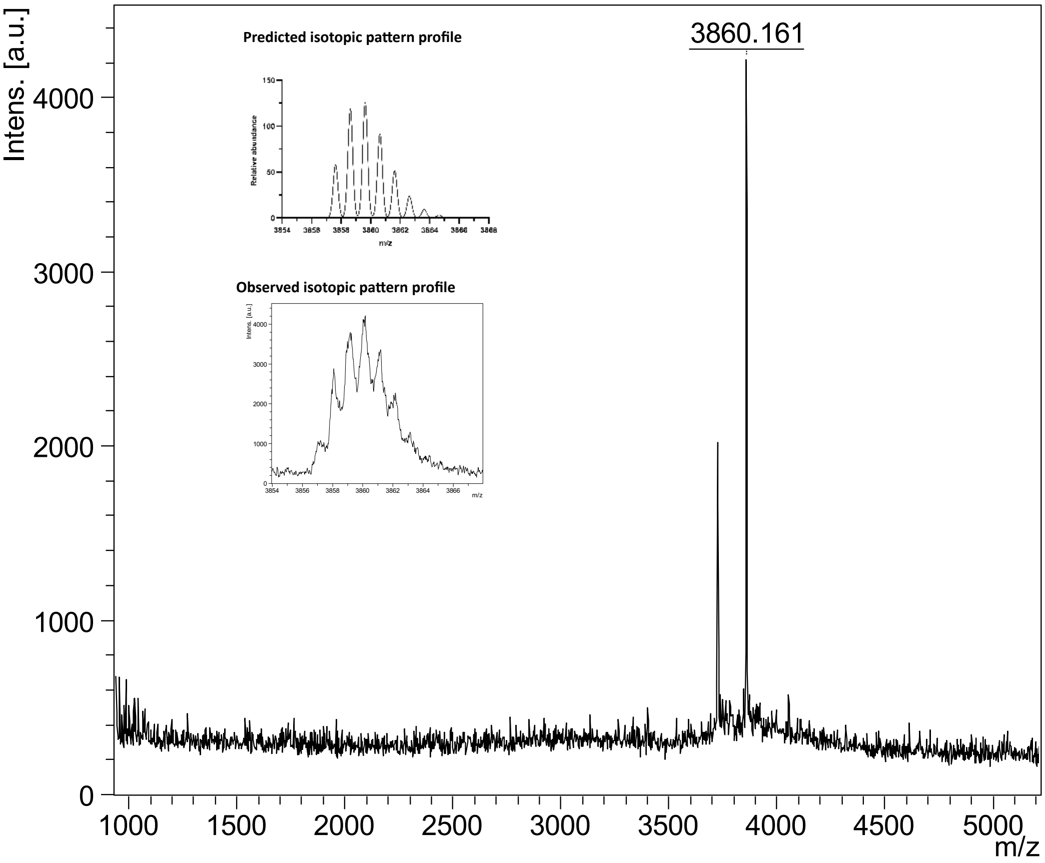

PNA1-N1-F: C-Ter: GTG AAT CC^{N}C AGT-Lys(FITC)-Ac

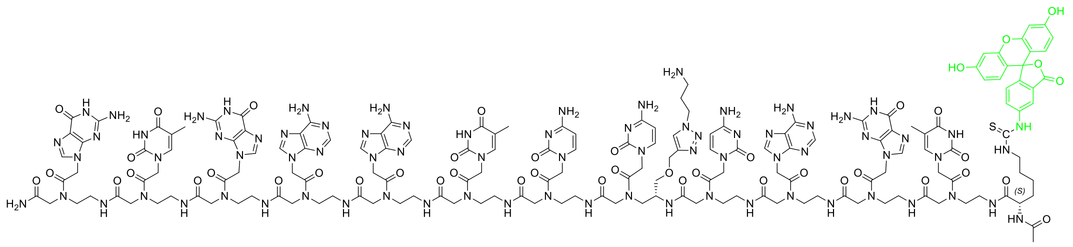

Chemical Formula: $C_{165}H_{199}N_{77}O_{44}S$ **Exact Mass:** 3994.542 **Molecular Weight:** 3996.962

LC-MS-ESI+: *m/z* expected for $[M+5H]^{5+}$: 800.3, *m/z* found: 800.4; *m/z* expected for $[M+4H]^{4+}$: 1000.1, *m/z* found: 1000.2; *m/z* expected for $[M+3H]^{3+}$: 1333.2, *m/z* found: 1333.2.

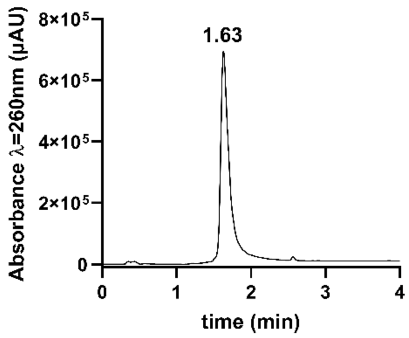

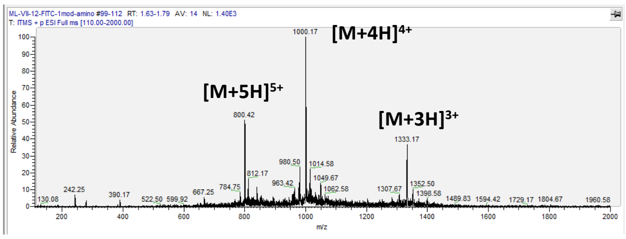

MALDI-TOF-MS: *m/z* expected for $[M+H]^{+}$: 3997.550, *m/z* found: 3997.123.

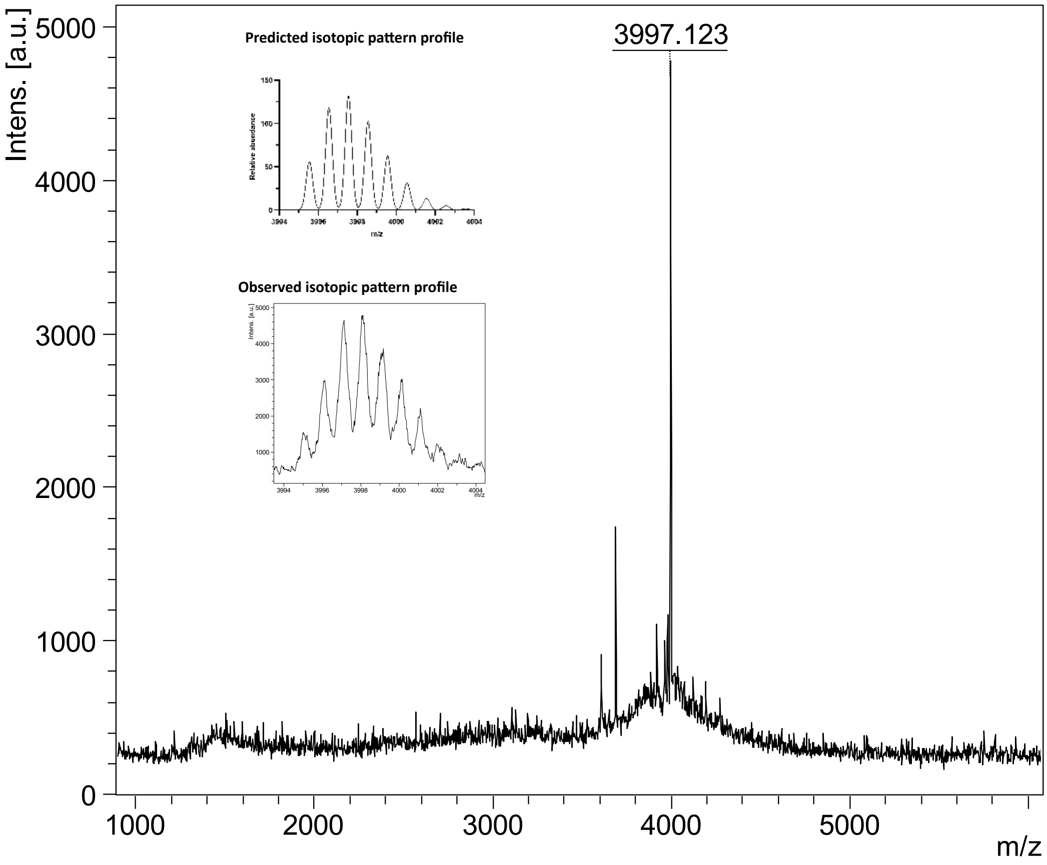

PNA1-N4-D: C-Ter: Lys(AC^{N}T GG^{N}G AT^{N}T CA^{N}C -Ac)-Dabcyl

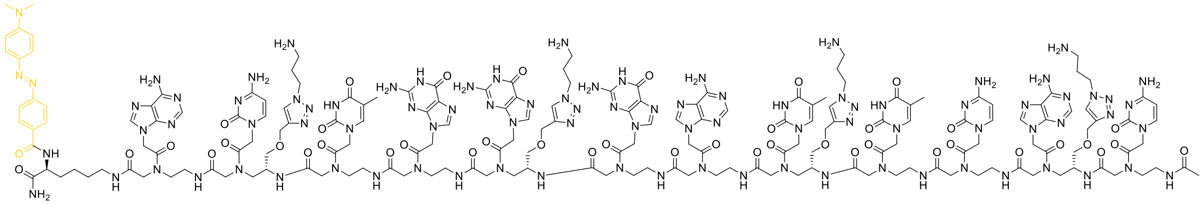

Chemical Formula: $C_{180}H_{237}N_{91}O_{43}$ **Exact Mass:** 4360.916 **Molecular Weight:** 4363.470

LC-MS-ESI+: *m/z* expected for $[M+7H]^{7+}$: 624.3, *m/z* found: 624.4; *m/z* expected for $[M+6H]^{6+}$: 728.2, *m/z* found: 728.3; *m/z* expected for $[M+5H]^{5+}$: 873.6, *m/z* found: 873.8; *m/z* expected for $[M+4H]^{4+}$: 1091.7, *m/z* found: 1091.8.

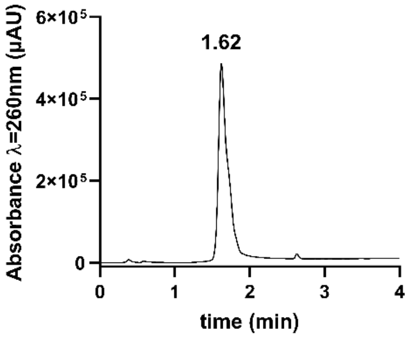

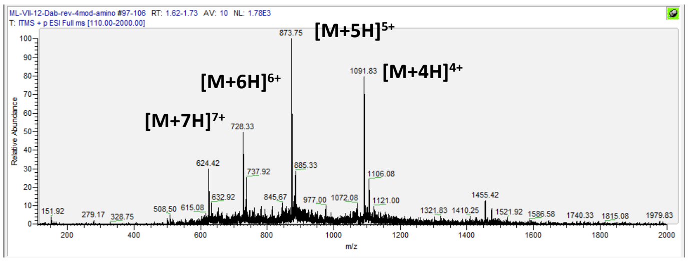

MALDI-TOF-MS: *m/z* expected for $[M+H]^{+}$: 4363.937, *m/z* found: 4363.583. *In all PNAs containing Dabcyl quencher have a secondary peak with a -132 m/z corresponding to a fragmentation of the quencher.*

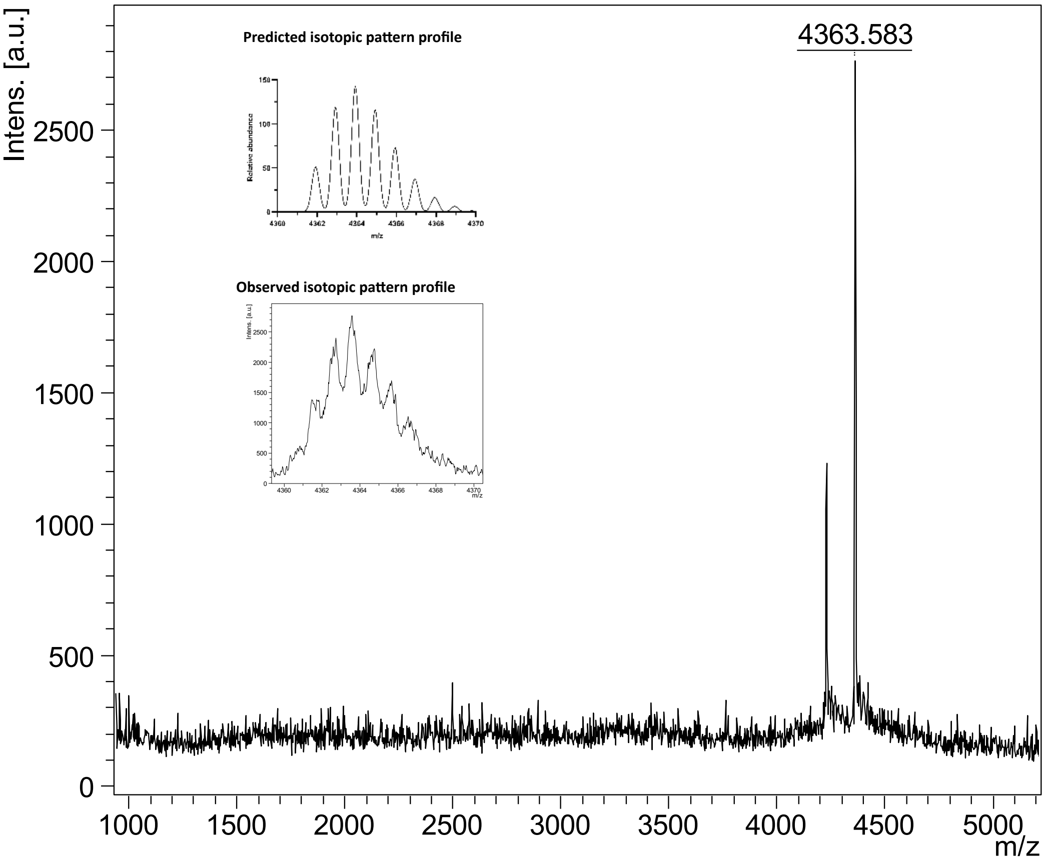

PNA1-N4-F: C-Ter: GT^{N}G AA^{N}T CC^{N}C AG^{N}T-Lys(FITC)-Ac

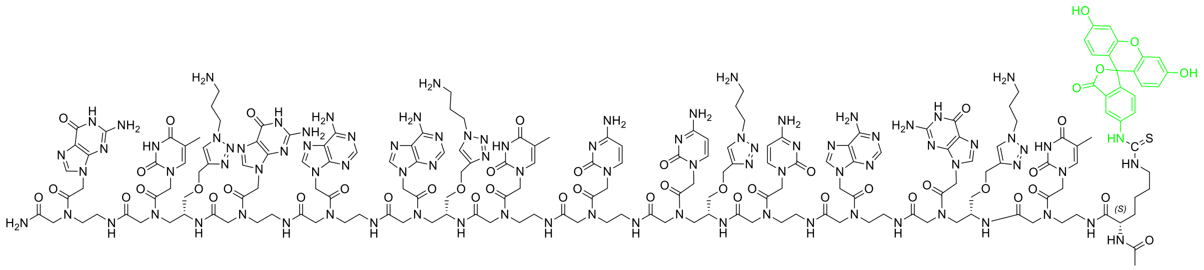

Chemical Formula: $C_{186}H_{235}N_{89}O_{47}S$ **Exact Mass:** 4498.846 **Molecular Weight:** 4501.562

LC-MS-ESI+: *m/z* expected for $[M+6H]^{6+}$: 751.1, *m/z* found: 751.3; *m/z* expected for $[M+5H]^{5+}$: 901.2, *m/z* found: 901.3; *m/z* expected for $[M+4H]^{4+}$: 1126.2, *m/z* found: 1126.3; *m/z* expected for $[M+3H]^{3+}$: 1501.3, *m/z* found: 1501.3.

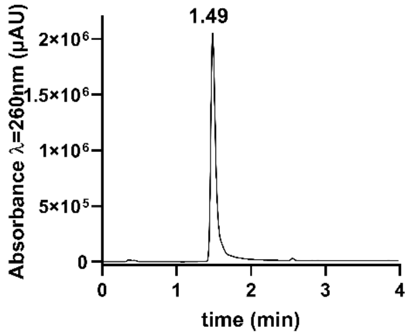

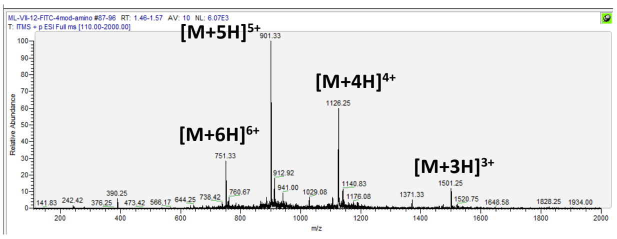

MALDI-TOF-MS: *m/z* expected for $[M+H]^+$: 4501.872, *m/z* found: 4501.810.

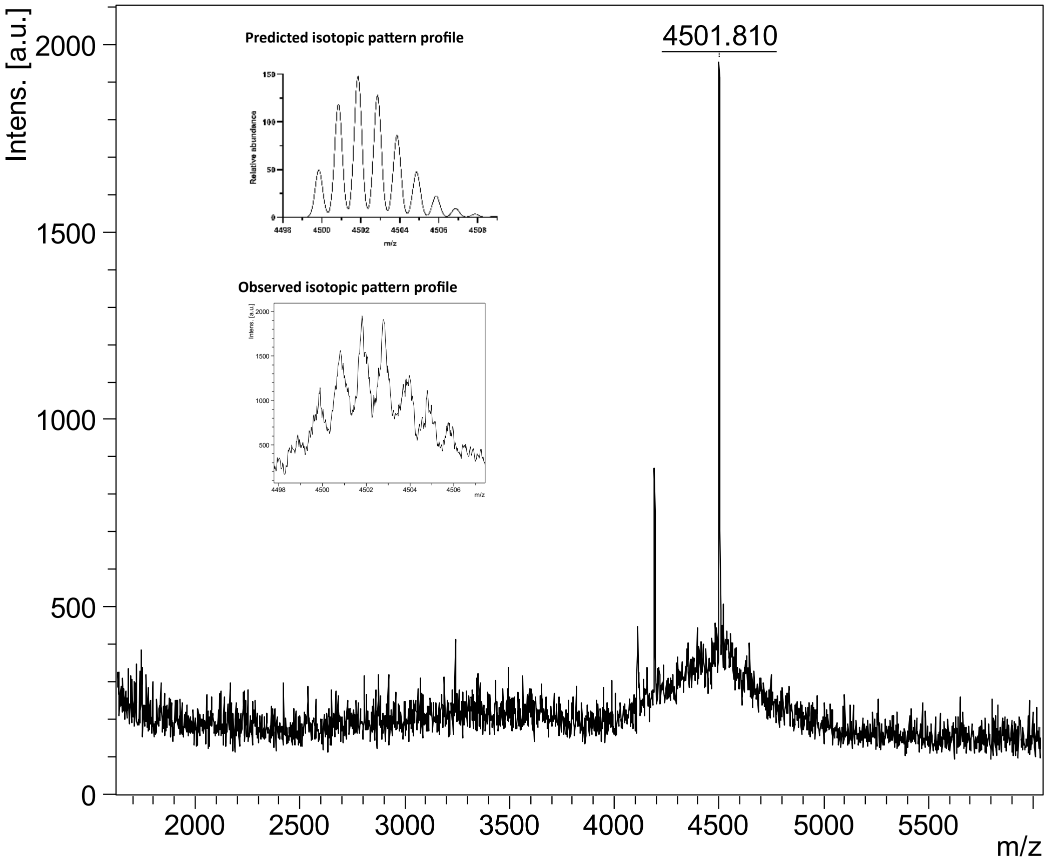

PNA1-G1-D: C-Ter: Lys(ACT GG^{G}G ATT CAC -Ac)-Dabcyl

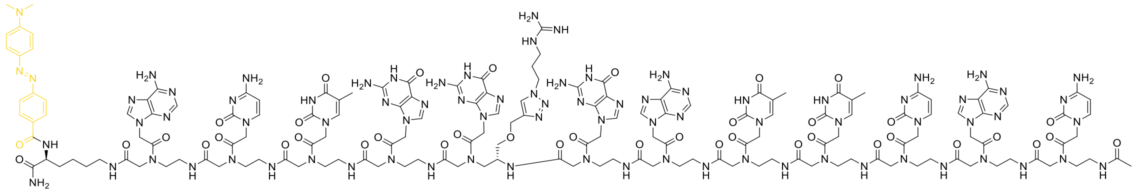

Chemical Formula: $C_{160}H_{203}N_{81}O_{40}$ **Exact Mass:** 3898.634 **Molecular Weight:** 3900.911

LC-MS-ESI+: *m/z* expected for $[M+6H]^{6+}$: 651.1, *m/z* found: 651.2; *m/z* expected for $[M+5H]^{5+}$: 781.1, *m/z* found: 781.1; *m/z* expected for $[M+4H]^{4+}$: 976.2, *m/z* found: 976.2; *m/z* expected for $[M+3H]^{3+}$: 1031.2, *m/z* found: 1031.0.

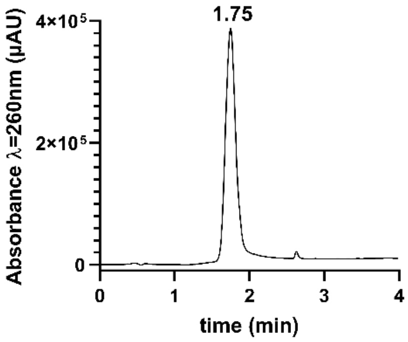

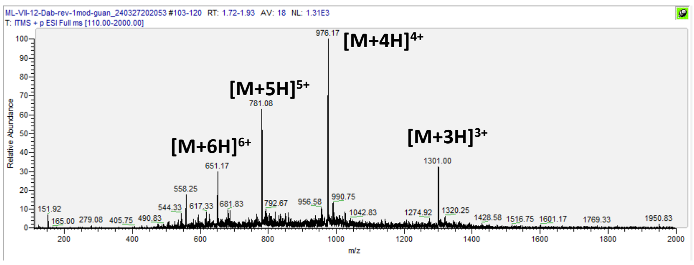

MALDI-TOF-MS: *m/z* expected for $[M+H]^{+}$: 3901.648, *m/z* found: 3901.030. *In all PNAs containing Dabcyl quencher have a secondary peak with a -132 m/z corresponding to a fragmentation of the quencher.*

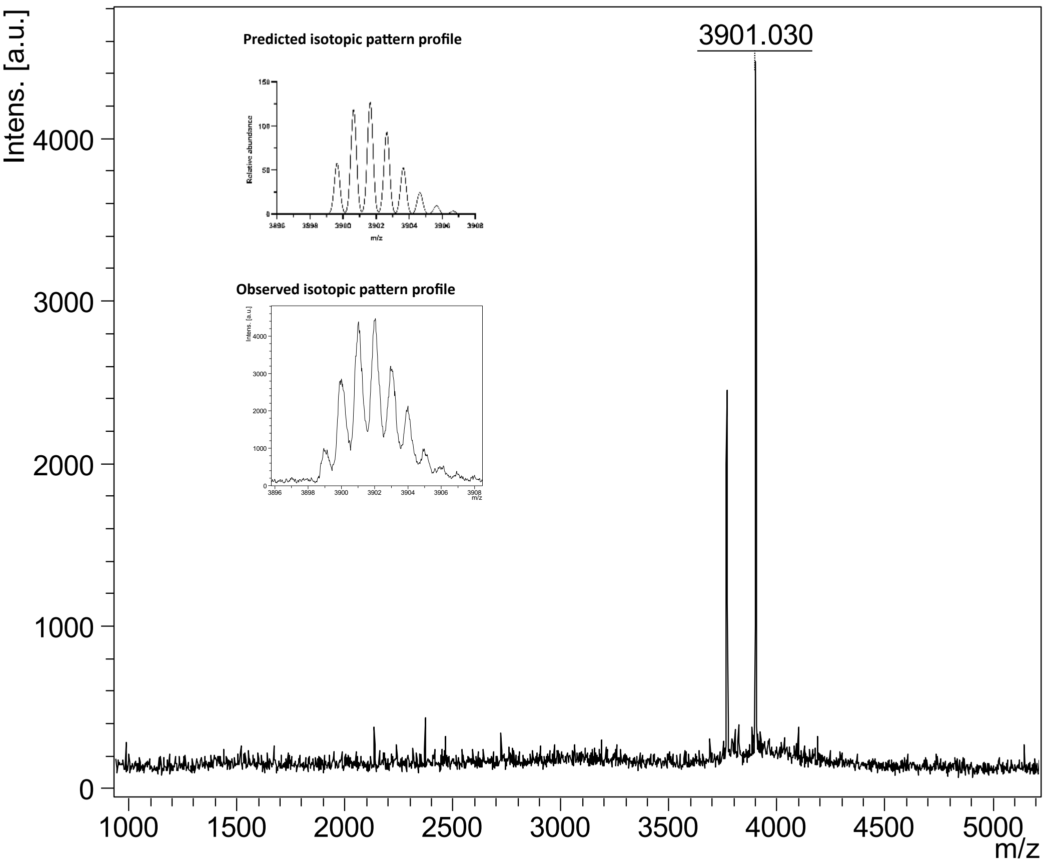

PNA1-G1-F: C-Ter: GTG AAT CC^{G}C AGT-Lys(FITC)-Ac

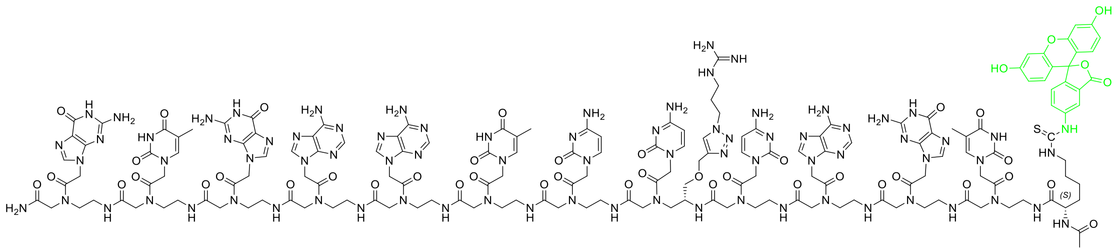

Chemical Formula: $C_{166}H_{201}N_{79}O_{44}S$ **Exact Mass:** 4036.564 **Molecular Weight:** 4039.003

LC-MS-ESI+: *m/z* expected for $[M+5H]^{5+}$: 808.7, *m/z* found: 808.8; *m/z* expected for $[M+4H]^{4+}$: 1010.6, *m/z* found: 1010.7; *m/z* expected for $[M+3H]^{3+}$: 1347.0, *m/z* found: 1347.0.

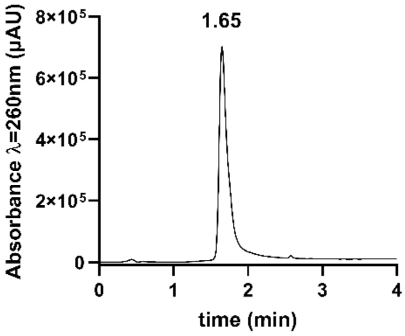

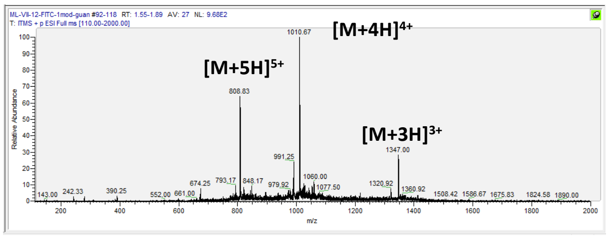

MALDI-TOF-MS: *m/z* expected for $[M+H]^{+}$: 4039.563, *m/z* found: 4039.329.

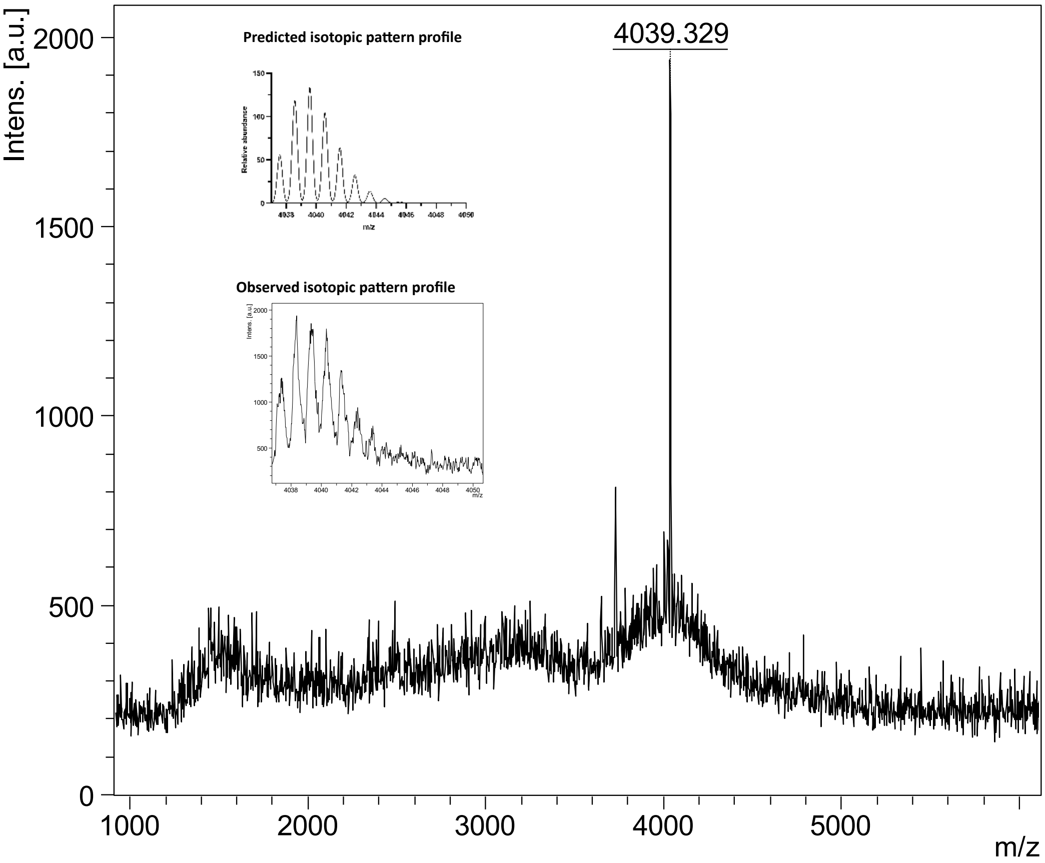

PNA1-G4-D: C-Ter: Lys(AC^{G}T GG^{G}G AT^{G}T CA^{G}C -Ac)-Dabcyl

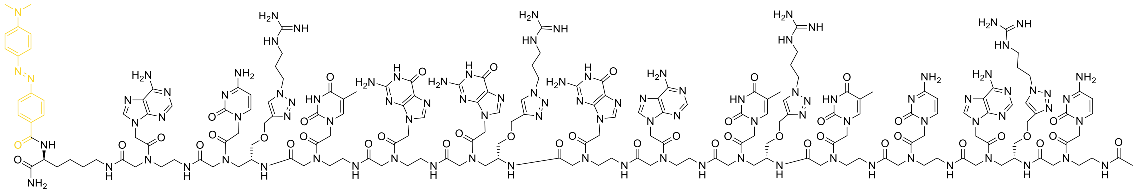

Chemical Formula: $C_{184}H_{245}N_{99}O_{43}$ **Exact Mass:** 4529.003 **Molecular Weight:** 4531.634

LC-MS-ESI+: *m/z* expected for $[M+5H]^{5+}$: 907.2, *m/z* found: 907.3; *m/z* expected for $[M+4H]^{4+}$: 1133.8, *m/z* found: 1133.9; *m/z* expected for $[M+3H]^{3+}$: 1511.3, *m/z* found: 1511.2. (*0.1% aqueous TFA solution and 0.1% TFA in HPLC grade acetonitrile used as eluents.*)

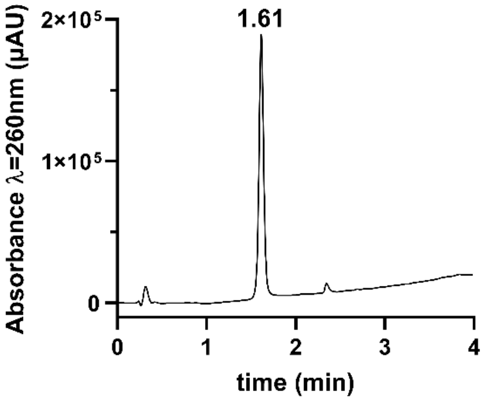

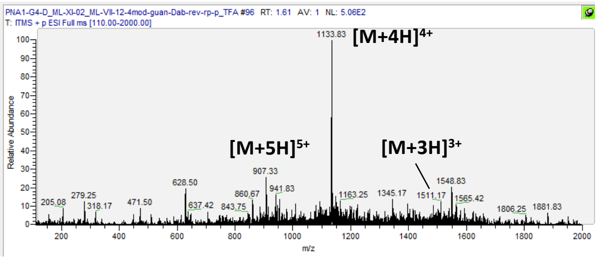

MALDI-TOF-MS: *m/z* expected for $[M+H]^{+}$: 4532.004, *m/z* found: 4532.629. *In all PNAs containing Dabcyl quencher have a secondary peak with a -132 m/z corresponding to a fragmentation of the quencher.*

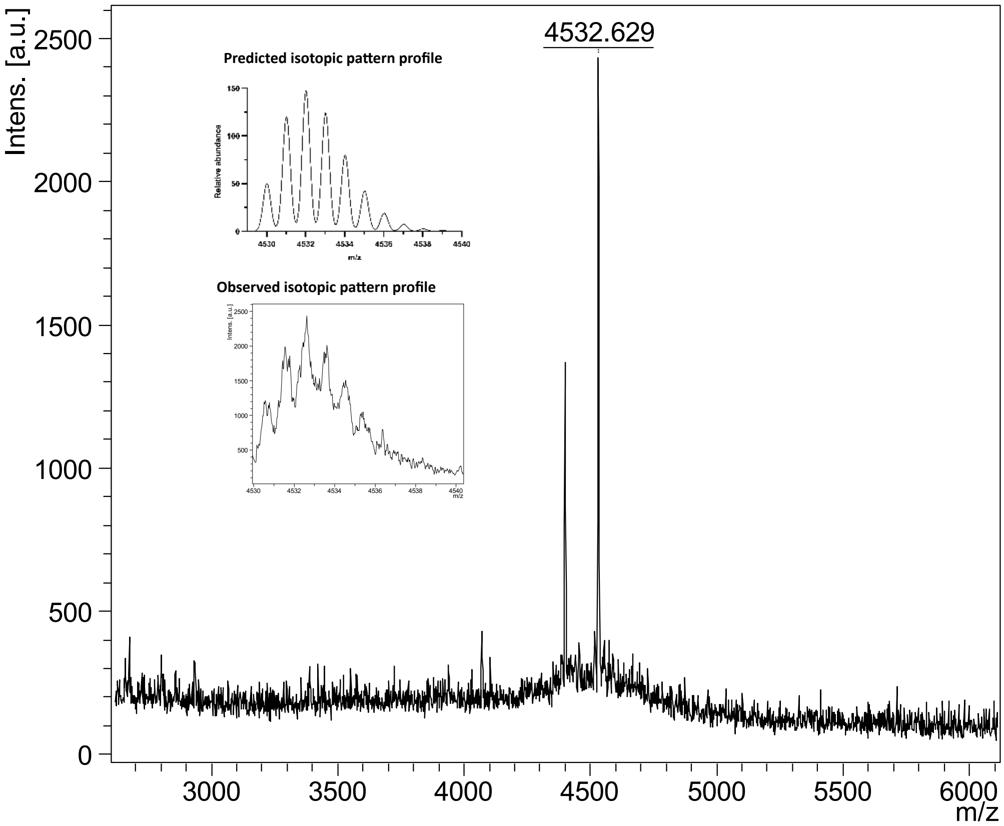

PNA1-G4-F: C-Ter: GT^{G}G AA^{G}T CC^{G}C AG^{G}T-Lys(FITC)-Ac

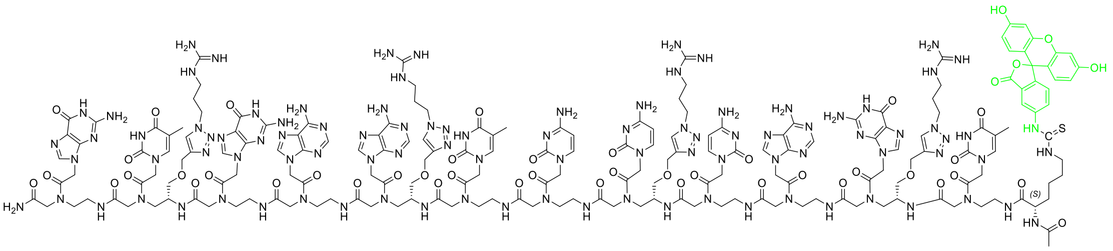

Chemical Formula: $C_{190}H_{243}N_{97}O_{47}S$ **Exact Mass:** 4666.933 **Molecular Weight:** 4669.726

LC-MS-ESI+: *m/z* expected for $[M+6H]^{6+}$: 779.2, *m/z* found: 779.3; *m/z* expected for $[M+5H]^{5+}$: 934.8, *m/z* found: 935.2; *m/z* expected for $[M+4H]^{4+}$: 1168.2, *m/z* found: 1168.5; *m/z* expected for $[M+3H]^{3+}$: 1557.3, *m/z* found: 1557.3. (*0.1% aqueous TFA solution and 0.1% TFA in HPLC grade acetonitrile used as eluents.*)

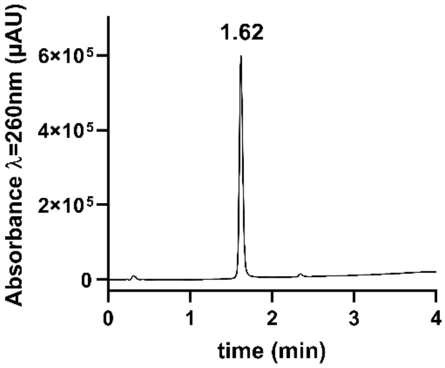

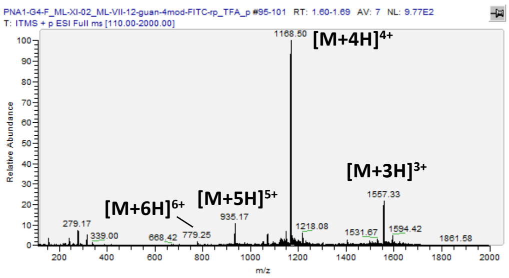

MALDI-TOF-MS: *m/z* expected for [M+H]$^{+}$: 4669.931, *m/z* found: 4669.190.

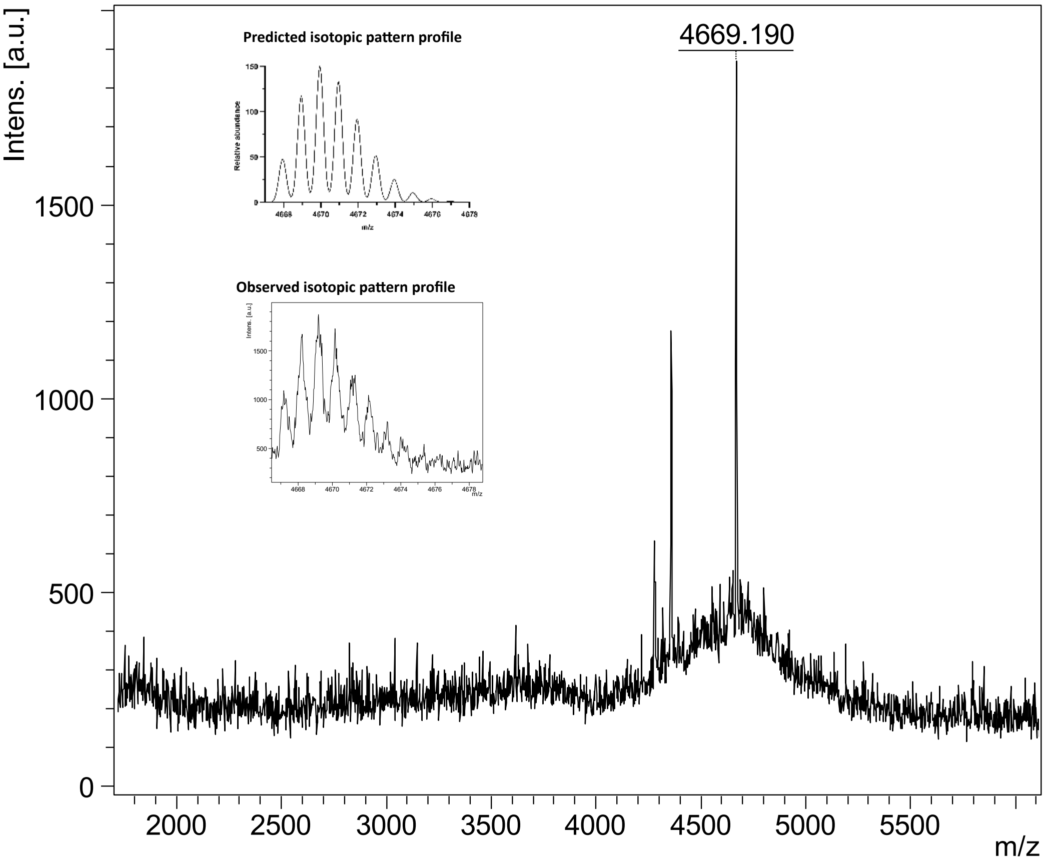

PNA1-P1-D: C-Ter: Lys(ACT GG^{P}G ATT CAC -Ac)-Dabcyl

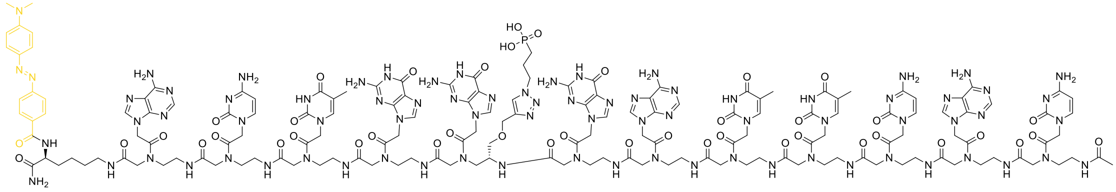

Chemical Formula: $C_{159}H_{201}N_{78}O_{43}P$ **Exact Mass:** 3921.568 **Molecular Weight:** 3923.834

LC-MS-ESI+: *m/z* expected for $[M+6H]^{6+}$: 654.9, *m/z* found: 655.3; *m/z* expected for $[M+5H]^{5+}$: 785.7, *m/z* found: 786.1; *m/z* expected for $[M+4H]^{4+}$: 981.9, *m/z* found: 982.2; *m/z* expected for $[M+3H]^{3+}$: 1308.8, *m/z* found: 1308.8.

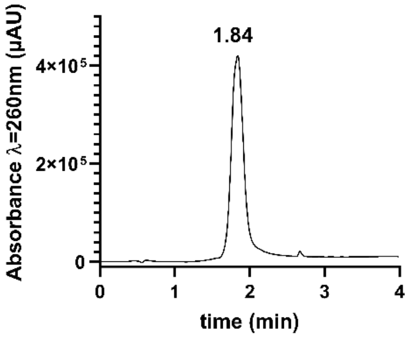

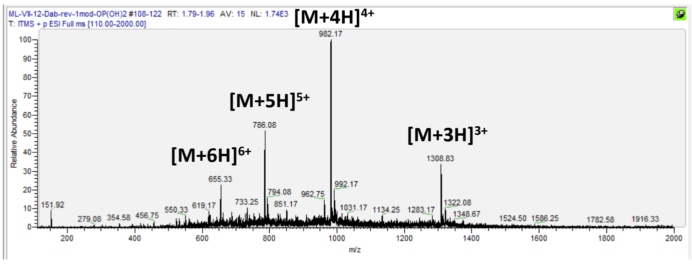

MALDI-TOF-MS: *m/z* expected for $[M+H]^+$: 3924.575, *m/z* found: 3924.998. *In all PNAs containing Dabcyl quencher have a secondary peak with a -132 m/z corresponding to a fragmentation of the quencher.*

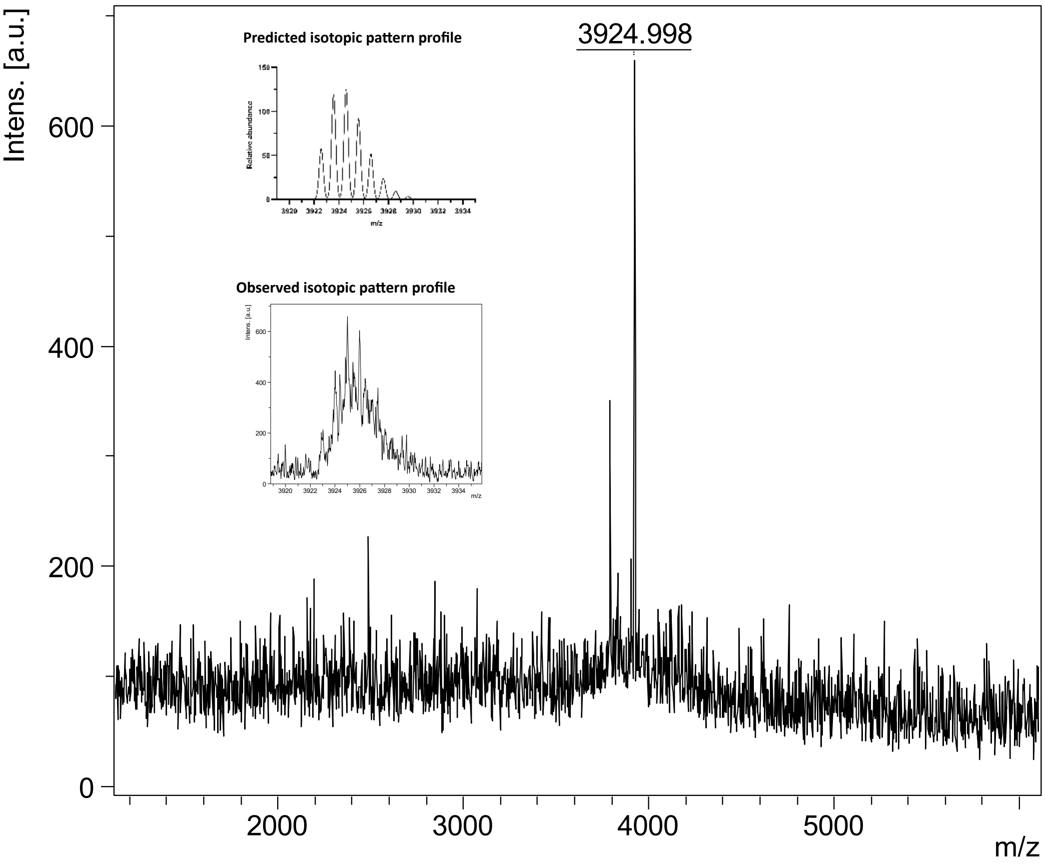

PNA1-P4-D: C-Ter: Lys(AC^{P}T GG^{P}G AT^{P}T CA^{P}C -Ac)-Dabcyl

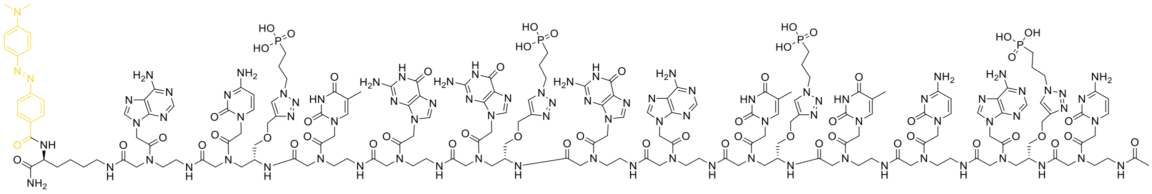

Chemical Formula: $C_{180}H_{237}N_{87}O_{55}P_4$ **Exact Mass:** 4620.737 **Molecular Weight:** 4623.325

LC-MS-ESI+: *m/z* expected for $[M+5H]^{5+}$: 925.6, *m/z* found: 926.1; *m/z* expected for $[M+4H]^{4+}$: 1156.9, *m/z* found: 1157.0; *m/z* expected for $[M+3H]^{3+}$: 1541.9, *m/z* found: 1542.3.

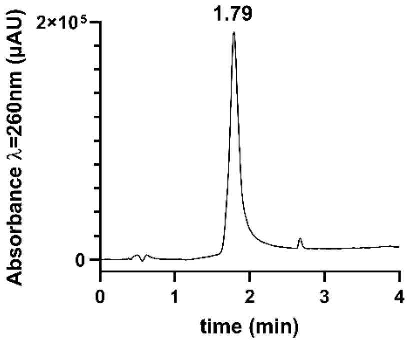

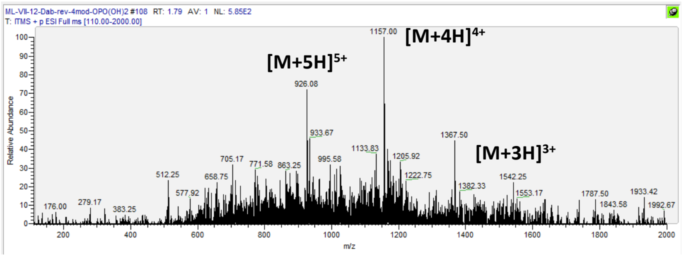

MALDI-TOF-MS: *m/z* expected for $[M+H]^{+}$: 4624.752, *m/z* found: 4623.978. *In all PNAs containing Dabcyl quencher have a secondary peak with a -132 m/z corresponding to a fragmentation of the quencher.*

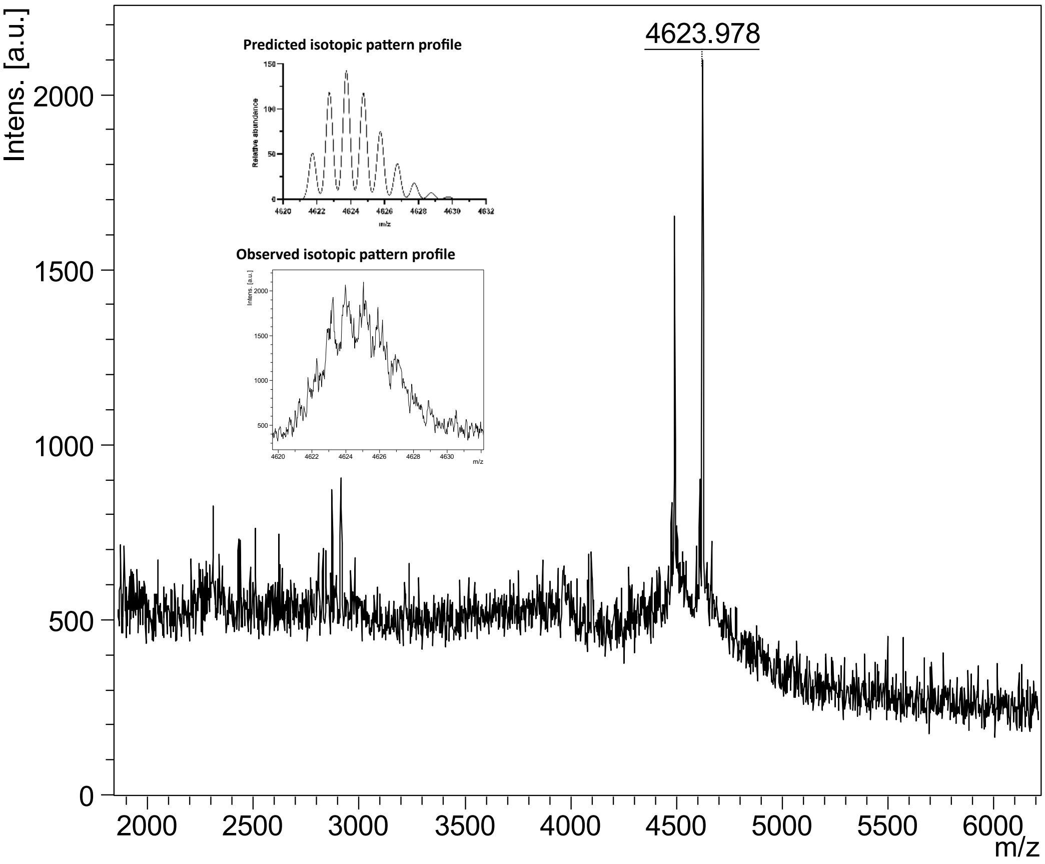

PNA1-P1-F: C-Ter: GTG AAT CC^{P}C AGT-Lys(FITC)-Ac

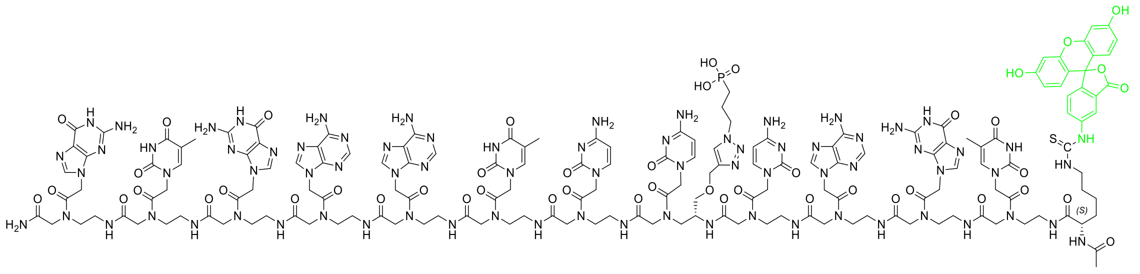

Chemical Formula: $C_{165}H_{199}N_{76}O_{47}PS$ **Exact Mass:** 4059.498 **Molecular Weight:** 4061.926

LC-MS-ESI+: *m/z* expected for $[M+4H]^{4+}$: 1016.4, *m/z* found: 1016.8; *m/z* expected for $[M+3H]^{3+}$: 1354.8, *m/z* found: 1354.8. (*0.1% aqueous TFA solution and 0.1% TFA in HPLC grade acetonitrile used as eluents.*)

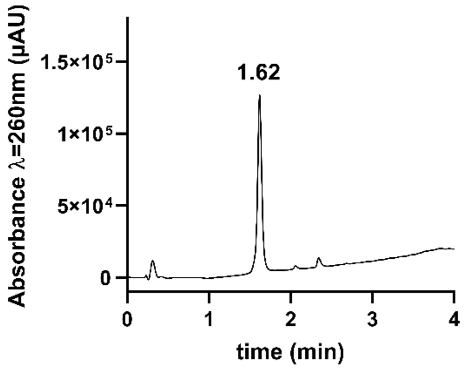

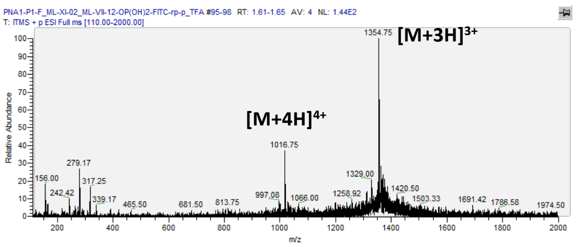

MALDI-TOF-MS: *m/z* expected for $[M+H]^+$: 4062.514, *m/z* found: 4061.662.

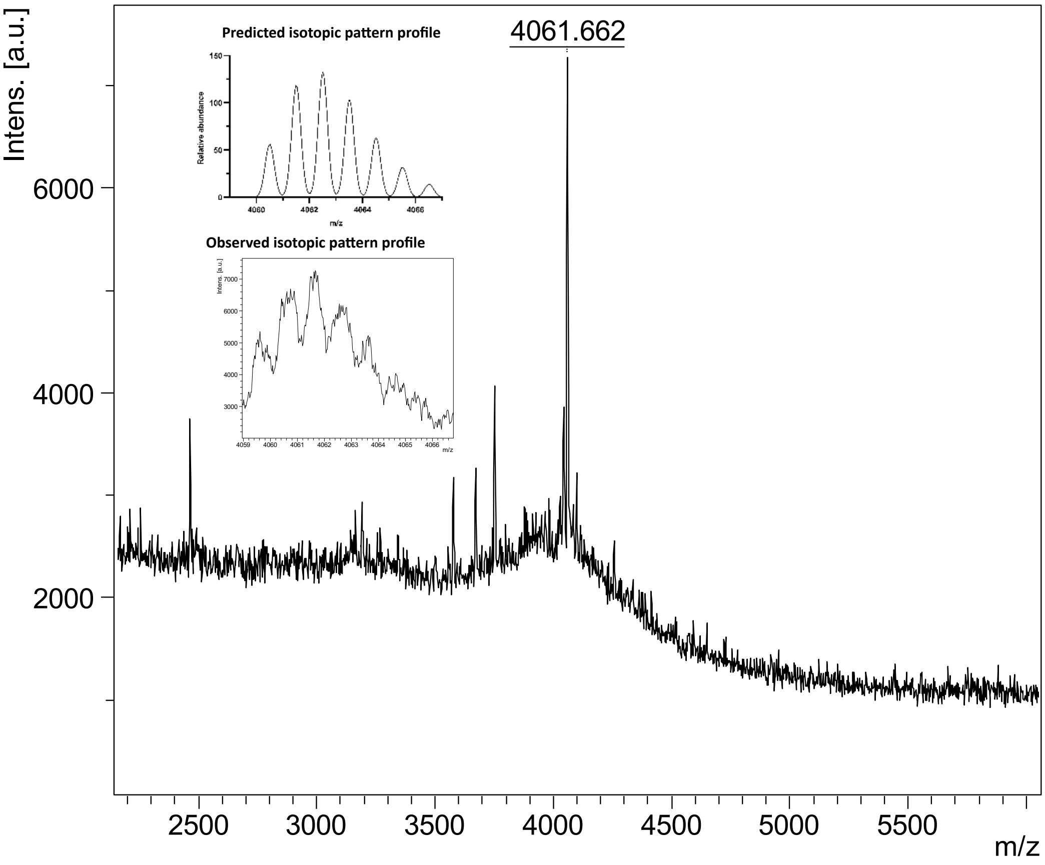

PNA1-P4-F: C-Ter: GT^{P}G AA^{P}T CC^{P}C AG^{P}T-Lys(FITC)-Ac

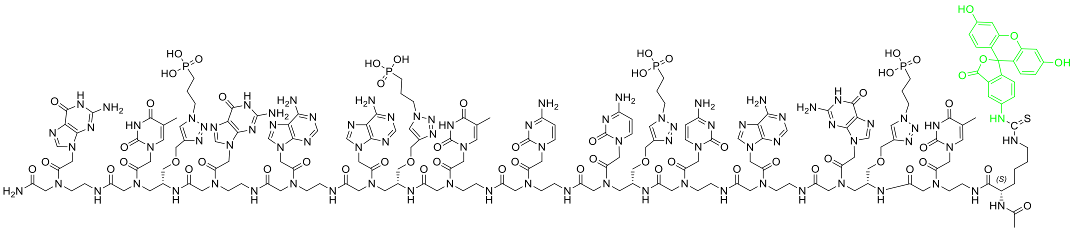

Chemical Formula: $C_{186}H_{235}N_{85}O_{59}P_4S$ **Exact Mass:** 4758.667 **Molecular Weight:** 4761.417

LC-MS-ESI+: *m/z* expected for $[M+5H]^{5+}$: 953.1, *m/z* found: 953.3; *m/z* expected for $[M+4H]^{4+}$: 1191.2, *m/z* found: 1191.3; *m/z* expected for $[M+3H]^{3+}$: 1587.9, *m/z* found: 1587.7.

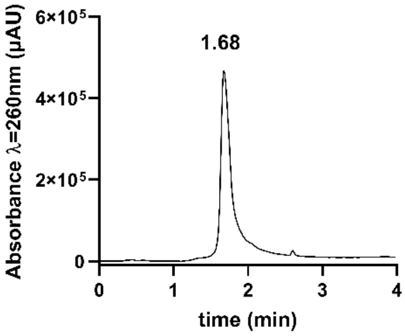

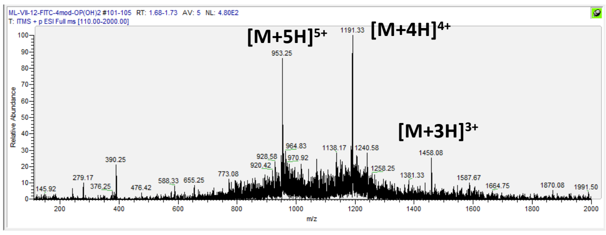

MALDI-TOF-MS: *m/z* expected for $[M+H]^{+}$: 4761.677, *m/z* found: 4761.874.

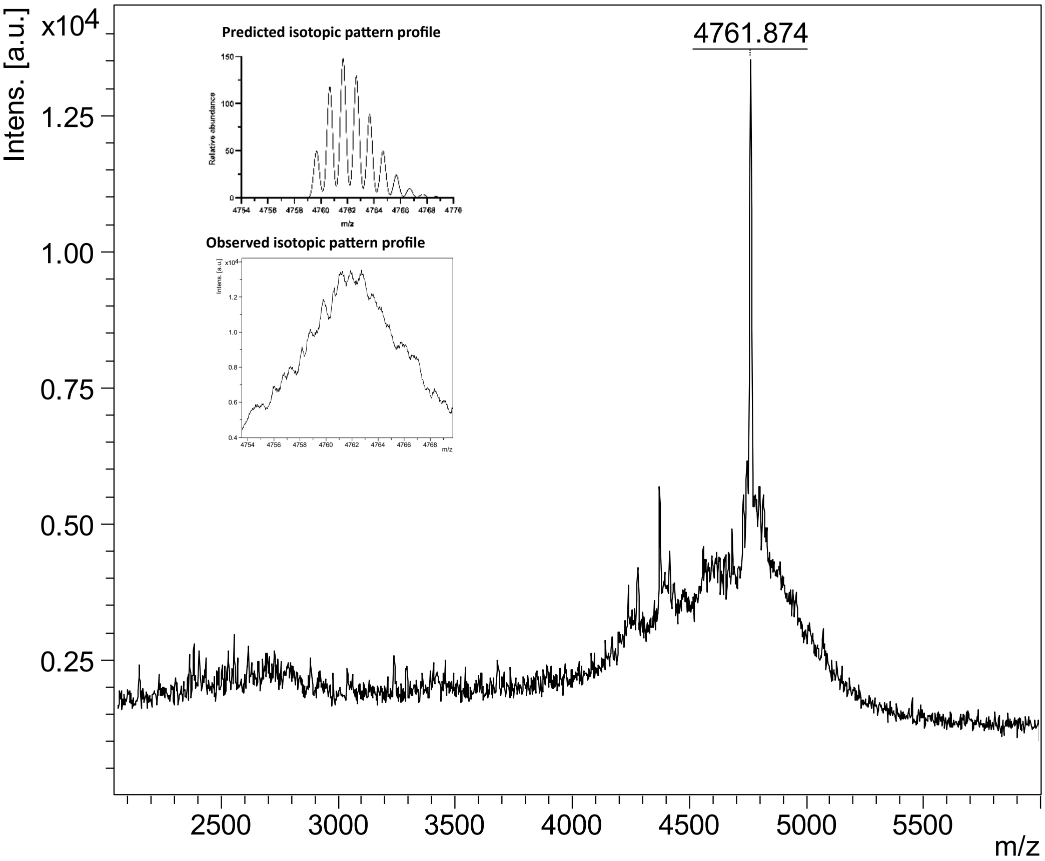

PNA3-Bt: C-Ter: GG ATT CAC-Lys(Bt)-Ac

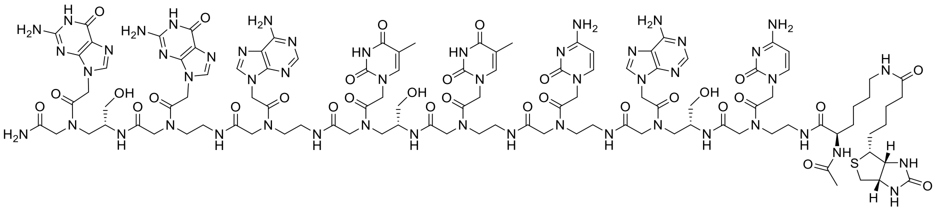

Chemical Formula: $C_{107}H_{143}N_{51}O_{31}S$ **Exact Mass:** 2670.090 **Molecular Weight:** 2671.707

LC-MS-ESI+: *m/z* expected for $[M+4H]^{4+}$: 668.8, *m/z* found: 669.0; *m/z* expected for $[M+3H]^{3+}$: 891.1, *m/z* found: 891.6; *m/z* expected for $[M+2H]^{2+}$: 1336.6, *m/z* found: 1336.6.

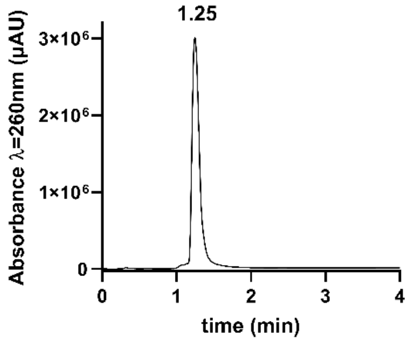

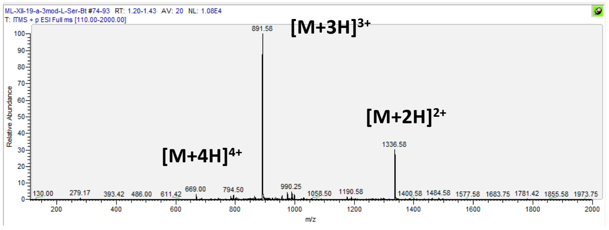

MALDI-TOF-MS: *m/z* expected for $[M+H]^+$: 2672.104, *m/z* found: 2672.439.

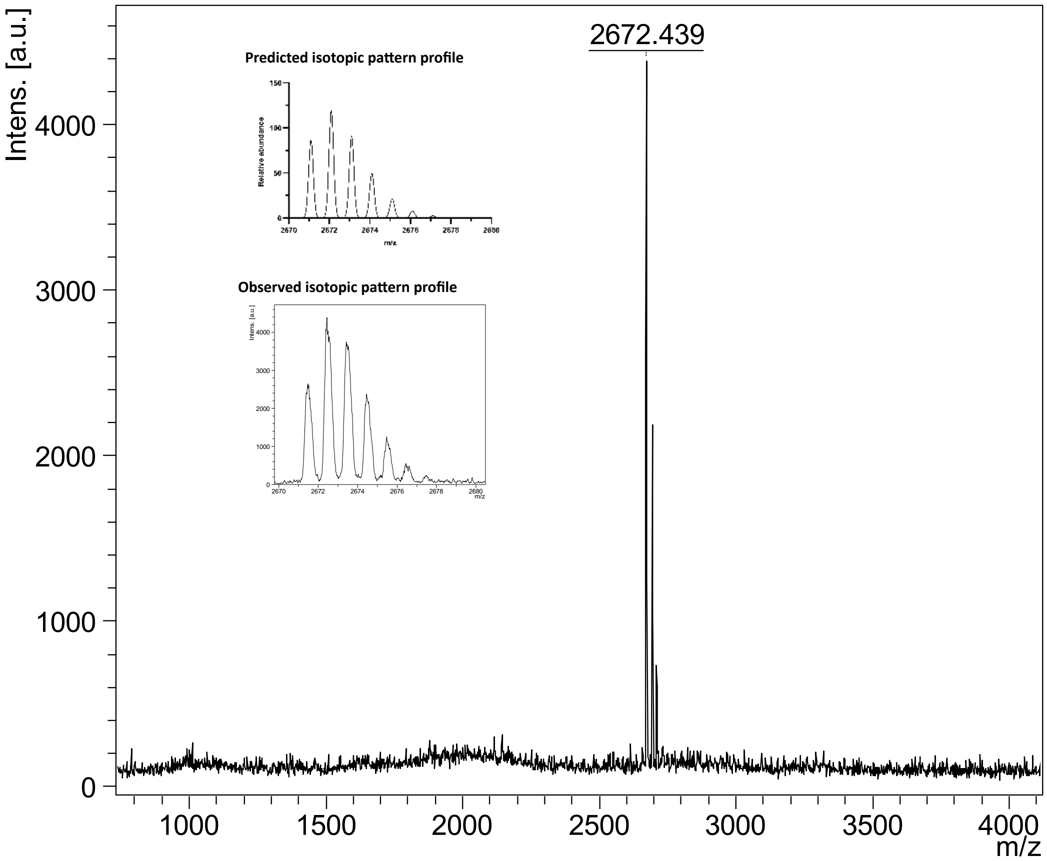

PNA3-AAmm-Bt: C-Ter: GG AAT CAC-Lys(Bt)-Ac

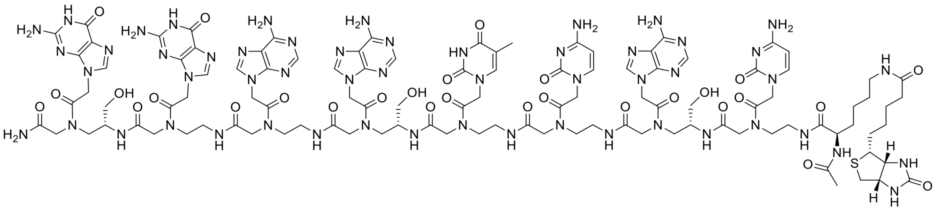

Chemical Formula: $C_{107}H_{142}N_{54}O_{29}S$ **Exact Mass:** 2679.102 **Molecular Weight:** 2680.722

LC-MS-ESI+: *m/z* expected for $[M+4H]^{4+}$: 671.0, *m/z* found: 671.2; *m/z* expected for $[M+3H]^{3+}$: 894.4, *m/z* found: 894.6; *m/z* expected for $[M+2H]^{2+}$: 1341.1, *m/z* found: 1341.2.

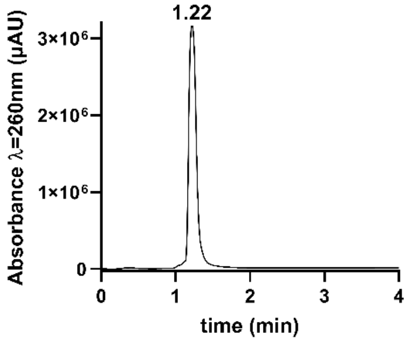

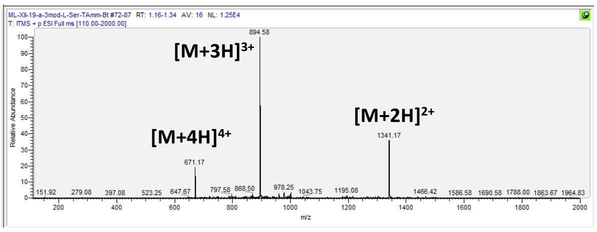

MALDI-TOF-MS: *m/z* expected for $[M+H]^+$: 2681.121, *m/z* found: 2681.776.

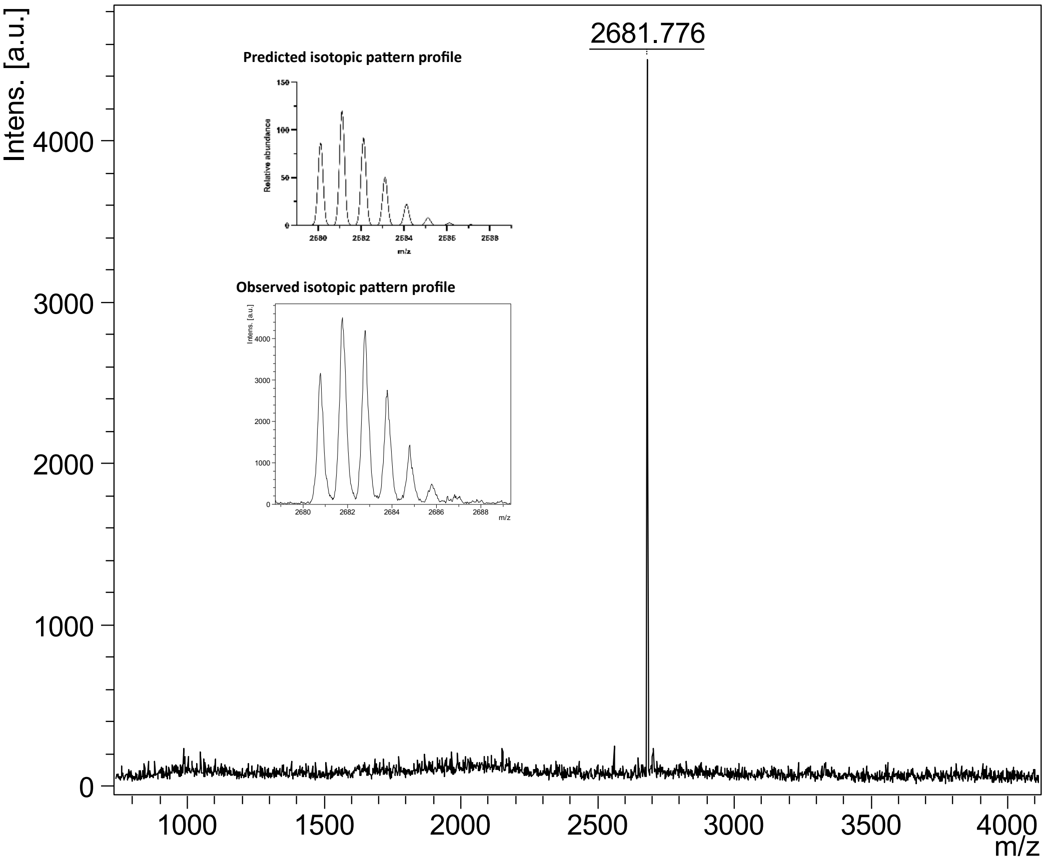

PNA2-ach: C-Ter: GTG AAT CC -Ac

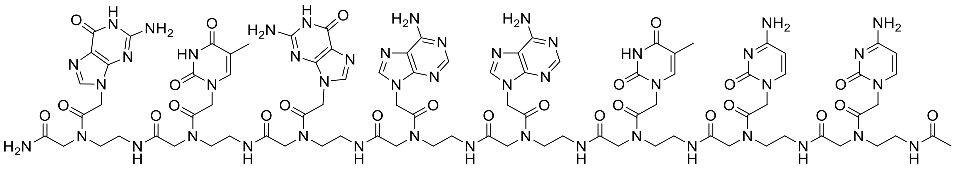

Chemical Formula: $C_{88}H_{111}N_{47}O_{25}$ **Exact Mass:** 2225.886 **Molecular Weight:** 2227.160

LC-MS-ESI+: *m/z* expected for $[M+4H]^{4+}$: 557.7, *m/z* found: 557.8; *m/z* expected for $[M+3H]^{3+}$: 743.3, *m/z* found: 743.4; *m/z* expected for $[M+2H]^{2+}$: 1114.5, *m/z* found: 1114.5.

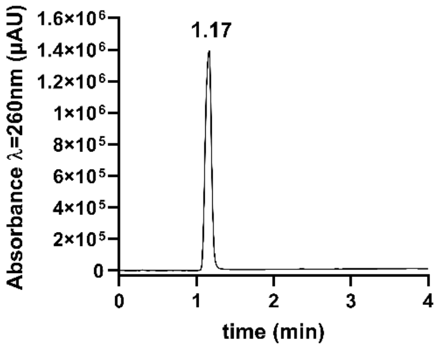

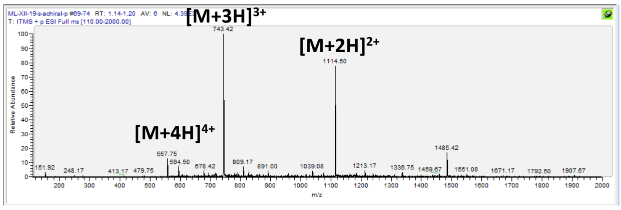

MALDI-TOF-MS: *m/z* expected for $[M+Na]^+$: 2227.901, *m/z* found: 2227.556.

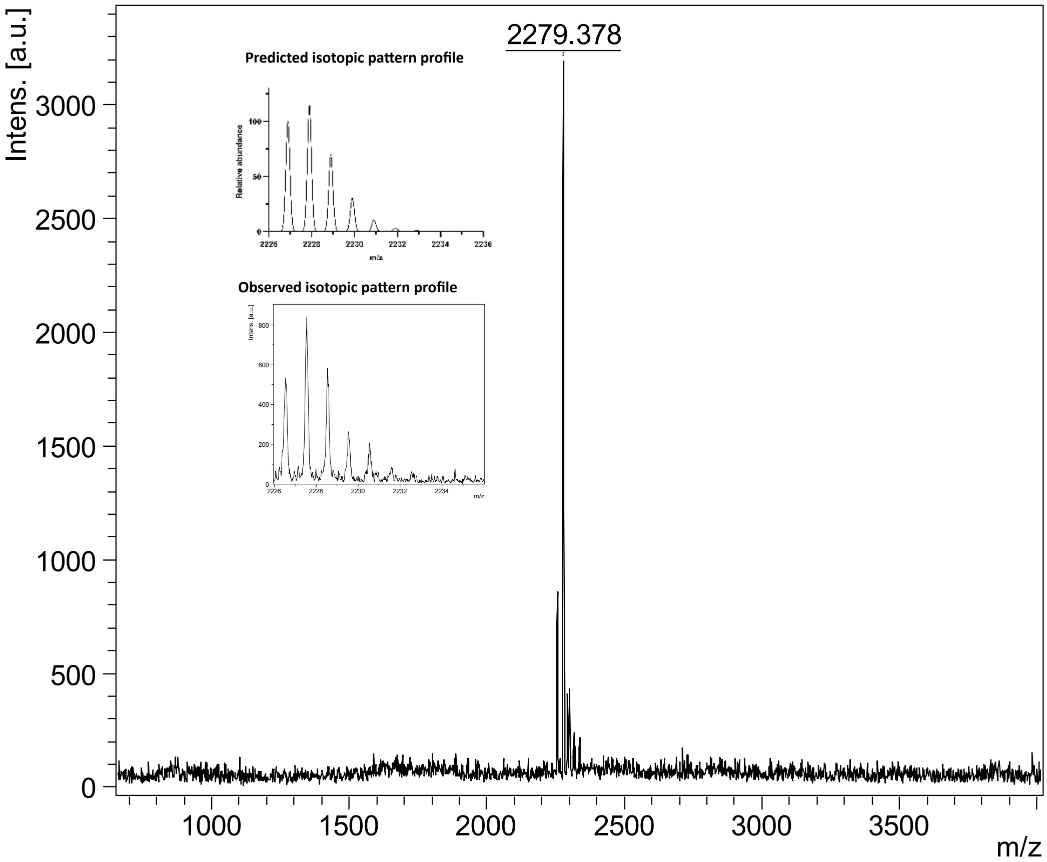

PNA2-H1: C-Ter: GTG AA^{H}T CC -Ac

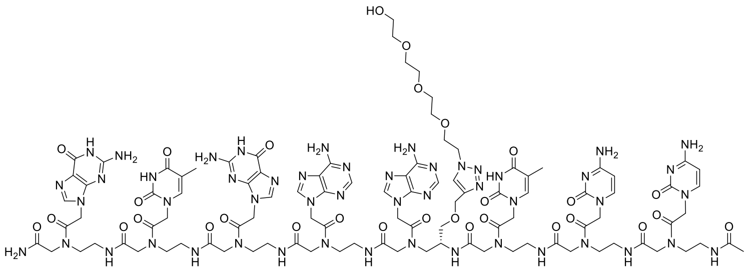

Chemical Formula: $C_{100}H_{132}N_{50}O_{30}$ **Exact Mass:** 2513.034 **Molecular Weight:** 2514.476

LC-MS-ESI+: *m/z* expected for $[M+4H]^{4+}$: 629.5, *m/z* found: 629.7; *m/z* expected for $[M+3H]^{3+}$: 839.0, *m/z* found: 839.0; *m/z* expected for $[M+2H]^{2+}$: 1258.0, *m/z* found: 1257.8.

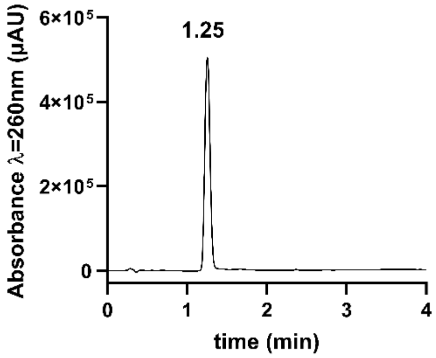

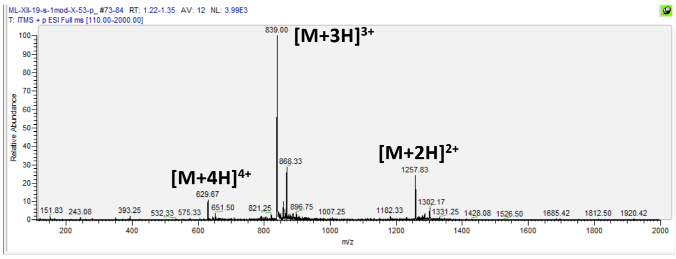

MALDI-TOF-MS: *m/z* expected for $[M+Na]^{+}$: 2537.030, *m/z* found: 2536.576.

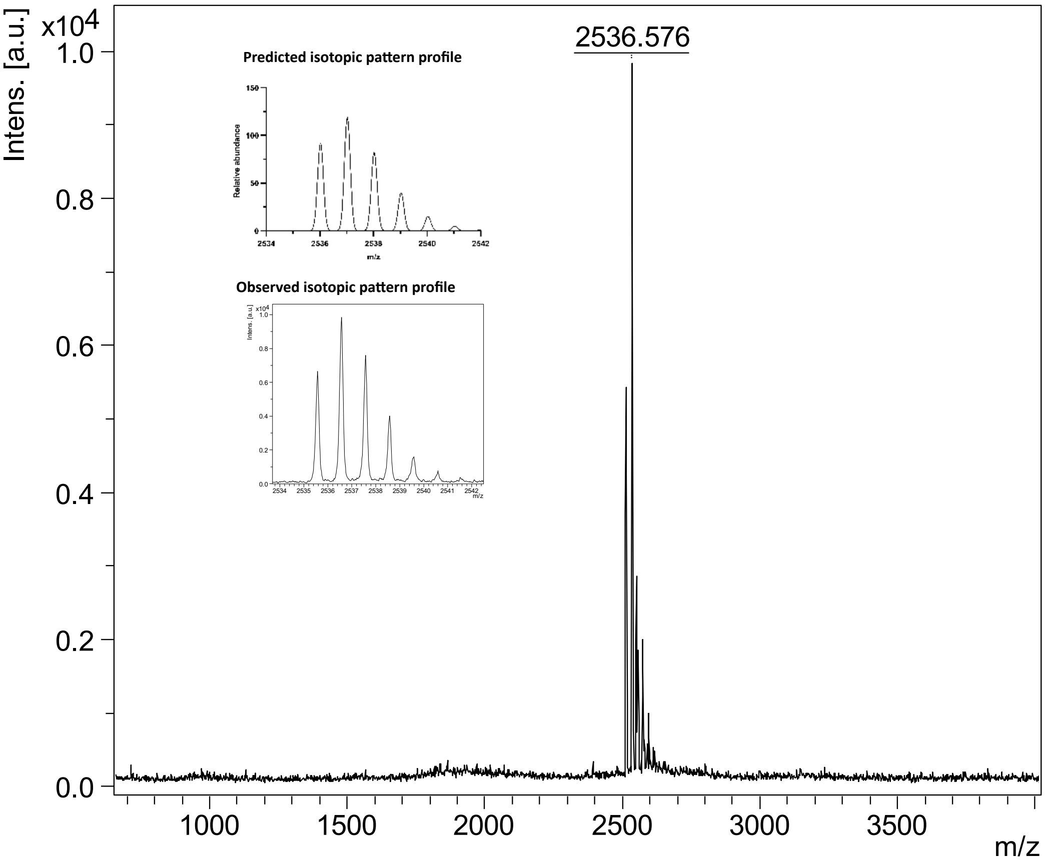

PNA2-H3: C-Ter: GT^{H}G AA^{H}T CC^{H} -Ac

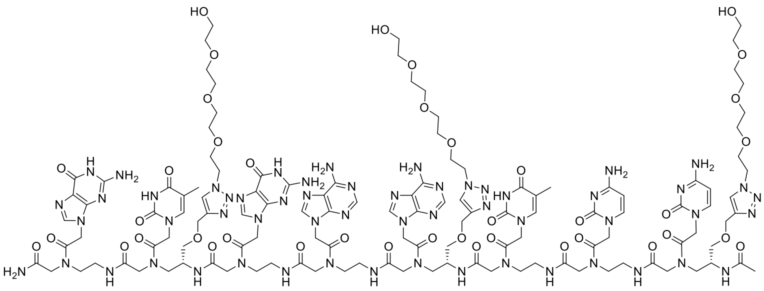

Chemical Formula: $C_{124}H_{174}N_{56}O_{40}$ **Exact Mass:** 3087.330 **Molecular Weight:** 3089.108

LC-MS-ESI+: *m/z* expected for $[M+4H]^{4+}$: 773.3, *m/z* found: 773.2; *m/z* expected for $[M+3H]^{3+}$: 1030.4, *m/z* found: 1030.4; *m/z* expected for $[M+2H]^{2+}$: 1545.2, *m/z* found: 1544.8.

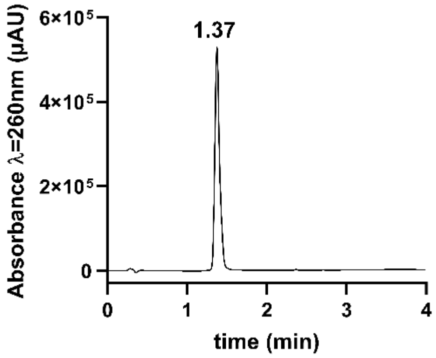

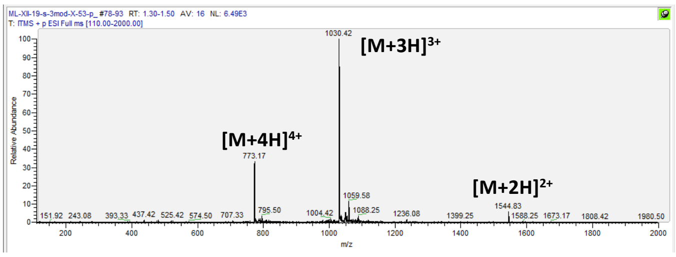

MALDI-TOF-MS: *m/z* expected for $[M+Na]^{+}$: 3111.313, *m/z* found: 3110.563.

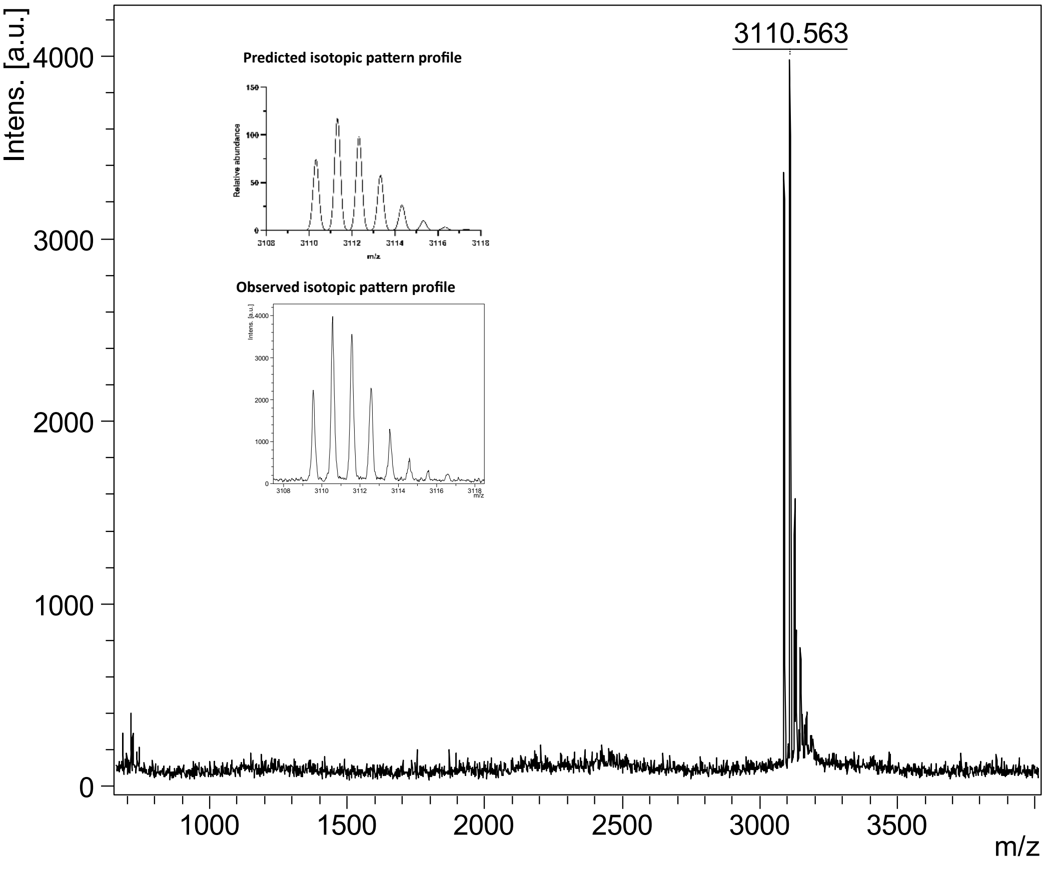

PNA2-A3: C-Ter: GT^{A}G AA^{A}T CC^{A} -Ac

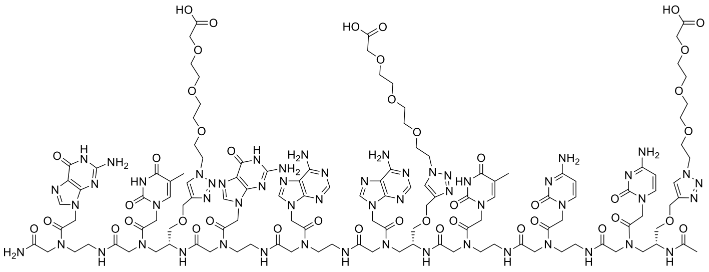

Chemical Formula: $C_{124}H_{168}N_{56}O_{43}$ **Exact Mass:** 3129.268 **Molecular Weight:** 3131.057

LC-MS-ESI+: *m/z* expected for $[M+4H]^{4+}$: 783.6, *m/z* found: 783.6; *m/z* expected for $[M+3H]^{3+}$: 1044.4, *m/z* found: 1044.4; *m/z* expected for $[M+2H]^{2+}$: 1566.1, *m/z* found: 1565.8.

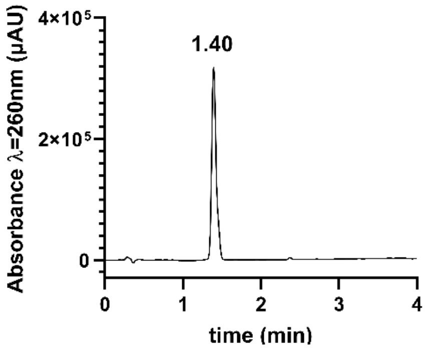

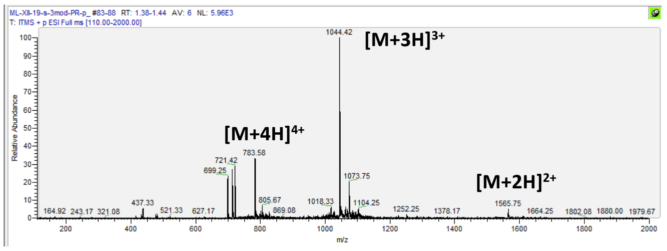

MALDI-TOF-MS: *m/z* expected for $[M+H]^{+}$: 3131.282, *m/z* found: 3130.871.

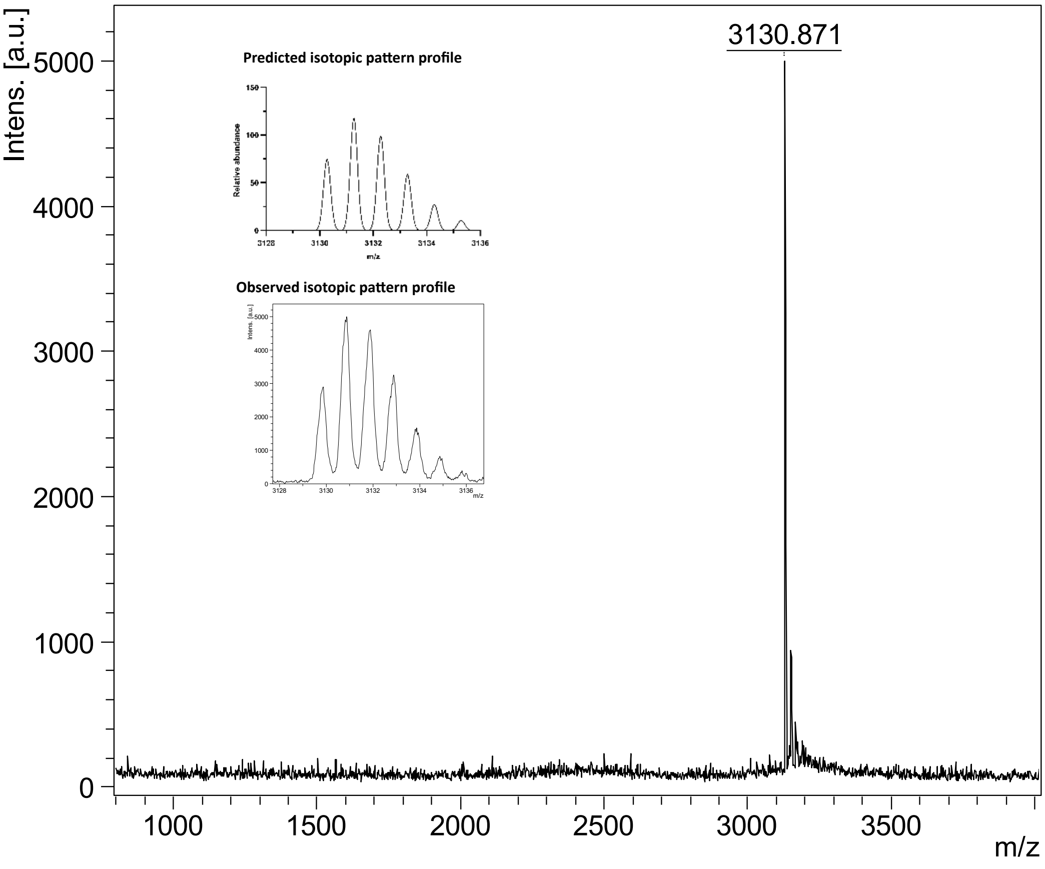

PNA2-N3: C-Ter: GT^{N}G AA^{N}T CC^{N} -Ac

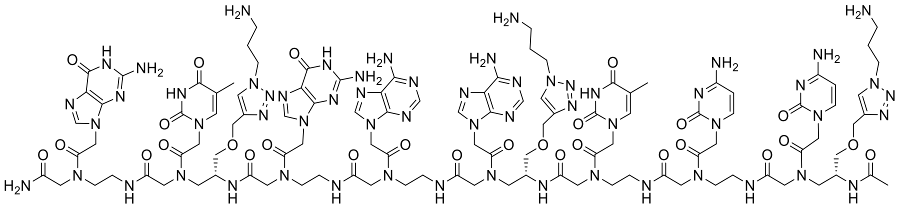

Chemical Formula: $C_{109}H_{147}N_{59}O_{28}$ **Exact Mass:** 2730.189 **Molecular Weight:** 2731.760

LC-MS-ESI+: *m/z* expected for $[M+4H]^{4+}$: 683.8, *m/z* found: 684.0; *m/z* expected for $[M+3H]^{3+}$: 911.4, *m/z* found: 911.6; *m/z* expected for $[M+2H]^{2+}$: 1366.6, *m/z* found: 1366.7 .

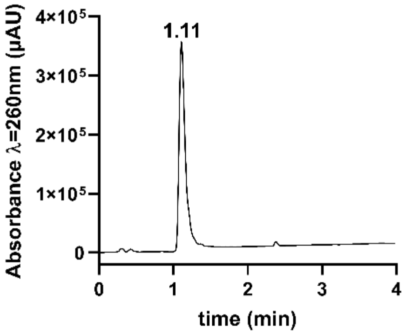

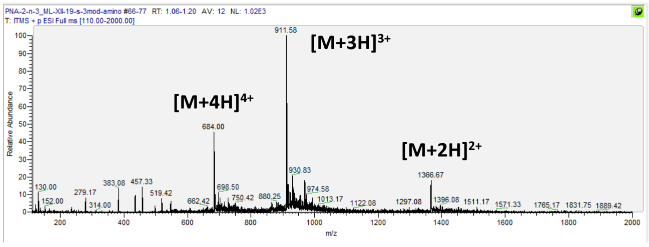

MALDI-TOF-MS: *m/z* expected for $[M+H]^{+}$: 2732.194, *m/z* found: 2732.269.

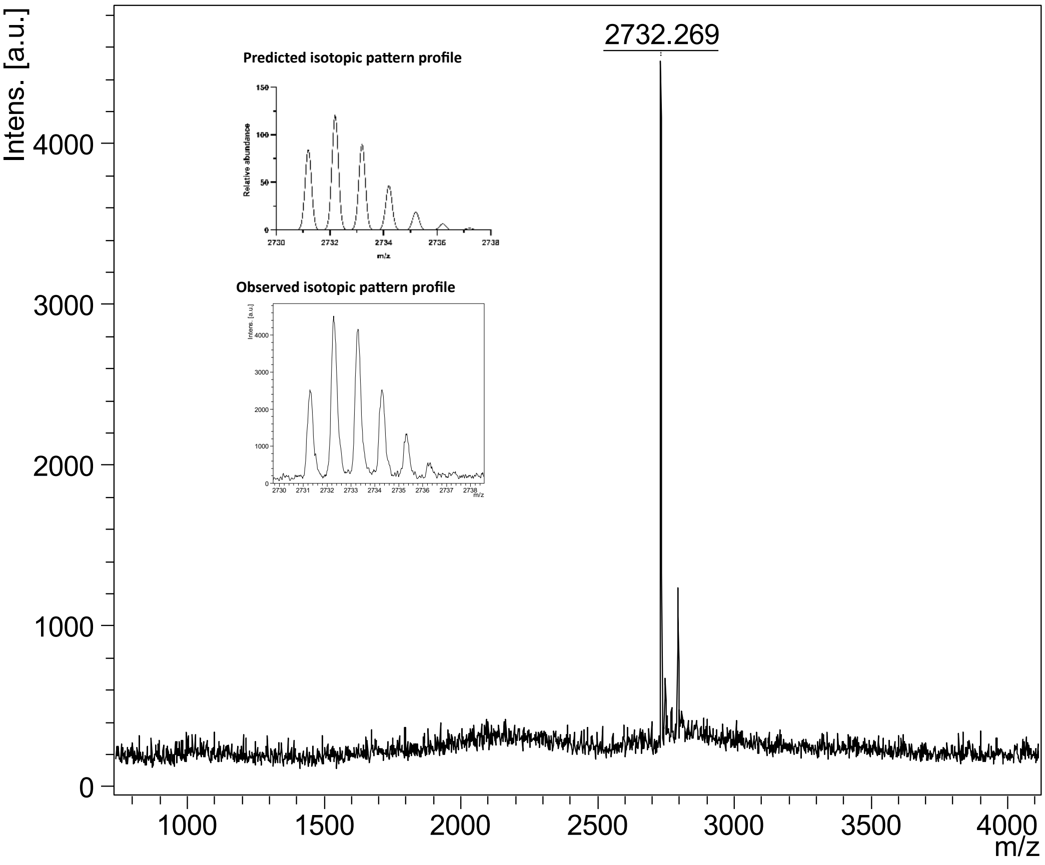

PNA2-G3: C-Ter: GT^{G}G AA^{G}T CC^{G} -Ac

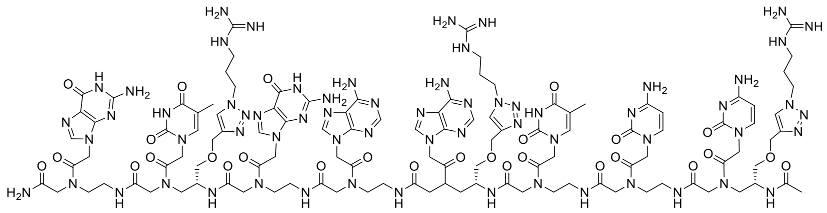

Chemical Formula: $C_{113}H_{154}N_{64}O_{28}$ **Exact Mass:** 2855.259 **Molecular Weight:** 2856.895

LC-MS-ESI+: *m/z* expected for $[M+4H]^{4+}$: 715.1, *m/z* found: 715.5; *m/z* expected for $[M+3H]^{3+}$: 953.1, *m/z* found: 953.5; *m/z* expected for $[M+2H]^{2+}$: 1429.1, *m/z* found: 1429.6.

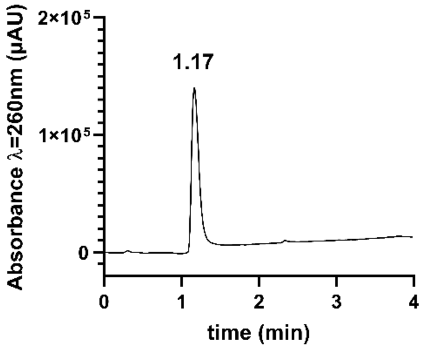

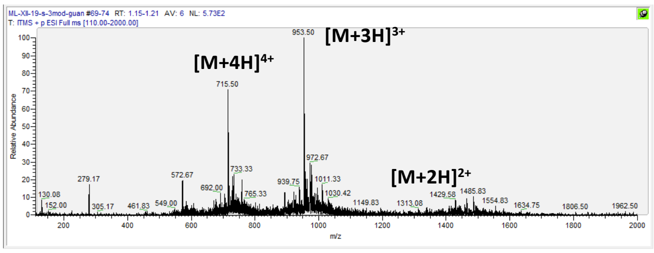

MALDI-TOF-MS: *m/z* expected for $[M+H]^{+}$: 2857.269, *m/z* found: 2857.701.

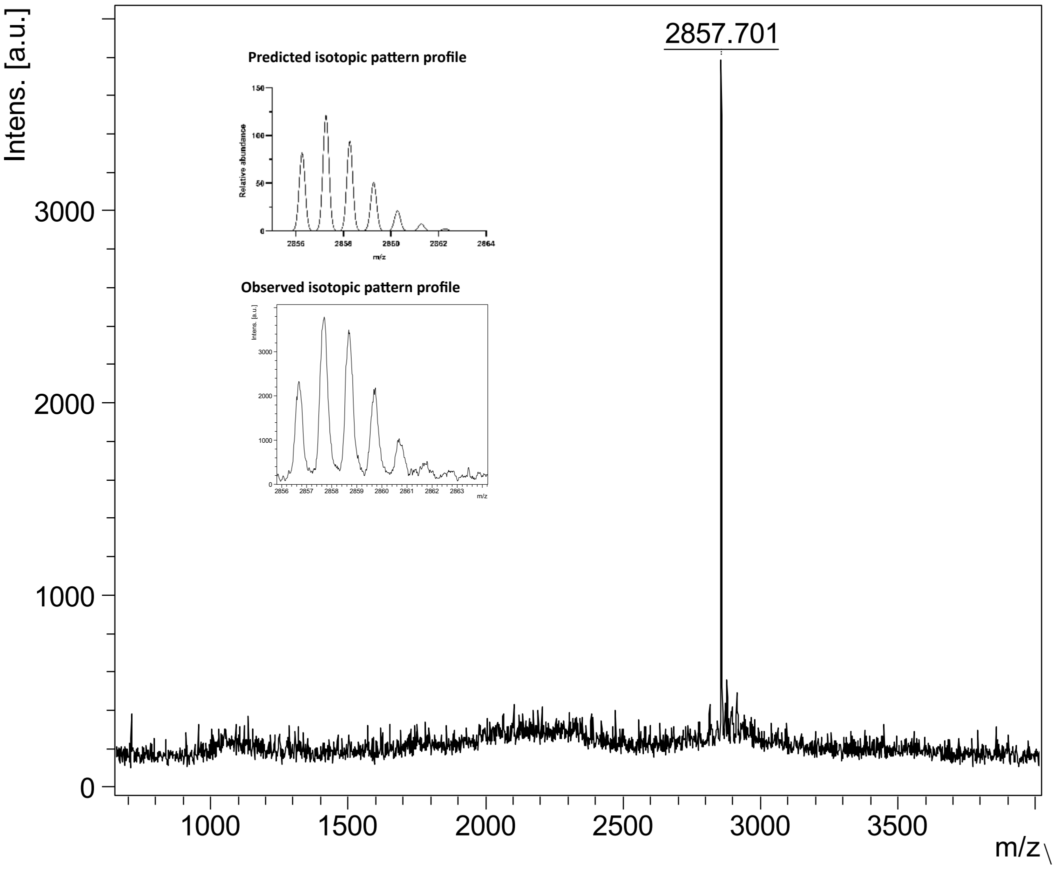

PNA2-P3: C-Ter: GT^{P}G AA^{P}T CC^P -Ac

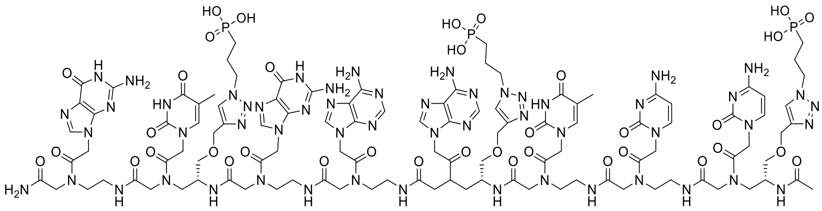

Chemical Formula: $C_{110}H_{148}N_{55}O_{37}P_3$ **Exact Mass:** 2924.060 **Molecular Weight:** 2925.663

LC-MS-ESI+: *m/z* expected for $[M+4H]^{4+}$: 732.3, *m/z* found: 732.7; *m/z* expected for $[M+3H]^{3+}$: 976.0, *m/z* found: 976.6; *m/z* expected for $[M+2H]^{2+}$: 1463.5, *m/z* found: 1464.0.

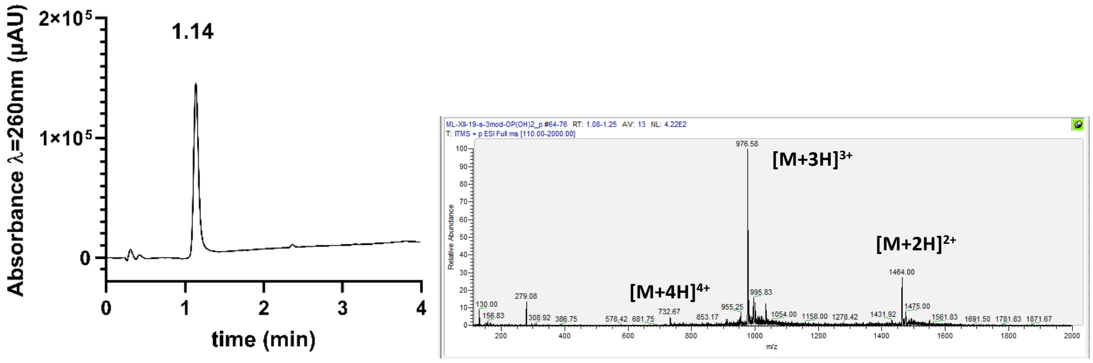

MALDI-TOF-MS: *m/z* expected for $[M+H]^+$: 2926.089, *m/z* found: 2926.953.

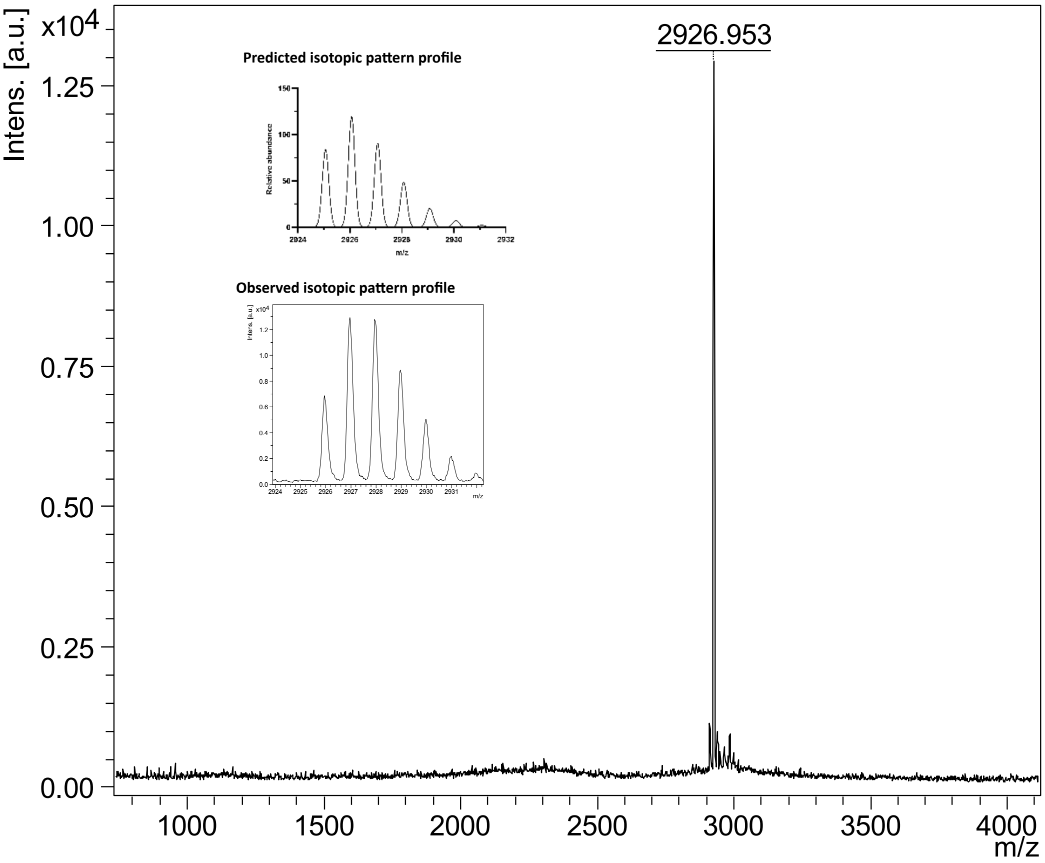

PNA2-pc-ach: C-Ter: GTG^{+} AAT C^{+}C -Ac

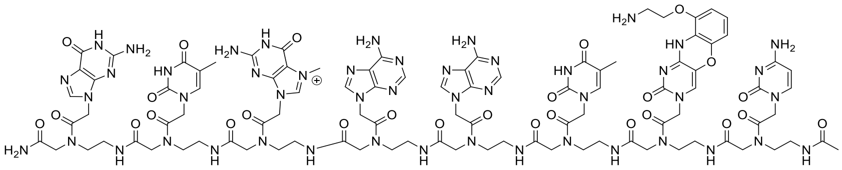

Chemical Formula: $C_{97}H_{121}N_{48}O_{27}^{+}$ **Exact Mass:** 2389.957 **Molecular Weight:** 2391.343

LC-MS-ESI+: *m/z* expected for $[M+3H]^{4+}$: 598.5, *m/z* found: 598.8; *m/z* expected for $[M+2H]^{3+}$: 797.7, *m/z* found: 797.8; *m/z* expected for $[M+H]^{2+}$: 1196.0, *m/z* found: 1196.1.

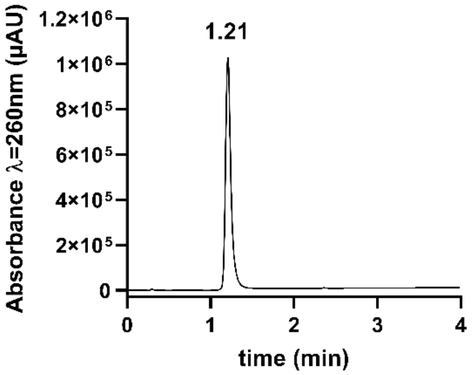

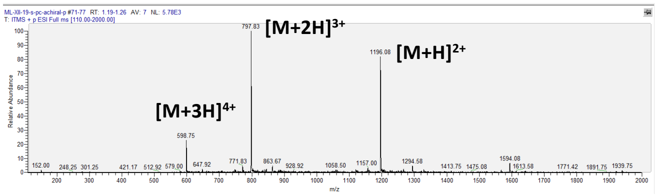

MALDI-TOF-MS: *m/z* expected for $[M+H]^{+}$: 2390.968, *m/z* found: 2390.723.

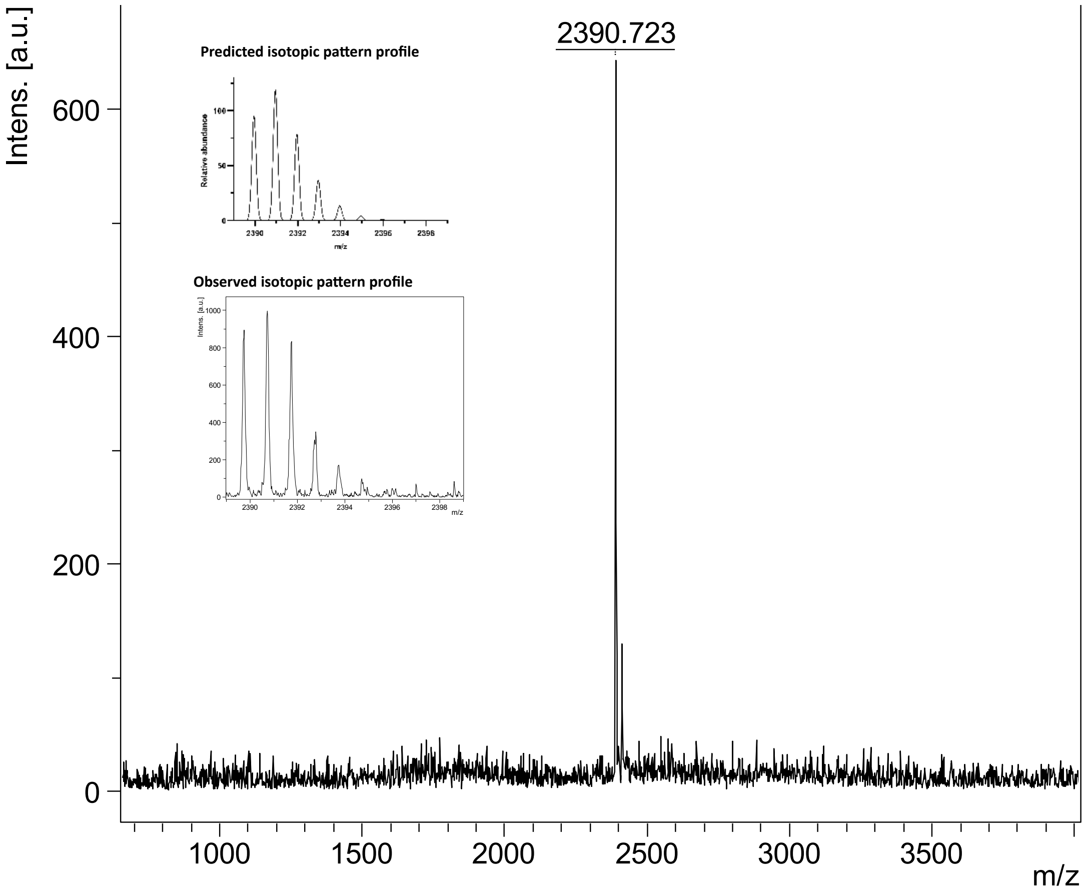

PNA2-pc-H3: C-Ter: $GT^{H}G^{+}$ $AA^{H}T$ $C^{+}C^{H}$ -Ac

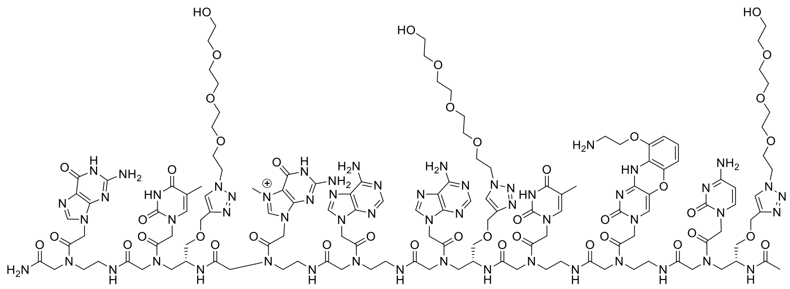

Chemical Formula: $C_{133}H_{184}N_{57}O_{42}^{+}$ **Exact Mass:** 3251.401 **Molecular Weight:** 3253.291

LC-MS-ESI+: *m/z* expected for $[M+3H]^{4+}$: 813.9, *m/z* found: 814.2; *m/z* expected for $[M+2H]^{3+}$: 1084.8, *m/z* found: 1084.9; *m/z* expected for $[M+H]^{2+}$: 1626.7, *m/z* found: 1626.6.

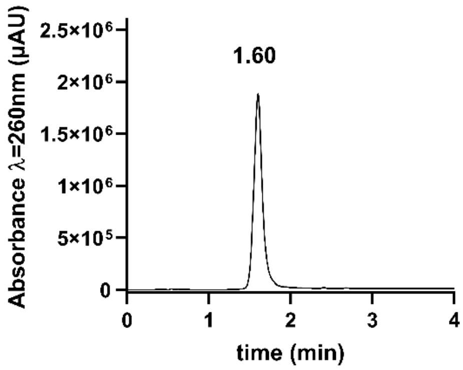

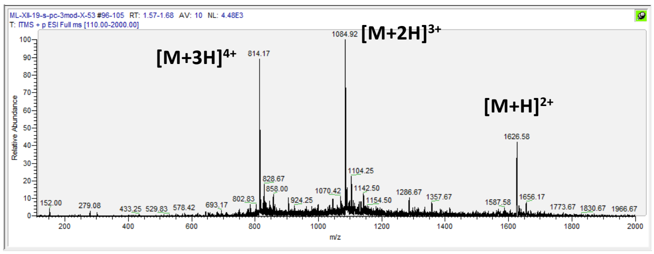

MALDI-TOF-MS: *m/z* expected for $[M+H]^{+}$: 3252.420, *m/z* found: 3252.451.

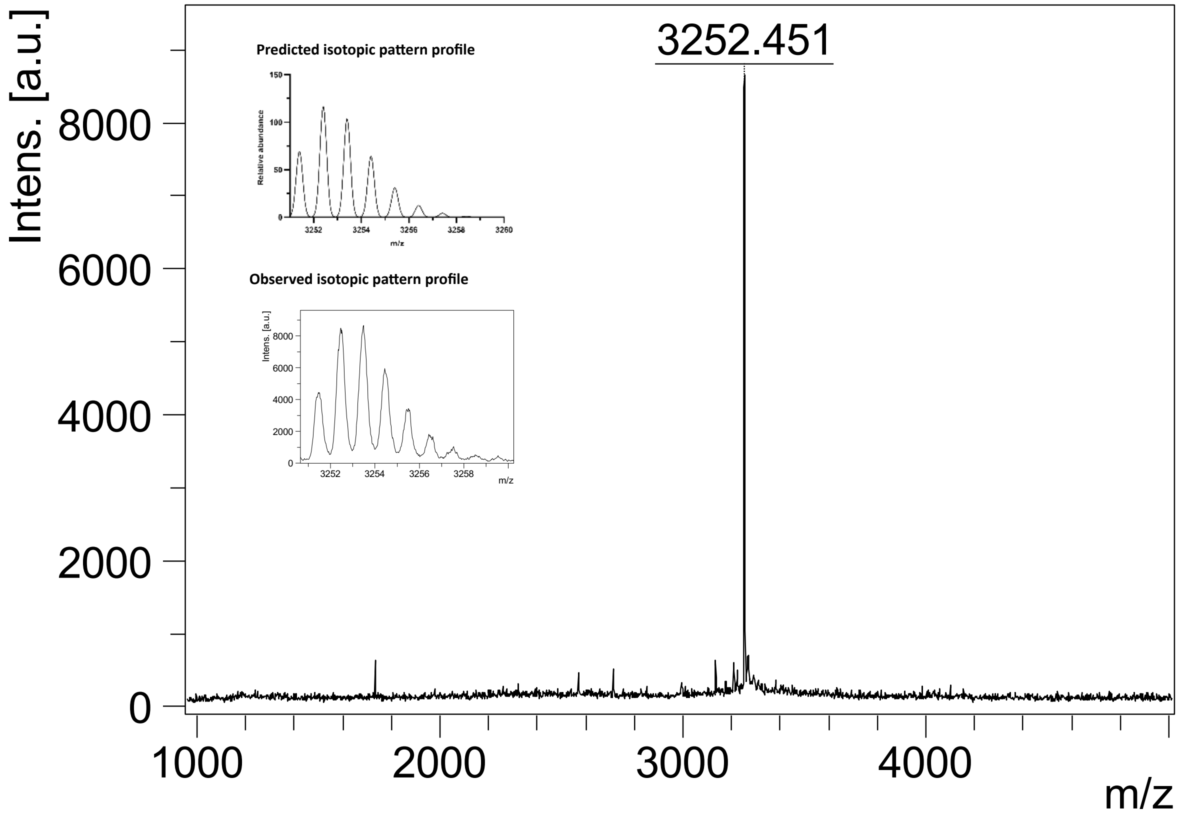

PNA2-pc-A1: C-Ter: GTG+ AAAT C+C -Ac

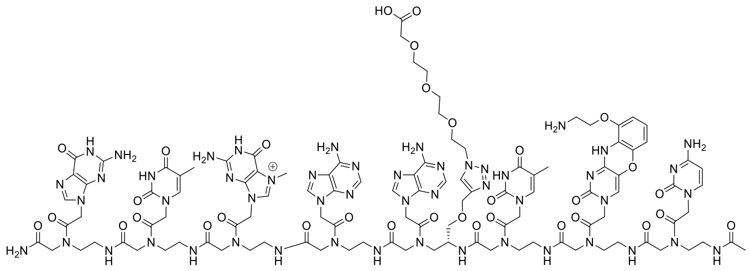

Chemical Formula: $C_{109}H_{140}N_{51}O_{33}^{+}$ **Exact Mass:** 2691.084 **Molecular Weight:** 2692.642

LC-MS-ESI+: *m/z* expected for $[M+3H]^{4+}$: 673.8, *m/z* found: 673.8; *m/z* expected for $[M+2H]^{3+}$: 898.0, *m/z* found: 898.2; *m/z* expected for $[M+H]^{2+}$: 1346.5, *m/z* found: 1346.4.

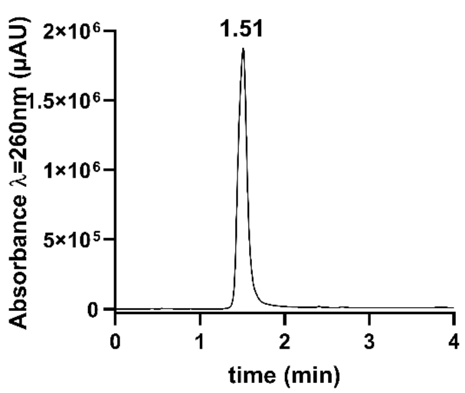

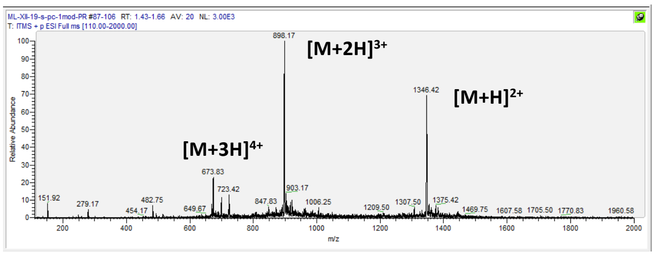

MALDI-TOF-MS: *m/z* expected for $[M+H]^{+}$: 2692.079, *m/z* found: 2692.257.

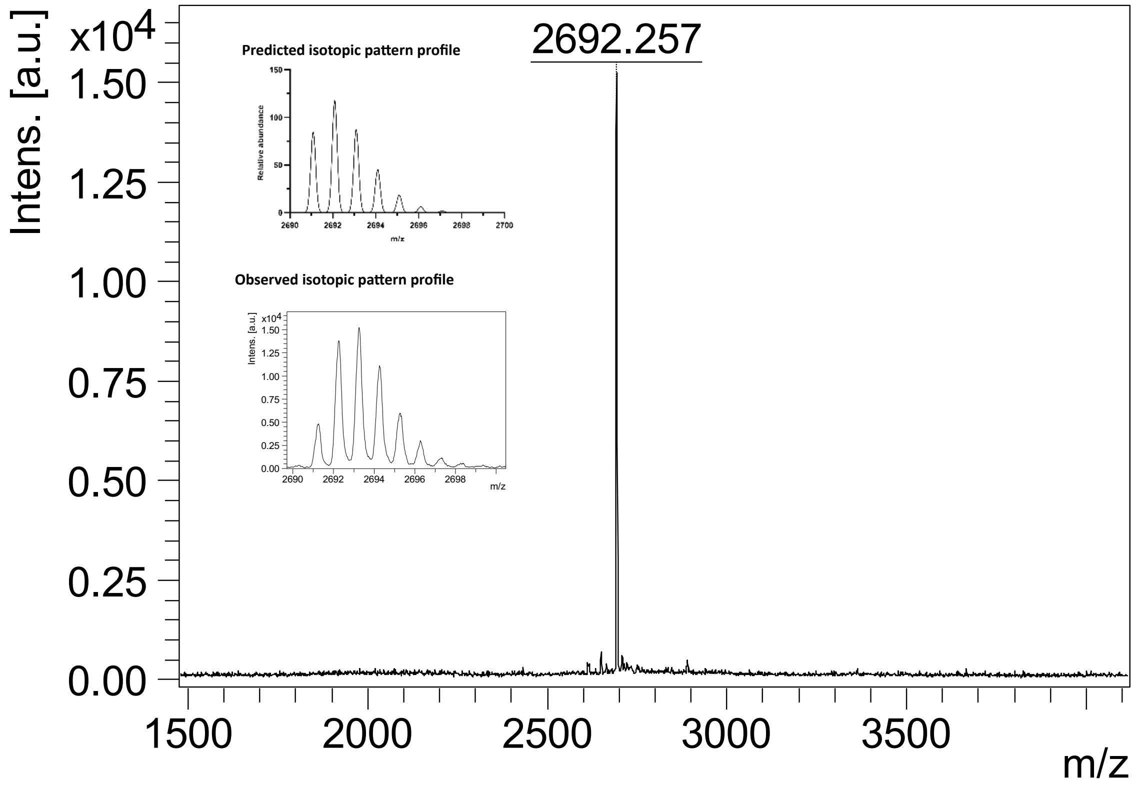

PNA2-pc-A3: C-Ter: GT^{A}G^{+} AA^{A}T C^{+}C^{A} -Ac

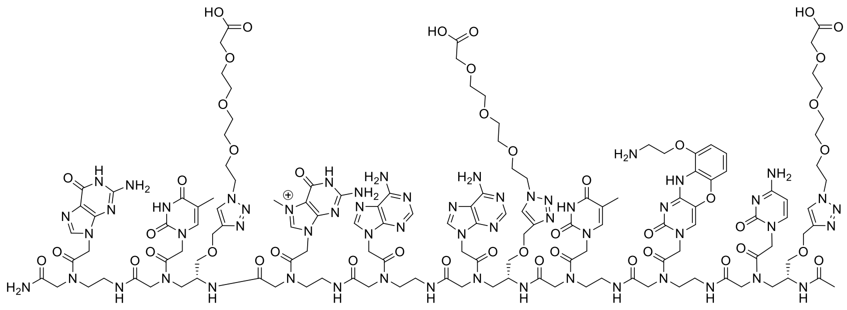

Chemical Formula: $C_{133}H_{178}N_{57}O_{45}^{+}$ **Exact Mass:** 3293.339 **Molecular Weight:** 3295.240

LC-MS-ESI+: *m/z* expected for $[M+3H]^{4+}$: 824.3, *m/z* found: 827.7; *m/z* expected for $[M+2H]^{3+}$: 1098.8, *m/z* found: 1098.9; *m/z* expected for $[M+H]^{2+}$: 1647.7, *m/z* found: 1647.8.

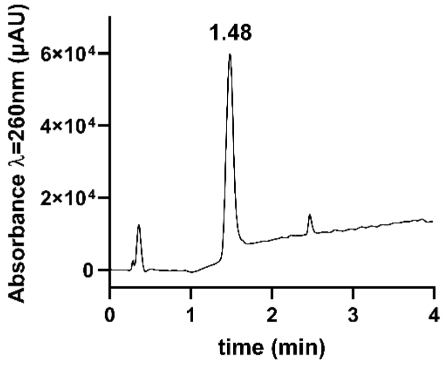

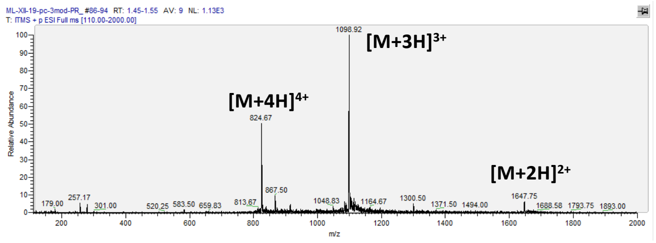

MALDI-TOF-MS: *m/z* expected for $[M+H]^{+}$: 3294.351, *m/z* found: 3294.365.

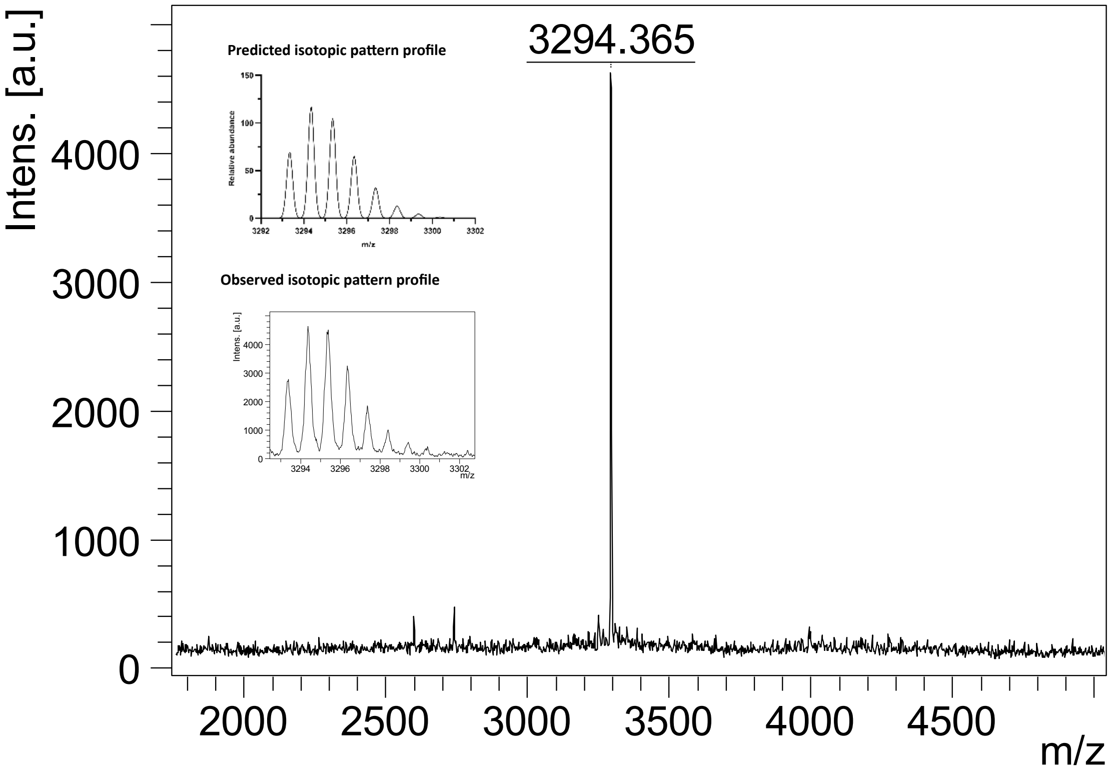

Copies of the SPR response curves and the 1:1 binding model fitting curves.

<u>**DNA6-Bt on chip**</u>

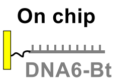

PNA2-ach

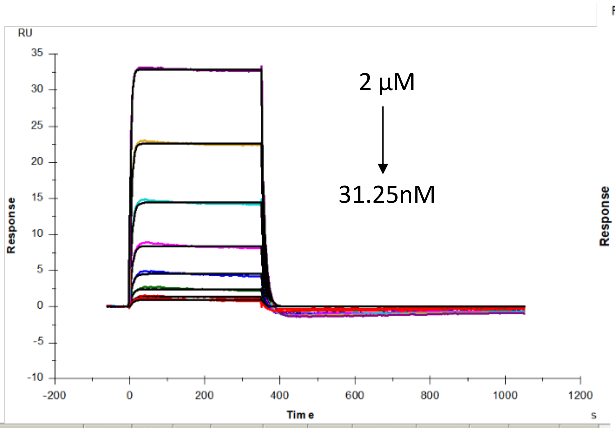

Curve	ka (1/Ms)	kd (1/s)	KD (M)	Rmax (RU)	Conc (M)	tc	Flow (ul/min)	kt (RU/Ms)	RI (RU)	Chi² (RU²)	U-value
	8.872E+4	0.1183	1.333E-6	48.30		1.135E+16				0.412	7
Cycle: 3 31.25 nM					3.125E-8		20.00	3.081E+16	0.2234		
Cycle: 4 62.5 nM					6.250E-8		20.00	3.081E+16	0.2691		
Cycle: 5 125 nM					1.250E-7		20.00	3.081E+16	0.3848		
Cycle: 6 250 nM					2.500E-7		20.00	3.081E+16	0.7863		
Cycle: 7 500 nM					5.000E-7		20.00	3.081E+16	1.234		
Cycle: 8 1000 nM					1.000E-6		20.00	3.081E+16	1.894		
Cycle: 9 2000 nM					2.000E-6		20.00	3.081E+16	3.861		
Cycle: 10 31.25 nM					3.125E-8		20.00	3.081E+16	-0.1420		

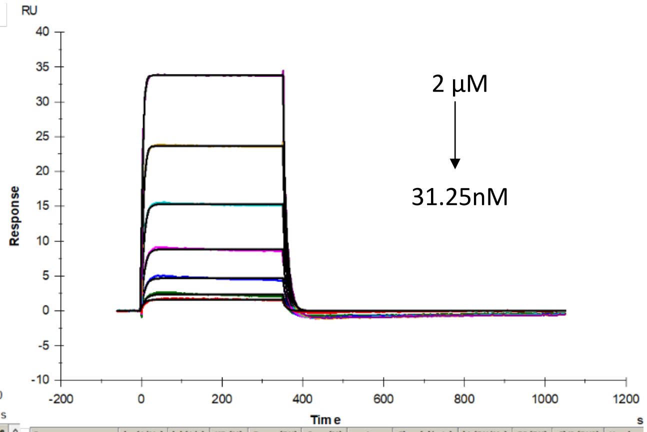

Curve	ka (1/Ms)	kd (1/s)	KD (M)	Rmax (RU)	Conc (M)	tc	Flow (ul/min)	kt (RU/Ms)	RI (RU)	Chi² (RU²)	U-value
	8.892E+4	0.1086	1.222E-6	47.79		8.006E+14				0.303	5
Cycle: 34 62.5 nM					6.250E-8		20.00	2.173E+15	0.01576		
Cycle: 35 125 nM					1.250E-7		20.00	2.173E+15	0.2508		
Cycle: 36 250 nM					2.500E-7		20.00	2.173E+15	0.7020		
Cycle: 37 500 nM					5.000E-7		20.00	2.173E+15	1.430		
Cycle: 38 1000 nM					1.000E-6		20.00	2.173E+15	2.179		
Cycle: 39 2000 nM					2.000E-6		20.00	2.173E+15	4.128		
Cycle: 40 31.25 nM					3.125E-8		20.00	2.173E+15	0.4661		

PNA2-H1

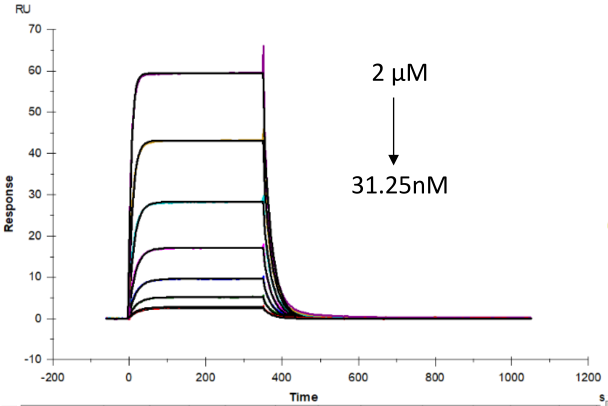

Curve	ka (1/Ms)	kd (1/s)	KD (M)	Rmax (RU)	Conc (M)	tc	Flow (ul/min)	kt (RU/Ms)	RI (RU)	Chi² (RU²)	U-value
	5.005E+4	0.04406	8.805E-7	70.47		6.826E+11				0.141	1
Cycle: 23 31.25 nM					3.125E-8		20.00	1.853E+12	0.1288		
Cycle: 24 62.5 nM					6.250E-8		20.00	1.853E+12	0.5643		
Cycle: 25 125 nM					1.250E-7		20.00	1.853E+12	0.8460		
Cycle: 26 250 nM					2.500E-7		20.00	1.853E+12	1.565		
Cycle: 27 500 nM					5.000E-7		20.00	1.853E+12	2.761		
Cycle: 28 1000 nM					1.000E-6		20.00	1.853E+12	5.662		
Cycle: 29 2000 nM					2.000E-6		20.00	1.853E+12	10.49		
Cycle: 30 31.25 nM					3.125E-8		20.00	1.853E+12	0.3664		

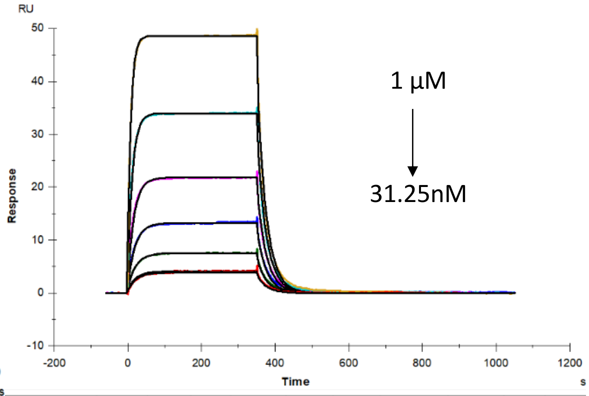

Curve	ka (1/Ms)	kd (1/s)	KD (M)	Rmax (RU)	Conc (M)	tc	Flow (ul/min)	kt (RU/Ms)	RI (RU)	Chi² (
	7.462E+4	0.04010	5.373E-7	62.91		1.192E+12				0
Cycle: 23 31.25 nM					3.125E-8		20.00	3.236E+12	0.6778	
Cycle: 24 62.5 nM					6.250E-8		20.00	3.236E+12	0.9930	
Cycle: 25 125 nM					1.250E-7		20.00	3.236E+12	1.379	
Cycle: 26 250 nM					2.500E-7		20.00	3.236E+12	1.838	
Cycle: 27 500 nM					5.000E-7		20.00	3.236E+12	3.664	
Cycle: 28 1000 nM					1.000E-6		20.00	3.236E+12	7.676	
Cycle: 30 31.25 nM					3.125E-8		20.00	3.236E+12	0.4233	

PNA2-H3

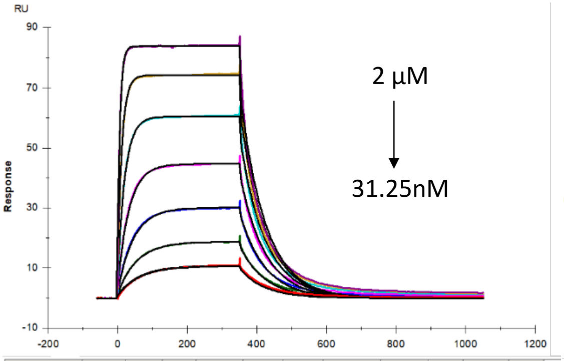

Curve	ka (1/Ms)	kd (1/s)	KD (M)	Rmax (RU)	Conc (M)	tc	Flow (ul/min)	kt (RU/Ms)	RI (RU)	Chi² (RU²)	U-value
	6.536E+4	0.01383	2.116E-7	76.17		2.986E+12				1.24	1
Cycle: 33 31.25 nM					3.125E-8		20.00	8.105E+12	1.042		
Cycle: 34 62.5 nM					6.250E-8		20.00	8.105E+12	1.407		
Cycle: 35 125 nM					1.250E-7		20.00	8.105E+12	1.846		
Cycle: 36 250 nM					2.500E-7		20.00	8.105E+12	3.409		
Cycle: 37 500 nM					5.000E-7		20.00	8.105E+12	6.966		
Cycle: 38 1000 nM					1.000E-6		20.00	8.105E+12	11.32		
Cycle: 39 2000 nM					2.000E-6		20.00	8.105E+12	14.98		
Cycle: 40 31.25 nM					3.125E-8		20.00	8.105E+12	0.8309		

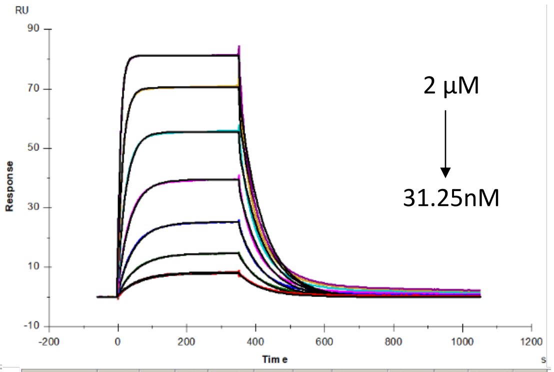

Curve	ka (1/Ms)	kd (1/s)	KD (M)	Rmax (RU)	Conc (M)	tc	Flow (ul/min)	kt (RU/Ms)	RI (RU)	Chi² (RU²)	U-value
	5.090E+4	0.01451	2.850E-7	78.14		9.227E+10				1.44	2
Cycle: 33 31.25 nM					3.125E-8		20.00	2.505E+11	0.2505		
Cycle: 34 62.5 nM					6.250E-8		20.00	2.505E+11	0.6114		
Cycle: 35 125 nM					1.250E-7		20.00	2.505E+11	1.331		
Cycle: 36 250 nM					2.500E-7		20.00	2.505E+11	2.944		
Cycle: 37 500 nM					5.000E-7		20.00	2.505E+11	5.781		
Cycle: 38 1000 nM					1.000E-6		20.00	2.505E+11	9.669		
Cycle: 39 2000 nM					2.000E-6		20.00	2.505E+11	12.81		
Cycle: 40 31.25 nM					3.125E-8		20.00	2.505E+11	0.5854		

PNA2-A3

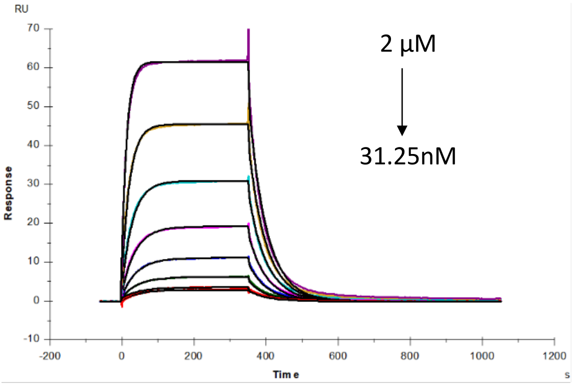

Curve	ka (1/Ms)	kd (1/s)	KD (M)	Rmax (RU)	Conc (M)	tc	Flow (ul/min)	kt (RU/Ms)	RI (RU)	Chi² (RU²)	U-value
	2.537E+4	0.02043	8.052E-7	73.08		2.874E+8				0.435	2
Cycle: 53 31.25 nM					3.125E-8		20.00	7.802E+8	0.2314		
Cycle: 54 62.5 nM					6.250E-8		20.00	7.802E+8	0.9803		
Cycle: 55 125 nM					1.250E-7		20.00	7.802E+8	1.326		
Cycle: 56 250 nM					2.500E-7		20.00	7.802E+8	1.896		
Cycle: 57 500 nM					5.000E-7		20.00	7.802E+8	2.941		
Cycle: 58 1000 nM					1.000E-6		20.00	7.802E+8	5.014		
Cycle: 59 2000 nM					2.000E-6		20.00	7.802E+8	9.468		
Cycle: 60 31.25 nM					3.125E-8		20.00	7.802E+8	0.8046		

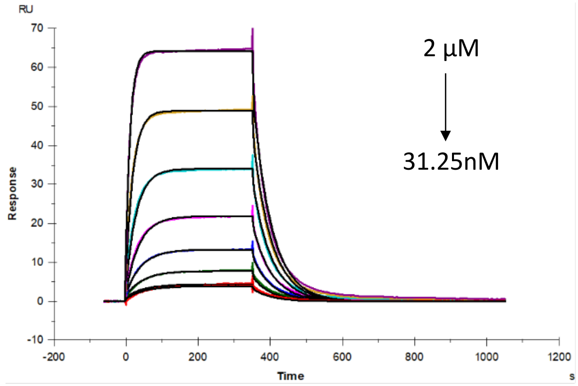

Curve	ka (1/Ms)	kd (1/s)	KD (M)	Rmax (RU)	Conc (M)	tc	Flow (ul/min)	kt (RU/Ms)	RI (RU)	Chi² (RU²)	U-value
	3.020E+4	0.01941	6.429E-7	71.08		1.248E+10				0.411	1
Cycle: 53 31.25 nM					3.125E-8		20.00	3.389E+10	1.088		
Cycle: 54 62.5 nM					6.250E-8		20.00	3.389E+10	1.503		
Cycle: 55 125 nM					1.250E-7		20.00	3.389E+10	1.771		
Cycle: 56 250 nM					2.500E-7		20.00	3.389E+10	1.984		
Cycle: 57 500 nM					5.000E-7		20.00	3.389E+10	2.963		
Cycle: 58 1000 nM					1.000E-6		20.00	3.389E+10	5.678		
Cycle: 59 2000 nM					2.000E-6		20.00	3.389E+10	10.50		
Cycle: 60 31.25 nM					3.125E-8		20.00	3.389E+10	0.6045		

PNA2-N3

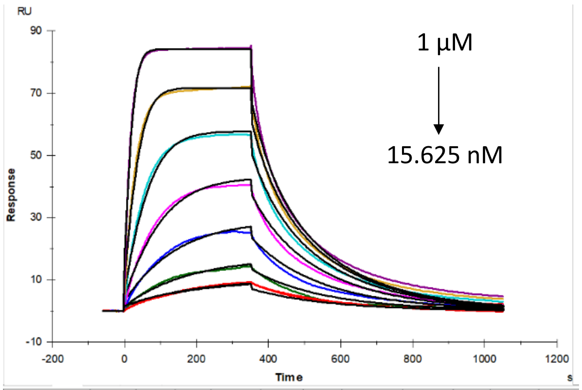

Curve	ka (1/Ms)	kd (1/s)	KD (M)	Rmax (RU)	Conc (M)	tc	Flow (ul/min)	kt (RU/Ms)	RI (RU)	Chi² (RU²)	U-value
	8.237E+4	0.01039	1.261E-7	77.48		1.765E+6				1.41	2
Cycle: 13 15.625 nM					1.563E-8		20.00	4.792E+6	1.486		
Cycle: 14 31.25 nM					3.125E-8		20.00	4.792E+6	1.603		
Cycle: 15 62.5 nM					6.250E-8		20.00	4.792E+6	3.215		
Cycle: 16 125 nM					1.250E-7		20.00	4.792E+6	4.487		
Cycle: 17 250 nM					2.500E-7		20.00	4.792E+6	6.251		
Cycle: 18 500 nM					5.000E-7		20.00	4.792E+6	9.801		
Cycle: 19 1000 nM					1.000E-6		20.00	4.792E+6	15.33		

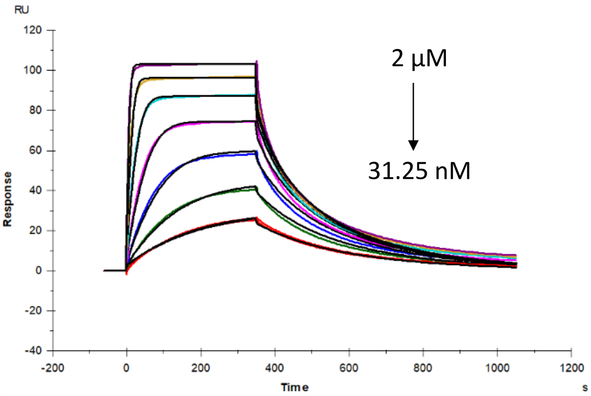

Curve	ka (1/Ms)	kd (1/s)	KD (M)	Rmax (RU)	Conc (M)	tc	Flow (ul/min)	kt (RU/Ms)	RI (RU)	Chi² (RU²)	U-value
	1.563E+5	0.01045	6.685E-8	84.73		2.464E+6				2.23	2
Cycle: 33 31.25 nM					3.125E-8		20.00	6.689E+6	2.453		
Cycle: 34 62.5 nM					6.250E-8		20.00	6.689E+6	3.104		
Cycle: 35 125 nM					1.250E-7		20.00	6.689E+6	4.536		
Cycle: 36 250 nM					2.500E-7		20.00	6.689E+6	7.566		
Cycle: 37 500 nM					5.000E-7		20.00	6.689E+6	12.23		
Cycle: 38 1000 nM					1.000E-6		20.00	6.689E+6	16.71		
Cycle: 39 2000 nM					2.000E-6		20.00	6.689E+6	20.82		

PNA2-G3

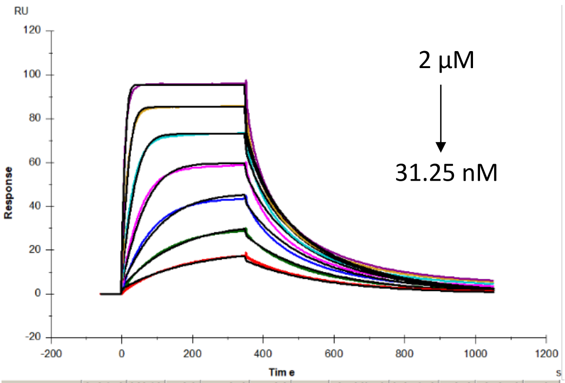

Curve	ka (1/Ms)	kd (1/s)	KD (M)	Rmax (RU)	Conc (M)	tc	Flow (ul/min)	kt (RU/Ms)	RI (RU)	Chi² (RU²)	U-value
	9.938E+4	0.01083	1.089E-7	78.83		1.677E+6				2.04	2
Cycle: 13 31.25 nM					3.125E-8		20.00	4.552E+6	2.358		
Cycle: 14 62.5 nM					6.250E-8		20.00	4.552E+6	3.193		
Cycle: 15 125 nM					1.250E-7		20.00	4.552E+6	3.878		
Cycle: 16 250 nM					2.500E-7		20.00	4.552E+6	4.867		
Cycle: 17 500 nM					5.000E-7		20.00	4.552E+6	8.339		
Cycle: 18 1000 nM					1.000E-6		20.00	4.552E+6	14.17		
Cycle: 19 2000 nM					2.000E-6		20.00	4.552E+6	20.72		
Cycle: 20 31.25 nM					3.125E-8		20.00	4.552E+6	2.323		

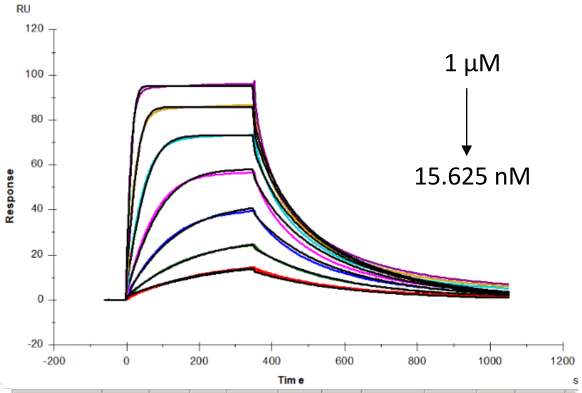

Curve	ka (1/Ms)	kd (1/s)	KD (M)	Rmax (RU)	Conc (M)	tc	Flow (ul/min)	kt (RU/Ms)	RI (RU)	Chi² (RU²)	U
	1.521E+5	0.01050	6.906E-8	84.40		2.440E+6				1.41	
Cycle: 33 15.625 nM					1.563E-8		20.00	6.624E+6	1.250		
Cycle: 34 31.25 nM					3.125E-8		20.00	6.624E+6	1.804		
Cycle: 35 62.5 nM					6.250E-8		20.00	6.624E+6	2.541		
Cycle: 36 125 nM					1.250E-7		20.00	6.624E+6	3.861		
Cycle: 37 250 nM					2.500E-7		20.00	6.624E+6	6.955		
Cycle: 38 500 nM					5.000E-7		20.00	6.624E+6	11.45		
Cycle: 39 1000 nM					1.000E-6		20.00	6.624E+6	16.14		

PNA2-P3

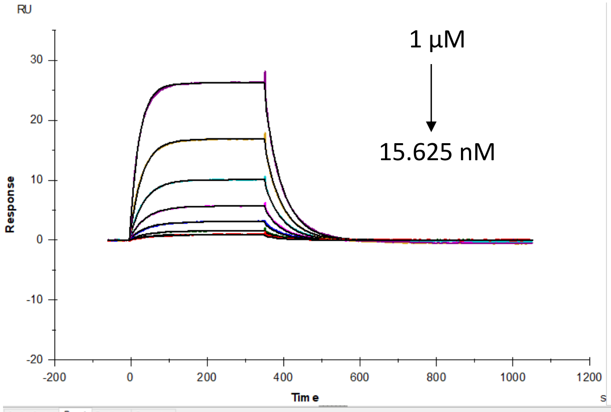

Quality Control | Report | Residuals | Parameters

Curve	ka (1/Ms)	kd (1/s)	KD (M)	Rmax (RU)	Conc (M)	tc	Flow (ul/min)	kt (RU/Ms)	RI (RU)	Chi^2 (RU^2)	U-value
	2.003E+4	0.02141	1.069E-6	49.07		4.167E+8				0.0379	1
Cycle: 53 15.625 nM					1.563E-8		20.00	1.131E+9	0.2328		
Cycle: 54 31.25 nM					3.125E-8		20.00	1.131E+9	0.1663		
Cycle: 55 62.5 nM					6.250E-8		20.00	1.131E+9	0.3705		
Cycle: 56 125 nM					1.250E-7		20.00	1.131E+9	0.5474		
Cycle: 57 250 nM					2.500E-7		20.00	1.131E+9	0.8215		
Cycle: 58 500 nM					5.000E-7		20.00	1.131E+9	1.192		
Cycle: 59 1000 nM					1.000E-6		20.00	1.131E+9	2.464		
Cycle: 60 15.625 nM					1.563E-8		20.00	1.131E+9	0.1382		

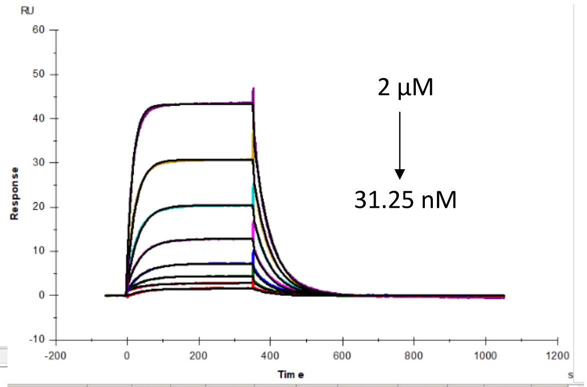

Curve	ka (1/Ms)	kd (1/s)	KD (M)	Rmax (RU)	Conc (M)	tc	Flow (ul/min)	kt (RU/Ms)	RI (RU)	Chi^2 (RU^2)	U-value
	1.960E+4	0.02050	1.046E-6	56.18		1.087E+10				0.169	1
Cycle: 53 31.25 nM					3.125E-8		20.00	2.950E+10	1.180		
Cycle: 54 62.5 nM					6.250E-8		20.00	2.950E+10	1.279		
Cycle: 55 125 nM					1.250E-7		20.00	2.950E+10	1.241		
Cycle: 56 250 nM					2.500E-7		20.00	2.950E+10	1.992		
Cycle: 57 500 nM					5.000E-7		20.00	2.950E+10	2.301		
Cycle: 58 1000 nM					1.000E-6		20.00	2.950E+10	3.320		
Cycle: 59 2000 nM					2.000E-6		20.00	2.950E+10	6.399		
Cycle: 60 31.25 nM					3.125E-8		20.00	2.950E+10	0.01549		

DNA8

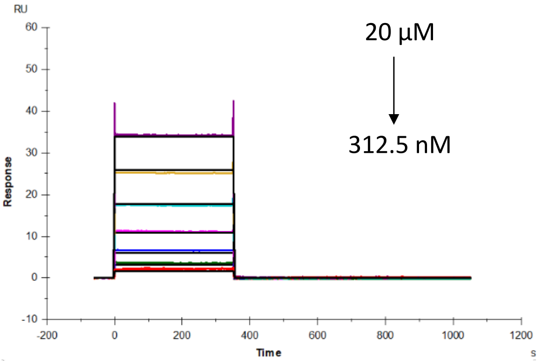

Curve	ka (1/Ms)	kd (1/s)	KD (M)	Rmax (RU)	Conc (M)	Chi^2 (RU^2)	U-value
	3.739E+5	3.250	8.692E-6	48.53		0.777	N/A
Cycle: 23 312.5 nM					3.125E-7		
Cycle: 24 625 nM					6.250E-7		
Cycle: 25 1250 nM					1.250E-6		
Cycle: 26 2500 nM					2.500E-6		
Cycle: 27 5000 nM					5.000E-6		
Cycle: 28 10000 nM					1.000E-5		
Cycle: 29 20000 nM					2.000E-5		
Cycle: 30 312.5 nM					3.125E-7		

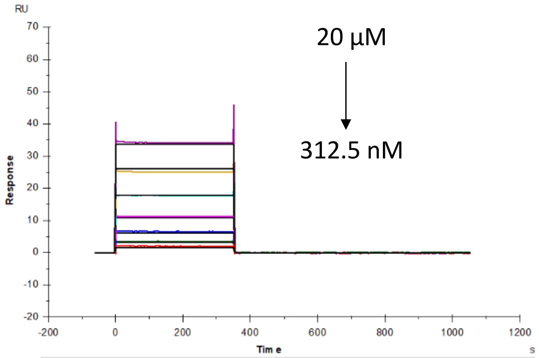

Curve	ka (1/Ms)	kd (1/s)	KD (M)	Rmax (RU)	Conc (M)	Chi^2 (RU^2)	U-value
	3.970E+5	3.335	8.400E-6	47.96		0.805	N/A
Cycle: 53 312.5 nM					3.125E-7		
Cycle: 54 625 nM					6.250E-7		
Cycle: 55 1250 nM					1.250E-6		
Cycle: 56 2500 nM					2.500E-6		
Cycle: 57 5000 nM					5.000E-6		
Cycle: 58 10000 nM					1.000E-5		
Cycle: 59 20000 nM					2.000E-5		
Cycle: 60 312.5 nM					3.125E-7		

PNA2-pc-ach

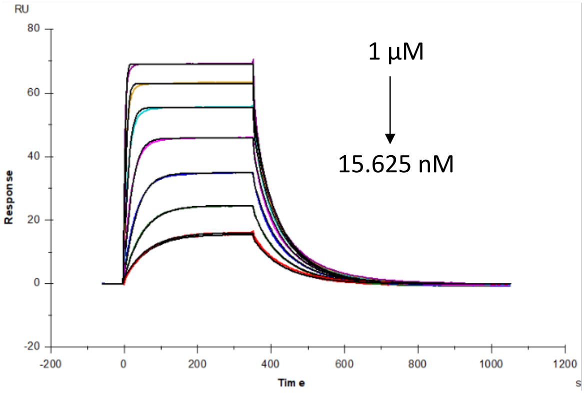

Curve	ka (1/Ms)	kd (1/s)	KD (M)	Rmax (RU)	Conc (M)	tc	Flow (ul/min)	kt (RU/Ms)	RI (RU)	Chi^2 (RU^2)	U-value
	3.860E+5	0.01873	4.854E-8	58.76		1.247E+7				0.267	1
Cycle: 13 15.625 nM					1.563E-8		20.00	3.384E+7	1.212		
Cycle: 14 31.25 nM					3.125E-8		20.00	3.384E+7	1.607		
Cycle: 15 62.5 nM					6.250E-8		20.00	3.384E+7	1.825		
Cycle: 16 125 nM					1.250E-7		20.00	3.384E+7	3.467		
Cycle: 17 250 nM					2.500E-7		20.00	3.384E+7	6.180		
Cycle: 18 500 nM					5.000E-7		20.00	3.384E+7	9.505		
Cycle: 19 1000 nM					1.000E-6		20.00	3.384E+7	13.13		
Cycle: 20 15.625 nM					1.563E-8		20.00	3.384E+7	1.732		

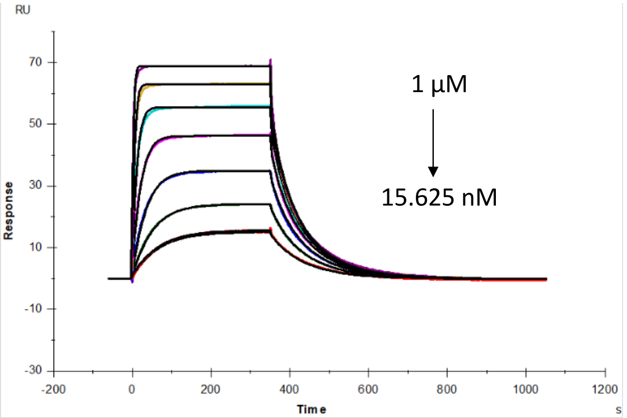

Curve	ka (1/Ms)	kd (1/s)	KD (M)	Rmax (RU)	Conc (M)	tc	Flow (ul/min)	kt (RU/Ms)	RI (RU)	Chi^2 (RU^2)	U-value
	3.902E+5	0.01862	4.771E-8	58.50		1.168E+7				0.246	1
Cycle: 43 15.625 nM					1.563E-8		20.00	3.171E+7	0.6966		
Cycle: 44 31.25 nM					3.125E-8		20.00	3.171E+7	1.008		
Cycle: 45 62.5 nM					6.250E-8		20.00	3.171E+7	1.733		
Cycle: 46 125 nM					1.250E-7		20.00	3.171E+7	3.874		
Cycle: 47 250 nM					2.500E-7		20.00	3.171E+7	6.363		
Cycle: 48 500 nM					5.000E-7		20.00	3.171E+7	9.526		
Cycle: 49 1000 nM					1.000E-6		20.00	3.171E+7	12.93		
Cycle: 50 15.625 nM					1.563E-8		20.00	3.171E+7	1.198		

PNA2-pc-H3

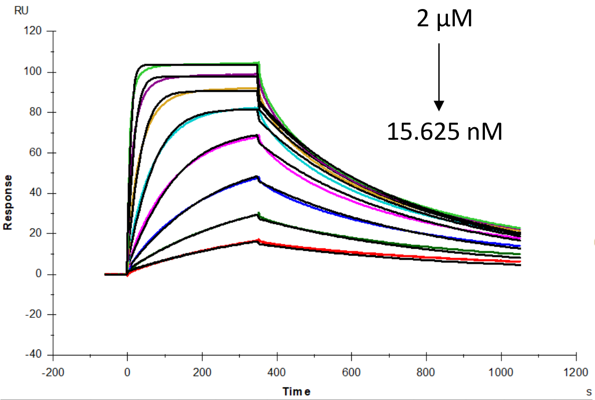

Curve	ka (1/Ms)	kd (1/s)	KD (M)	Rmax (RU)	Conc (M)	tc	Flow (ul/min)	kt (RU/Ms)	RI (RU)	Chi² (RU²)	U-value
	7.491E+4	0.002749	3.669E-8	87.51		3.461E+6				2.02	1
Cycle: 36 15.625 nM					1.563E-8		20.00	9.394E+6	1.654		
Cycle: 37 31.25 nM					3.125E-8		20.00	9.394E+6	2.262		
Cycle: 38 62.5 nM					6.250E-8		20.00	9.394E+6	2.746		
Cycle: 39 125 nM					1.250E-7		20.00	9.394E+6	3.883		
Cycle: 40 250 nM					2.500E-7		20.00	9.394E+6	5.316		
Cycle: 41 500 nM					5.000E-7		20.00	9.394E+6	8.911		
Cycle: 42 1000 nM					1.000E-6		20.00	9.394E+6	13.16		
Cycle: 43 2000 nM					2.000E-6		20.00	9.394E+6	17.46		
Cycle: 44 15.625 nM					1.563E-8		20.00	9.394E+6	1.436		

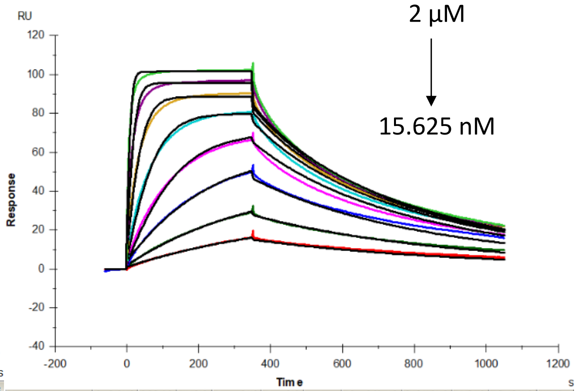

Curve	ka (1/Ms)	kd (1/s)	KD (M)	Rmax (RU)	Conc (M)	tc	Flow (ul/min)	kt (RU/Ms)	RI (RU)	Chi² (RU²)	U-value
	8.279E+4	0.002835	3.425E-8	85.97		3.108E+6				2.22	1
Cycle: 36 15.625 nM					1.563E-8		20.00	8.437E+6	1.161		
Cycle: 37 31.25 nM					3.125E-8		20.00	8.437E+6	1.983		
Cycle: 38 62.5 nM					6.250E-8		20.00	8.437E+6	4.192		
Cycle: 39 125 nM					1.250E-7		20.00	8.437E+6	2.605		
Cycle: 40 250 nM					2.500E-7		20.00	8.437E+6	4.221		
Cycle: 41 500 nM					5.000E-7		20.00	8.437E+6	8.173		
Cycle: 42 1000 nM					1.000E-6		20.00	8.437E+6	12.45		
Cycle: 43 2000 nM					2.000E-6		20.00	8.437E+6	16.93		
Cycle: 44 15.625 nM					1.563E-8		20.00	8.437E+6	1.111		

PNA2-pc-A1

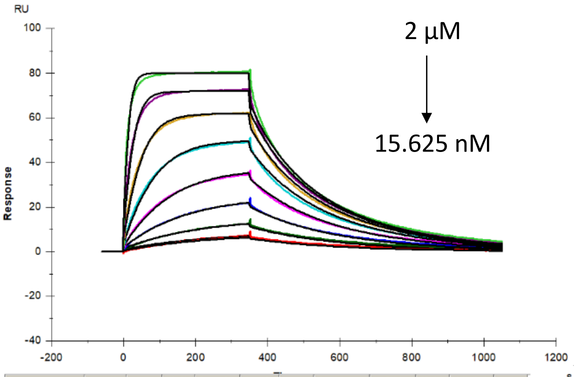

Curve	ka (1/Ms)	kd (1/s)	KD (M)	Rmax (RU)	Conc (M)	tc	Flow (ul/min)	kt (RU/Ms)	RI (RU)	Chi² (RU²)	U-value
	4.599E+4	0.005999	1.304E-7	70.56		2.009E+6				0.698	1
Cycle: 47 15.625 nM					1.563E-8		20.00	5.453E+6	1.096		
Cycle: 48 31.25 nM					3.125E-8		20.00	5.453E+6	1.455		
Cycle: 49 62.5 nM					6.250E-8		20.00	5.453E+6	2.064		
Cycle: 50 125 nM					1.250E-7		20.00	5.453E+6	2.655		
Cycle: 51 250 nM					2.500E-7		20.00	5.453E+6	3.581		
Cycle: 52 500 nM					5.000E-7		20.00	5.453E+6	5.879		
Cycle: 53 1000 nM					1.000E-6		20.00	5.453E+6	9.440		
Cycle: 54 2000 nM					2.000E-6		20.00	5.453E+6	13.73		
Cycle: 55 15.625 nM					1.563E-8		20.00	5.453E+6	0.4996		

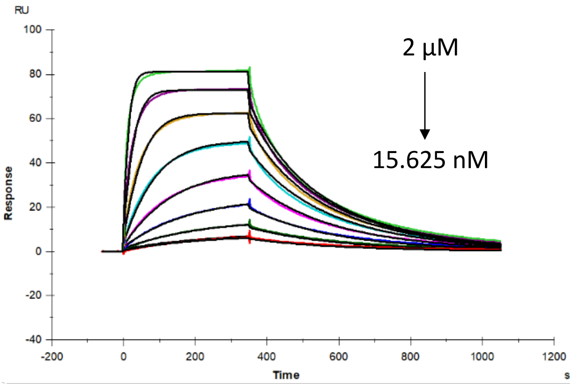

Curve	ka (1/Ms)	kd (1/s)	KD (M)	Rmax (RU)	Conc (M)	tc	Flow (ul/min)	kt (RU/Ms)	RI (RU)	Chi² (RU²)	U-value
	4.304E+4	0.005982	1.390E-7	71.65		1.974E+6				0.707	1
Cycle: 47 15.625 nM					1.563E-8		20.00	5.358E+6	1.015		
Cycle: 48 31.25 nM					3.125E-8		20.00	5.358E+6	1.347		
Cycle: 49 62.5 nM					6.250E-8		20.00	5.358E+6	1.946		
Cycle: 50 125 nM					1.250E-7		20.00	5.358E+6	2.637		
Cycle: 51 250 nM					2.500E-7		20.00	5.358E+6	3.812		
Cycle: 52 500 nM					5.000E-7		20.00	5.358E+6	6.189		
Cycle: 53 1000 nM					1.000E-6		20.00	5.358E+6	9.819		
Cycle: 54 2000 nM					2.000E-6		20.00	5.358E+6	13.94		
Cycle: 55 15.625 nM					1.563E-8		20.00	5.358E+6	0.5221		

PNA2-pc-A3

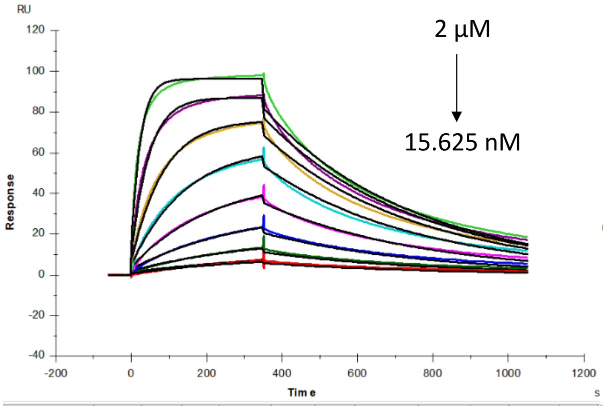

Curve	ka (1/Ms)	kd (1/s)	KD (M)	Rmax (RU)	Conc (M)	tc	Flow (ul/min)	kt (RU/Ms)	RI (RU)	Chi² (RU²)	U-value
	2.052E+4	0.002548	1.242E-7	86.75		5.905E+6				1.96	1
Cycle: 58 15.625 nM					1.563E-8		20.00	1.603E+7	1.300		
Cycle: 59 31.25 nM					3.125E-8		20.00	1.603E+7	1.988		
Cycle: 60 62.5 nM					6.250E-8		20.00	1.603E+7	2.860		
Cycle: 61 125 nM					1.250E-7		20.00	1.603E+7	3.888		
Cycle: 62 250 nM					2.500E-7		20.00	1.603E+7	4.898		
Cycle: 63 500 nM					5.000E-7		20.00	1.603E+7	6.553		
Cycle: 64 1000 nM					1.000E-6		20.00	1.603E+7	9.784		
Cycle: 65 2000 nM					2.000E-6		20.00	1.603E+7	14.49		
Cycle: 66 15.625 nM					1.563E-8		20.00	1.603E+7	0.7069		

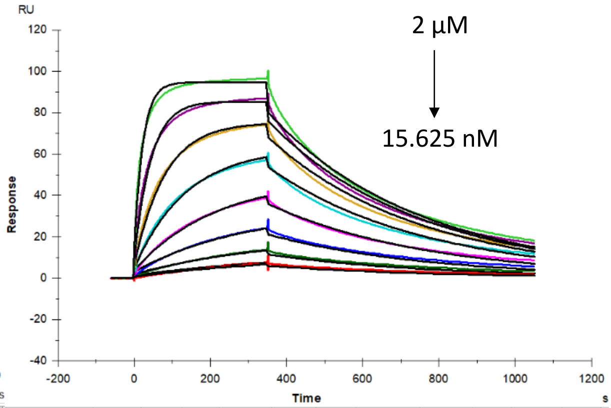

Curve	ka (1/Ms)	kd (1/s)	KD (M)	Rmax (RU)	Conc (M)	tc	Flow (ul/min)	kt (RU/Ms)	RI (RU)	Chi² (RU²)	U-value
	2.184E+4	0.002548	1.167E-7	85.10		6.001E+6				1.94	1
Cycle: 58 15.625 nM					1.563E-8		20.00	1.629E+7	1.373		
Cycle: 59 31.25 nM					3.125E-8		20.00	1.629E+7	1.996		
Cycle: 60 62.5 nM					6.250E-8		20.00	1.629E+7	3.061		
Cycle: 61 125 nM					1.250E-7		20.00	1.629E+7	3.783		
Cycle: 62 250 nM					2.500E-7		20.00	1.629E+7	4.837		
Cycle: 63 500 nM					5.000E-7		20.00	1.629E+7	6.354		
Cycle: 64 1000 nM					1.000E-6		20.00	1.629E+7	9.381		
Cycle: 65 2000 nM					2.000E-6		20.00	1.629E+7	14.38		
Cycle: 66 15.625 nM					1.563E-8		20.00	1.629E+7	0.7005		

PNA3-Bt on chip

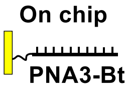

PNA2-ach

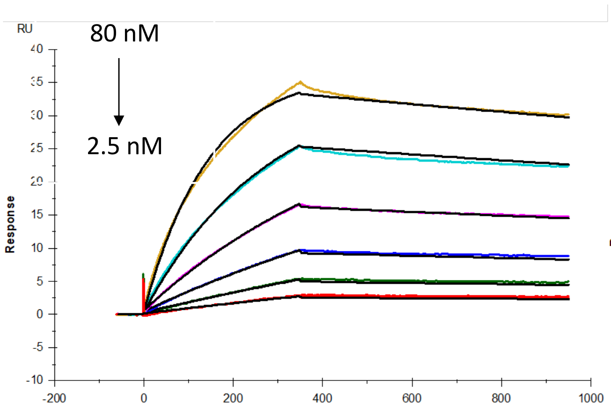

Curve	ka (1/Ms)	kd (1/s)	KD (M)	Rmax (RU)	Conc (M)	tc	Flow (ul/min)	kt (RU/Ms)	RI (RU)	Chi² (RU²)	U-value
	8.413E+4	1.860E-4	2.211E-9	37.50		5.526E+19				0.124	2
Cycle: 12 2.5 nM					2.500E-9		20.00	1.500E+20	0.2059		
Cycle: 13 5 nM					5.000E-9		20.00	1.500E+20	0.3454		
Cycle: 14 10 nM					1.000E-8		20.00	1.500E+20	0.4823		
Cycle: 15 20 nM					2.000E-8		20.00	1.500E+20	0.5779		
Cycle: 16 40 nM					4.000E-8		20.00	1.500E+20	0.2977		
Cycle: 17 80 nM					8.000E-8		20.00	1.500E+20	0.2850		
Cycle: 18 2.5 nM					2.500E-9		20.00	1.500E+20	0.2024		

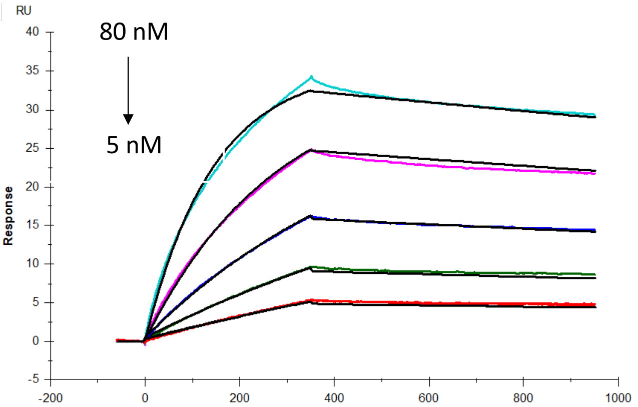

Curve	ka (1/Ms)	kd (1/s)	KD (M)	Rmax (RU)	Conc (M)	tc	Flow (ul/min)	kt (RU/Ms)	RI (RU)	Chi² (RU²)	U-value
	8.461E+4	1.854E-4	2.191E-9	36.50		3.049E+14				0.142	2
Cycle: 12 5 nM					5.000E-9		20.00	8.277E+14	0.3685		
Cycle: 13 10 nM					1.000E-8		20.00	8.277E+14	0.5071		
Cycle: 14 20 nM					2.000E-8		20.00	8.277E+14	0.4956		
Cycle: 15 40 nM					4.000E-8		20.00	8.277E+14	0.1962		
Cycle: 16 80 nM					8.000E-8		20.00	8.277E+14	0.1265		
Cycle: 17 5 nM					5.000E-9		20.00	8.277E+14	0.3133		

PNA2-H1

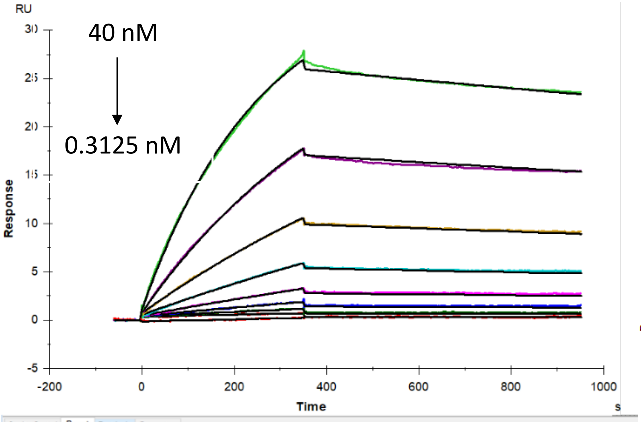

Quality Control | Report | Residuals | Parameters

Curve	ka (1/Ms)	kd (1/s)	KD (M)	Rmax (RU)	Conc (M)	tc	Flow (ul/min)	kt (RU/Ms)	RI (RU)	Chi² (RU²)	U-value
	9.437E+4	1.764E-4	1.869E-9	36.23		5.316E+18				0.0437	2
Cycle: 37 0.3125 nM					3.125E-10		20.00	1.443E+19	0.3567		
Cycle: 38 0.625 nM					6.250E-10		20.00	1.443E+19	0.4434		
Cycle: 39 1.25 nM					1.250E-9		20.00	1.443E+19	0.4630		
Cycle: 40 2.5 nM					2.500E-9		20.00	1.443E+19	0.4931		
Cycle: 41 5 nM					5.000E-9		20.00	1.443E+19	0.5613		
Cycle: 42 10 nM					1.000E-8		20.00	1.443E+19	0.7206		
Cycle: 43 20 nM					2.000E-8		20.00	1.443E+19	0.7769		
Cycle: 44 40 nM					4.000E-8		20.00	1.443E+19	1.032		

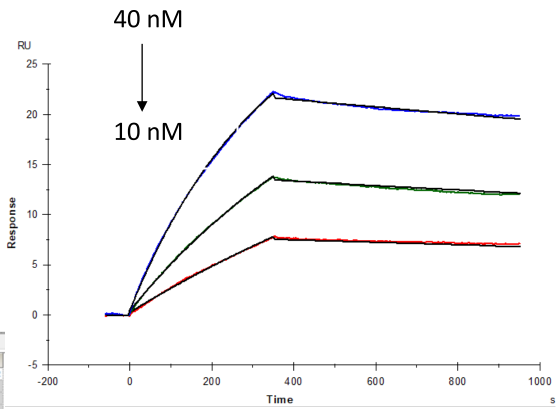

Curve	ka (1/Ms)	kd (1/s)	KD (M)	Rmax (RU)	Conc (M)	tc	Flow (ul/min)	kt (RU/Ms)	RI (RU)	Chi² (RU²)	U-value
	7.180E+4	1.717E-4	2.391E-9	34.98		2.063E+13				0.0215	1
Cycle: 31 10 nM					1.000E-8		20.00	5.601E+13	0.2726		
Cycle: 32 20 nM					2.000E-8		20.00	5.601E+13	0.4394		
Cycle: 33 40 nM					4.000E-8		20.00	5.601E+13	0.5047		

PNA2-H3

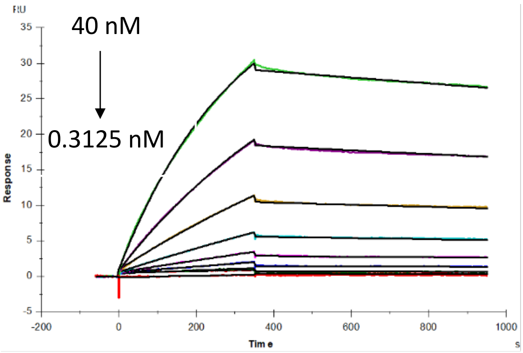

Curve	ka (1/Ms)	kd (1/s)	KD (M)	Rmax (RU)	Conc (M)	tc	Flow (ul/min)	kt (RU/Ms)	RI (RU)	Chi^2 (RU^2)	U-value
	7.968E+4	1.494E-4	1.875E-9	44.11		2.512E+17				0.0668	3
Cycle: 48 0.3125 nM					3.125E-10		20.00	6.818E+17	0.5595		
Cycle: 49 0.625 nM					6.250E-10		20.00	6.818E+17	0.4402		
Cycle: 50 1.25 nM					1.250E-9		20.00	6.818E+17	0.5566		
Cycle: 51 2.5 nM					2.500E-9		20.00	6.818E+17	0.5892		
Cycle: 52 5 nM					5.000E-9		20.00	6.818E+17	0.6423		
Cycle: 53 10 nM					1.000E-8		20.00	6.818E+17	0.9734		
Cycle: 54 20 nM					2.000E-8		20.00	6.818E+17	0.8853		
Cycle: 55 40 nM					4.000E-8		20.00	6.818E+17	1.012		

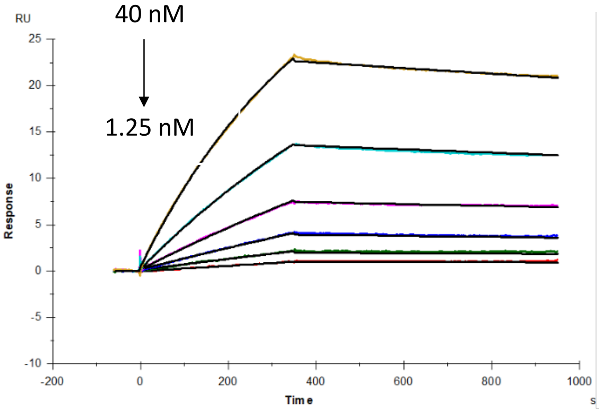

Curve	ka (1/Ms)	kd (1/s)	KD (M)	Rmax (RU)	Conc (M)	tc	Flow (ul/min)	kt (RU/Ms)	RI (RU)	Chi^2 (RU^2)	U-value
	5.849E+4	1.381E-4	2.361E-9	41.31		1.609E+17				0.0142	2
Cycle: 39 1.25 nM					1.250E-9		20.00	4.367E+17	-0.01018		
Cycle: 40 2.5 nM					2.500E-9		20.00	4.367E+17	0.1462		
Cycle: 41 5 nM					5.000E-9		20.00	4.367E+17	0.1932		
Cycle: 42 10 nM					1.000E-8		20.00	4.367E+17	0.1793		
Cycle: 43 20 nM					2.000E-8		20.00	4.367E+17	0.1857		
Cycle: 44 40 nM					4.000E-8		20.00	4.367E+17	0.3908		

PNA2-A3

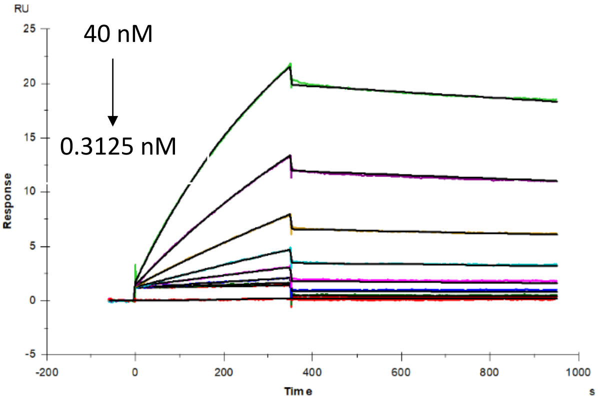

Curve	ka (1/Ms)	kd (1/s)	KD (M)	Rmax (RU)	Conc (M)	tc	Flow (ul/min)	kt (RU/Ms)	RI (RU)	Chi^2 (RU^2)	U-value
	5.972E+4	1.355E-4	2.268E-9	35.77		5.136E+12				0.187	9
Cycle: 70 0.3125 nM					3.125E-10		20.00	1.394E+13	1.194		
Cycle: 71 0.625 nM					6.250E-10		20.00	1.394E+13	1.194		
Cycle: 72 1.25 nM					1.250E-9		20.00	1.394E+13	1.203		
Cycle: 73 2.5 nM					2.500E-9		20.00	1.394E+13	1.307		
Cycle: 74 5 nM					5.000E-9		20.00	1.394E+13	1.254		
Cycle: 75 10 nM					1.000E-8		20.00	1.394E+13	1.374		
Cycle: 76 20 nM					2.000E-8		20.00	1.394E+13	1.463		
Cycle: 77 40 nM					4.000E-8		20.00	1.394E+13	1.750		

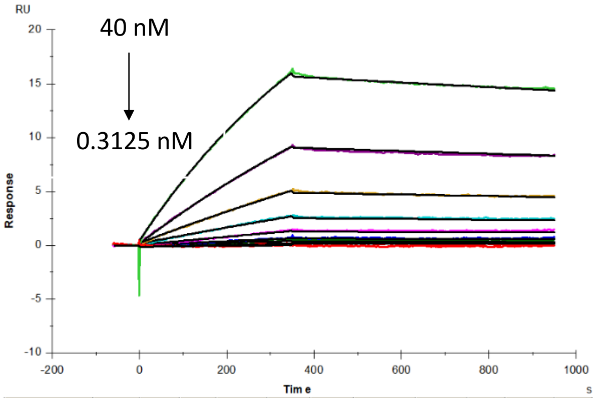

Curve	ka (1/Ms)	kd (1/s)	KD (M)	Rmax (RU)	Conc (M)	tc	Flow (ul/min)	kt (RU/Ms)	RI (RU)	Chi^2 (RU^2)	U-value
	4.562E+4	1.433E-4	3.141E-9	33.93		9.488E+14				0.0126	3
Cycle: 59 0.3125 nM					3.125E-10		20.00	2.575E+15	3.942E-4		
Cycle: 60 0.625 nM					6.250E-10		20.00	2.575E+15	0.08497		
Cycle: 61 1.25 nM					1.250E-9		20.00	2.575E+15	0.009180		
Cycle: 62 2.5 nM					2.500E-9		20.00	2.575E+15	0.04633		
Cycle: 63 5 nM					5.000E-9		20.00	2.575E+15	0.1697		
Cycle: 64 10 nM					1.000E-8		20.00	2.575E+15	0.2155		
Cycle: 65 20 nM					2.000E-8		20.00	2.575E+15	0.1516		
Cycle: 66 40 nM					4.000E-8		20.00	2.575E+15	0.4059		

PNA2-N3

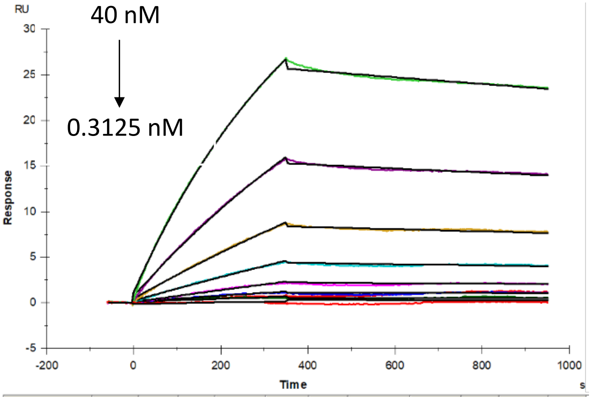

Curve	ka (1/Ms)	kd (1/s)	KD (M)	Rmax (RU)	Conc (M)	tc	Flow (ul/min)	kt (RU/Ms)	RI (RU)	Chi^2 (RU^2)	U-value
	5.663E+4	1.513E-4	2.672E-9	48.16		8.854E+7				0.0586	4
Cycle: 15 0.3125 nM					3.125E-10		20.00	2.403E+8	0.3342		
Cycle: 16 0.625 nM					6.250E-10		20.00	2.403E+8	0.1136		
Cycle: 17 1.25 nM					1.250E-9		20.00	2.403E+8	0.1153		
Cycle: 18 2.5 nM					2.500E-9		20.00	2.403E+8	0.08813		
Cycle: 19 5 nM					5.000E-9		20.00	2.403E+8	0.2414		
Cycle: 20 10 nM					1.000E-8		20.00	2.403E+8	0.4646		
Cycle: 21 20 nM					2.000E-8		20.00	2.403E+8	0.7389		
Cycle: 22 40 nM					4.000E-8		20.00	2.403E+8	1.126		

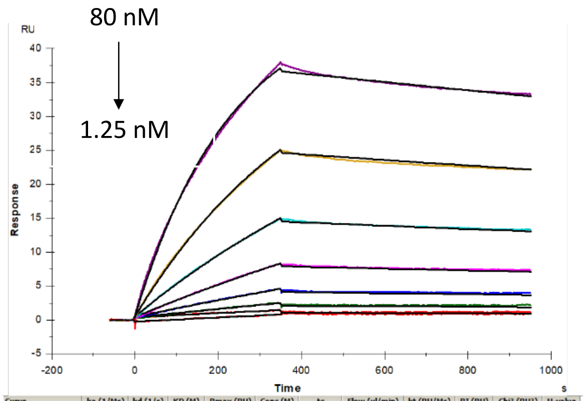

Curve	ka (1/Ms)	kd (1/s)	KD (M)	Rmax (RU)	Conc (M)	tc	Flow (ul/min)	kt (RU/Ms)	RI (RU)	Chi^2 (RU^2)	U-value
	5.206E+4	1.769E-4	3.397E-9	48.87		1.164E+16				0.0437	2
Cycle: 14 1.25 nM					1.250E-9		20.00	3.159E+16	0.4195		
Cycle: 15 2.5 nM					2.500E-9		20.00	3.159E+16	0.4000		
Cycle: 16 5 nM					5.000E-9		20.00	3.159E+16	0.4920		
Cycle: 17 10 nM					1.000E-8		20.00	3.159E+16	0.4726		
Cycle: 18 20 nM					2.000E-8		20.00	3.159E+16	0.5361		
Cycle: 19 40 nM					4.000E-8		20.00	3.159E+16	0.5127		
Cycle: 20 80 nM					8.000E-8		20.00	3.159E+16	0.5282		
Cycle: 21 1.25 nM					1.250E-9		20.00	3.159E+16	-0.2310		

PNA2-G3

40 nM

0.3125 nM

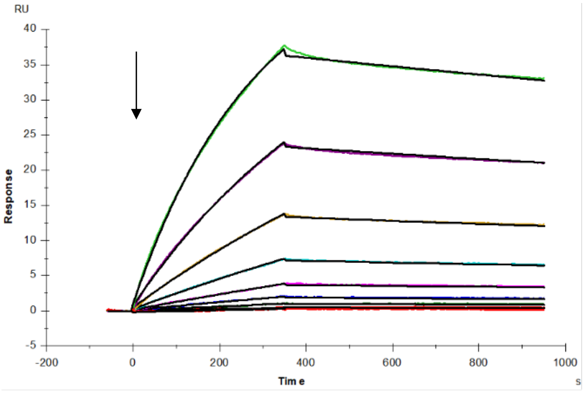

Curve	ka (1/Ms)	kd (1/s)	KD (M)	Rmax (RU)	Conc (M)	tc	Flow (ul/min)	kt (RU/Ms)	RI (RU)	Chi^2 (RU^2)	U-value
	8.486E+4	1.706E-4	2.011E-9	53.40		1.922E+17				0.0193	1
Cycle: 37 0.3125 nM					3.125E-10		20.00	5.218E+17	-0.003214		
Cycle: 38 0.625 nM					6.250E-10		20.00	5.218E+17	0.08495		
Cycle: 39 1.25 nM					1.250E-9		20.00	5.218E+17	0.1522		
Cycle: 40 2.5 nM					2.500E-9		20.00	5.218E+17	0.1803		
Cycle: 41 5 nM					5.000E-9		20.00	5.218E+17	0.2988		
Cycle: 42 10 nM					1.000E-8		20.00	5.218E+17	0.5063		
Cycle: 43 20 nM					2.000E-8		20.00	5.218E+17	0.7700		
Cycle: 44 40 nM					4.000E-8		20.00	5.218E+17	1.031		

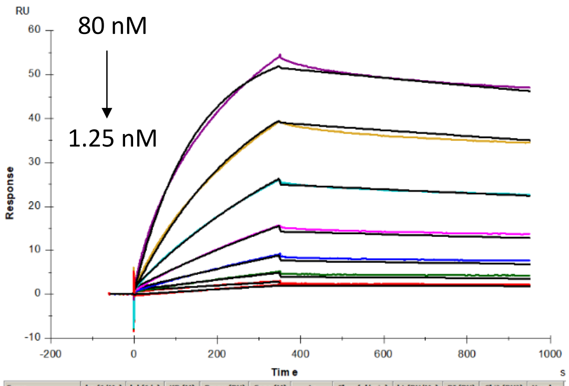

Curve	ka (1/Ms)	kd (1/s)	KD (M)	Rmax (RU)	Conc (M)	tc	Flow (ul/min)	kt (RU/Ms)	RI (RU)	Chi^2 (RU^2)	U-value
	8.281E+4	1.804E-4	2.179E-9	58.31		2.216E+17				0.324	3
Cycle: 34 1.25 nM					1.250E-9		20.00	6.014E+17	0.8208		
Cycle: 35 2.5 nM					2.500E-9		20.00	6.014E+17	0.9559		
Cycle: 36 5 nM					5.000E-9		20.00	6.014E+17	1.199		
Cycle: 37 10 nM					1.000E-8		20.00	6.014E+17	1.377		
Cycle: 38 20 nM					2.000E-8		20.00	6.014E+17	1.474		
Cycle: 39 40 nM					4.000E-8		20.00	6.014E+17	0.6320		
Cycle: 40 80 nM					8.000E-8		20.00	6.014E+17	0.4646		
Cycle: 41 1.25 nM					1.250E-9		20.00	6.014E+17	-0.06664		

PNA2-P3

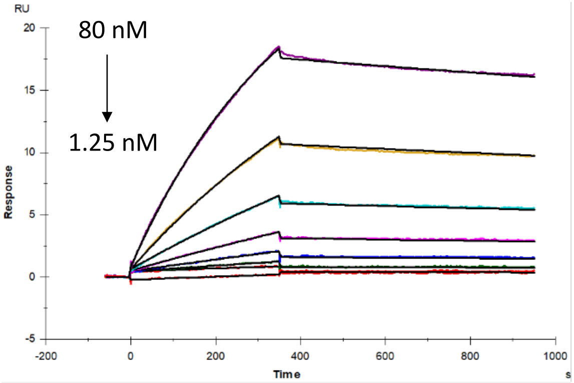

Curve	ka (1/Ms)	kd (1/s)	KD (M)	Rmax (RU)	Conc (M)	tc	Flow (ul/min)	kt (RU/Ms)	RI (RU)	Chi^2 (RU^2)	U-value
	3.143E+4	1.519E-4	4.833E-9	30.71		9.792E+14				0.0268	3
Cycle: 24 1.25 nM					1.250E-9		20.00	2.658E+15	0.4580		
Cycle: 25 2.5 nM					2.500E-9		20.00	2.658E+15	0.4422		
Cycle: 26 5 nM					5.000E-9		20.00	2.658E+15	0.4740		
Cycle: 27 10 nM					1.000E-8		20.00	2.658E+15	0.5241		
Cycle: 28 20 nM					2.000E-8		20.00	2.658E+15	0.6343		
Cycle: 29 40 nM					4.000E-8		20.00	2.658E+15	0.6417		
Cycle: 30 80 nM					8.000E-8		20.00	2.658E+15	0.8215		
Cycle: 31 1.25 nM					1.250E-9		20.00	2.658E+15	-0.2055		

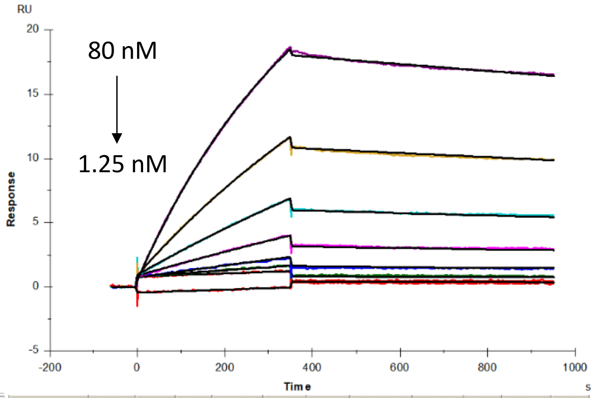

Curve	ka (1/Ms)	kd (1/s)	KD (M)	Rmax (RU)	Conc (M)	tc	Flow (ul/min)	kt (RU/Ms)	RI (RU)	Chi^2 (RU^2)	U-value
	2.941E+4	1.562E-4	5.311E-9	32.85		4.285E+15				0.0838	5
Cycle: 54 1.25 nM					1.250E-9		20.00	1.163E+16	0.8136		
Cycle: 55 2.5 nM					2.500E-9		20.00	1.163E+16	0.8241		
Cycle: 56 5 nM					5.000E-9		20.00	1.163E+16	0.7278		
Cycle: 57 10 nM					1.000E-8		20.00	1.163E+16	0.8819		
Cycle: 58 20 nM					2.000E-8		20.00	1.163E+16	0.9426		
Cycle: 59 40 nM					4.000E-8		20.00	1.163E+16	0.8953		
Cycle: 60 80 nM					8.000E-8		20.00	1.163E+16	0.5166		
Cycle: 61 1.25 nM					1.250E-9		20.00	1.163E+16	-0.4483		

DNA8

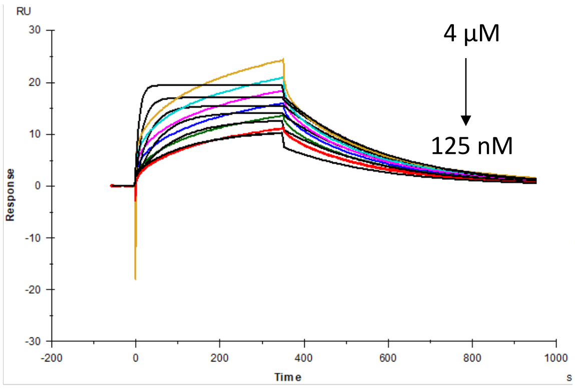

Curve	ka (1/Ms)	kd (1/s)	KD (M)	Rmax (RU)	Conc (M)	tc	Flow (ul/min)	kt (RU/Ms)	RI (RU)	Chi^2 (RU^2)	U-value
	2.778E+4	0.004234	1.524E-7	17.95		1.877E+8				1.54	4
Cycle: 4 125 nM					1.250E-7		20.00	5.095E+8	2.713		
Cycle: 5 250 nM					2.500E-7		20.00	5.095E+8	1.667		
Cycle: 6 500 nM					5.000E-7		20.00	5.095E+8	0.4664		
Cycle: 7 1000 nM					1.000E-6		20.00	5.095E+8	-0.06186		
Cycle: 8 2000 nM					2.000E-6		20.00	5.095E+8	0.4380		
Cycle: 9 4000 nM					4.000E-6		20.00	5.095E+8	2.183		
Cycle: 10 125 nM					1.250E-7		20.00	5.095E+8	2.786		

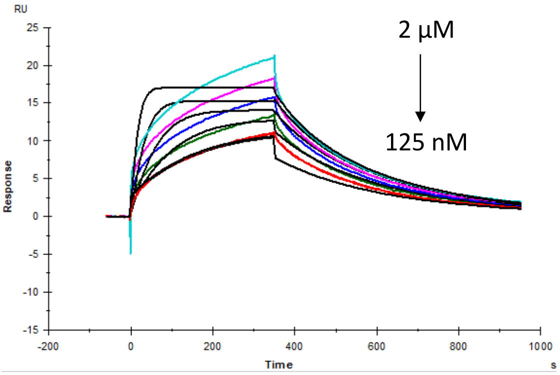

Curve	ka (1/Ms)	kd (1/s)	KD (M)	Rmax (RU)	Conc (M)	tc	Flow (ul/min)	kt (RU/Ms)	RI (RU)	Chi^2 (RU^2)	U-value
	4.106E+4	0.005445	1.326E-7	17.56		3.529E+5				0.944	4
Cycle: 4 125.5 nM					1.255E-7		20.00	9.580E+5	2.683		
Cycle: 5 250 nM					2.500E-7		20.00	9.580E+5	1.458		
Cycle: 6 500 nM					5.000E-7		20.00	9.580E+5	0.2073		
Cycle: 7 1000 nM					1.000E-6		20.00	9.580E+5	-0.1707		
Cycle: 8 2000 nM					2.000E-6		20.00	9.580E+5	0.6028		
Cycle: 9 125.5 nM					1.255E-7		20.00	9.580E+5	2.838		

PNA3-AAmm-Bt on chip A:A mismatch

PNA2-ach

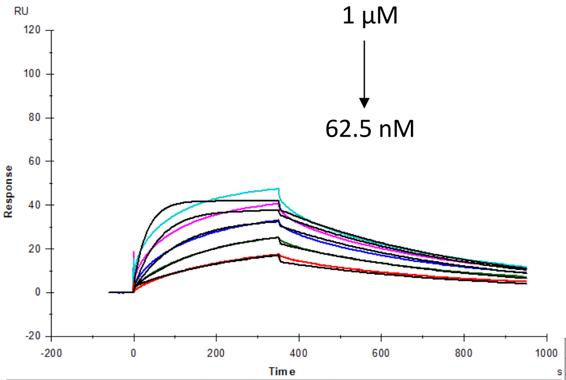

Curve	ka (1/Ms)	kd (1/s)	KD (M)	Rmax (RU)	Conc (M)	tc	Flow (ul/min)	kt (RU/Ms)	RI (RU)	Chi² (RU²)	U-value
	2.966E+4	0.002106	7.101E-8	40.91		1.013E+7				2.10	2
Cycle: 12 62.5 nM					6.250E-8		20.00	2.749E+7	2.947		
Cycle: 13 125 nM					1.250E-7		20.00	2.749E+7	2.787		
Cycle: 14 250 nM					2.500E-7		20.00	2.749E+7	1.822		
Cycle: 15 500 nM					5.000E-7		20.00	2.749E+7	1.875		
Cycle: 16 1000 nM					1.000E-6		20.00	2.749E+7	3.856		
Cycle: 17 62.5 nM					6.250E-8		20.00	2.749E+7	3.038		

DNA8

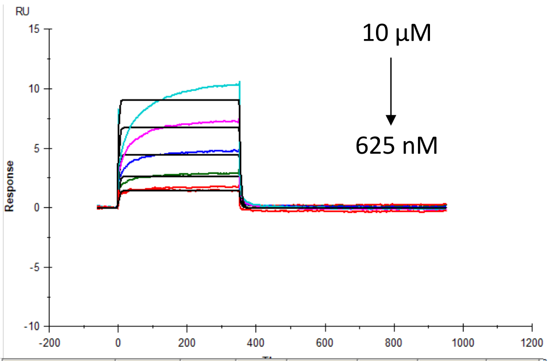

Curve	ka (1/Ms)	kd (1/s)	KD (M)	Rmax (RU)	Conc (M)	Chi² (RU²)	U-value
	4.941E+4	0.2582	5.225E-6	13.76		0.320	N/A
Cycle: 4 625 nM					6.250E-7		
Cycle: 5 1250 nM					1.250E-6		
Cycle: 6 2500 nM					2.500E-6		
Cycle: 7 5000 nM					5.000E-6		
Cycle: 8 10000 nM					1.000E-5		
Cycle: 9 625 nM					6.250E-7		

PNA2-H1

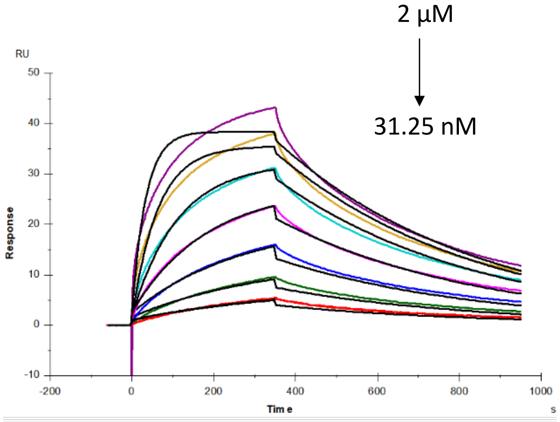

Curve	ka (1/Ms)	kd (1/s)	KD (M)	Rmax (RU)	Conc (M)	tc	Flow (ul/min)	kt (RU/Ms)	RI (RU)	Chi² (RU²)	U-value
	1.382E+4	0.002035	1.473E-7	39.49		1.887E+13				1.10	1
Cycle: 34 31.25 nM					3.125E-8		20.00	5.122E+13	1.012		
Cycle: 35 62.5 nM					6.250E-8		20.00	5.122E+13	1.682		
Cycle: 36 125 nM					1.250E-7		20.00	5.122E+13	2.445		
Cycle: 37 250 nM					2.500E-7		20.00	5.122E+13	2.559		
Cycle: 38 500 nM					5.000E-7		20.00	5.122E+13	1.762		
Cycle: 39 1000 nM					1.000E-6		20.00	5.122E+13	1.143		
Cycle: 40 2000 nM					2.000E-6		20.00	5.122E+13	1.638		
Cycle: 41 31.25 nM					3.125E-8		20.00	5.122E+13	1.108		

PNA-H3

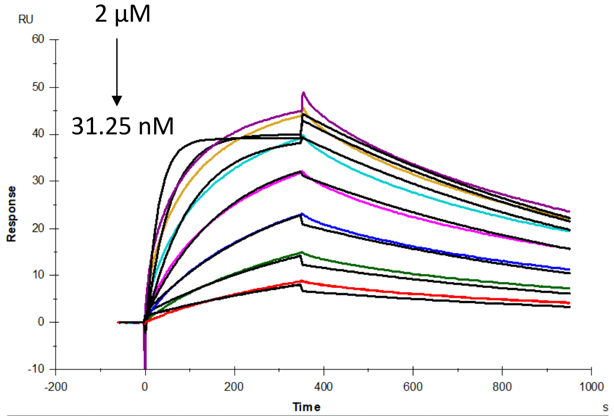

Curve	ka (1/Ms)	kd (1/s)	KD (M)	Rmax (RU)	Conc (M)	tc	Flow (ul/min)	kt (RU/Ms)	RI (RU)	Chi² (RU²)	U-value
	1.768E+4	0.001156	6.535E-8	45.90		3.412E+14				2.08	2
Cycle: 44 31.25 nM					3.125E-8		20.00	9.262E+14	1.393		
Cycle: 45 62.5 nM					6.250E-8		20.00	9.262E+14	1.938		
Cycle: 46 125 nM					1.250E-7		20.00	9.262E+14	2.007		
Cycle: 47 250 nM					2.500E-7		20.00	9.262E+14	0.9595		
Cycle: 48 500 nM					5.000E-7		20.00	9.262E+14	-1.192		
Cycle: 49 1000 nM					1.000E-6		20.00	9.262E+14	-3.058		
Cycle: 50 2000 nM					2.000E-6		20.00	9.262E+14	-5.325		
Cycle: 51 31.25 nM					3.125E-8		20.00	9.262E+14	1.427		

PNA2-A3

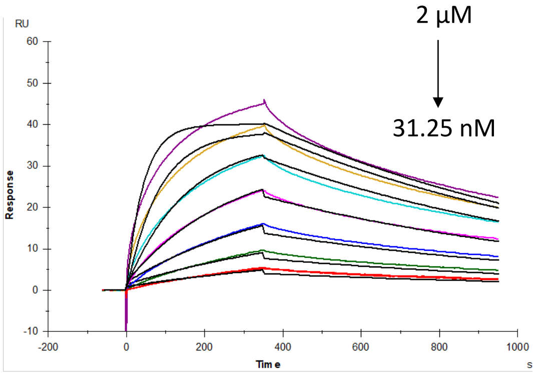

Curve	ka (1/Ms)	kd (1/s)	KD (M)	Rmax (RU)	Conc (M)	tc	Flow (ul/min)	kt (RU/Ms)	RI (RU)	Chi² (RU²)	U-value
	1.112E+4	0.001085	9.761E-8	42.42		1.978E+14				1.42	2
Cycle: 64 31.25 nM					3.125E-8		20.00	5.370E+14	0.9517		
Cycle: 65 62.5 nM					6.250E-8		20.00	5.370E+14	1.430		
Cycle: 66 125 nM					1.250E-7		20.00	5.370E+14	1.895		
Cycle: 67 250 nM					2.500E-7		20.00	5.370E+14	1.841		
Cycle: 68 500 nM					5.000E-7		20.00	5.370E+14	0.7315		
Cycle: 69 1000 nM					1.000E-6		20.00	5.370E+14	-0.4145		
Cycle: 70 2000 nM					2.000E-6		20.00	5.370E+14	-0.3439		
Cycle: 71 31.25 nM					3.125E-8		20.00	5.370E+14	0.8654		

PNA-P3

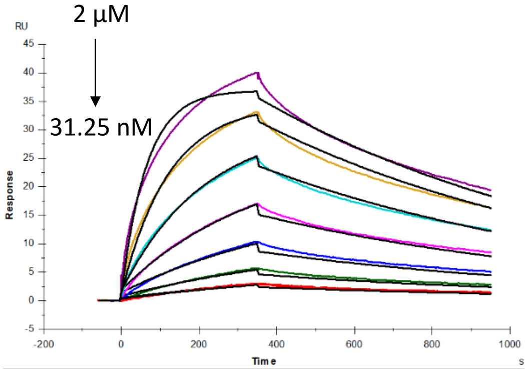

Curve	ka (1/Ms)	kd (1/s)	KD (M)	Rmax (RU)	Conc (M)	tc	Flow (ul/min)	kt (RU/Ms)	RI (RU)	Chi² (RU²)	U-value
	7115	0.001104	1.552E-7	38.61		7.363E+13				0.489	1
Cycle: 54 31.25 nM					3.125E-8		20.00	1.999E+14	0.4922		
Cycle: 55 62.5 nM					6.250E-8		20.00	1.999E+14	0.8493		
Cycle: 56 125 nM					1.250E-7		20.00	1.999E+14	1.441		
Cycle: 57 250 nM					2.500E-7		20.00	1.999E+14	1.858		
Cycle: 58 500 nM					5.000E-7		20.00	1.999E+14	1.716		
Cycle: 59 1000 nM					1.000E-6		20.00	1.999E+14	1.187		
Cycle: 60 2000 nM					2.000E-6		20.00	1.999E+14	1.115		
Cycle: 61 31.25 nM					3.125E-8		20.00	1.999E+14	0.3818		

PNA2-N3

2 µM

31.25 nM

PNA-G3

2 µM

31.25 nM

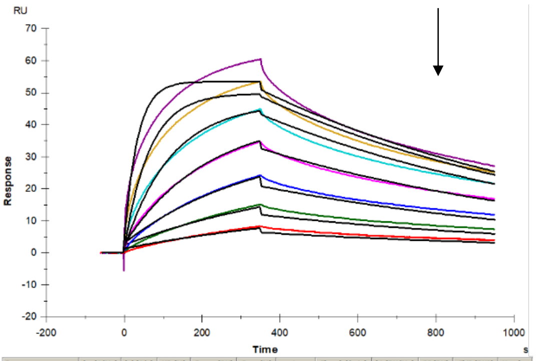

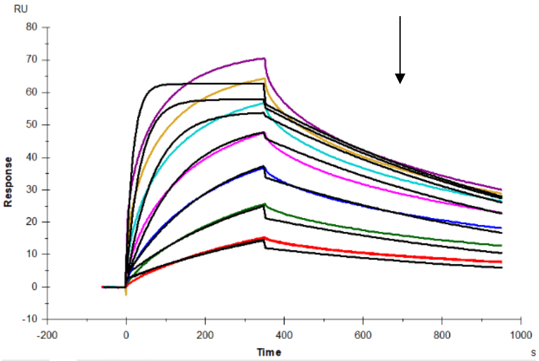

Curve	ka (1/Ms)	kd (1/s)	KD (M)	Rmax (RU)	Conc (M)	tc	Flow (ul/min)	kt (RU/Ms)	RI (RU)	Chi² (RU²)	U-value
	1.444E+4	0.001159	8.028E-8	52.97		3.309E+14				2.42	2
Cycle: 14 31.25 nM					3.125E-8		20.00	8.983E+14	1.403		
Cycle: 15 62.5 nM					6.250E-8		20.00	8.983E+14	2.444		
Cycle: 16 125 nM					1.250E-7		20.00	8.983E+14	3.017		
Cycle: 17 250 nM					2.500E-7		20.00	8.983E+14	2.546		
Cycle: 18 500 nM					5.000E-7		20.00	8.983E+14	1.174		
Cycle: 19 1000 nM					1.000E-6		20.00	8.983E+14	0.7718		
Cycle: 20 2000 nM					2.000E-6		20.00	8.983E+14	2.594		
Cycle: 21 31.25 nM					3.125E-8		20.00	8.983E+14	1.376		

Curve	ka (1/Ms)	kd (1/s)	KD (M)	Rmax (RU)	Conc (M)	tc	Flow (ul/min)	kt (RU/Ms)	RI (RU)	Chi² (RU²)	U-value
	2.649E+4	0.001174	4.431E-8	57.82		6.650E+18				5.04	2
Cycle: 34 31.25 nM					3.125E-8		20.00	1.805E+19	2.453		
Cycle: 35 62.5 nM					6.250E-8		20.00	1.805E+19	3.806		
Cycle: 36 125 nM					1.250E-7		20.00	1.805E+19	3.551		
Cycle: 37 250 nM					2.500E-7		20.00	1.805E+19	1.919		
Cycle: 38 500 nM					5.000E-7		20.00	1.805E+19	0.9804		
Cycle: 39 1000 nM					1.000E-6		20.00	1.805E+19	2.471		
Cycle: 40 2000 nM					2.000E-6		20.00	1.805E+19	6.112		
Cycle: 41 31.25 nM					3.125E-8		20.00	1.805E+19	2.615		

DNA7-Bt on chip A:A mismatch (No binding observed at the concentrations tested)

PNA2-H3

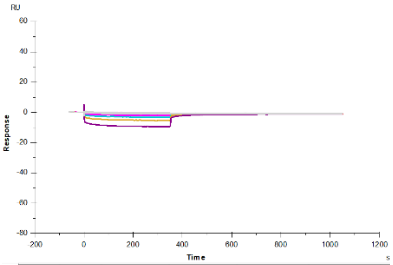

Include	Cycle#	Conc (nM)	Flow (µl/min)	Contact Time (s)	Diss. Time (s)
✓	32	0	20	351.0	699.9
✓	33	31.25	20	351.1	699.9
✓	34	62.5	20	351.1	699.9
✓	35	125	20	351.0	699.9
✓	36	250	20	351.0	699.9
✓	37	500	20	351.0	700.0
✓	38	1000	20	351.0	700.0
✓	39	2000	20	351.0	700.0
✓	40	31.25	20	351.1	699.9
✓	41	0	20	351.0	700.0

PNA2-A3

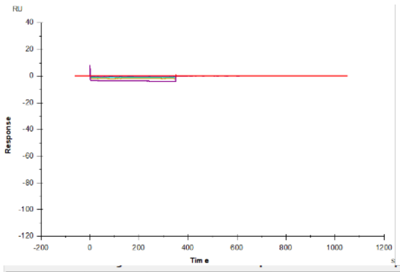

Edit	Cycle#	Curve	Conc (nM)	Flow (µl/min)	Contact Time (s)	Diss. Time (s)
✓	53	Fc=4-3	31.25	20	351.0	700.0
✓	54	Fc=4-3	62.5	20	351.1	699.9
✓	55	Fc=4-3	125	20	351.0	700.0
✓	56	Fc=4-3	250	20	351.1	699.9
✓	57	Fc=4-3	500	20	351.0	700.0
✓	58	Fc=4-3	1000	20	351.1	699.9
✓	59	Fc=4-3	2000	20	351.0	699.9
✓	60	Fc=4-3	31.25	20	351.0	699.9

PNA2-P3

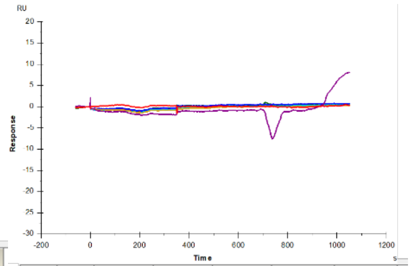

Edit	Cycle#	Curve	Conc (nM)	Flow (µl/min)	Contact Time (s)	Diss. Time (s)
✓	53	Fc=4-3	31.25	20	351.0	700.0
✓	54	Fc=4-3	62.5	20	351.0	700.0
✓	55	Fc=4-3	125	20	351.1	699.9
✓	56	Fc=4-3	250	20	351.0	699.9
✓	57	Fc=4-3	500	20	351.1	699.9
✓	58	Fc=4-3	1000	20	351.0	699.9
✓	59	Fc=4-3	2000	20	351.1	699.9
✓	60	Fc=4-3	31.25	20	351.0	699.9

PNA2-N3

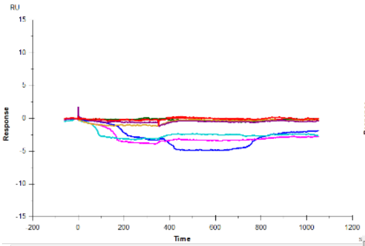

Edit	Cycle#	Curve	Conc (nM)	Flow (µl/min)	Contact Time (s)	Diss. Time (s)
✓	13	Fc=4-3	31.25	20	351.0	699.9
✓	14	Fc=4-3	62.5	20	351.1	699.9
✓	15	Fc=4-3	125	20	351.1	699.9
✓	16	Fc=4-3	250	20	351.0	700.0
✓	17	Fc=4-3	500	20	351.0	699.9
✓	18	Fc=4-3	1000	20	351.0	700.0
✓	19	Fc=4-3	2000	20	351.0	699.9
✓	20	Fc=4-3	31.25	20	351.0	699.9

PNA2-G3

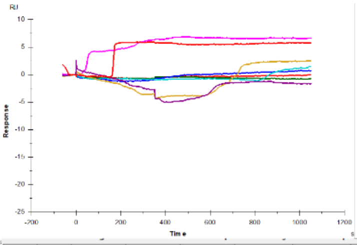

Edit	Cycle#	Curve	Conc (nM)	Flow (µl/min)	Contact Time (s)	Diss. Time (s)
✓	33	Fc=4-3	31.25	20	351.0	699.9
✓	34	Fc=4-3	62.5	20	351.0	699.9
✓	35	Fc=4-3	125	20	351.0	699.9
✓	36	Fc=4-3	250	20	351.0	699.9
✓	37	Fc=4-3	500	20	351.0	699.9
✓	38	Fc=4-3	1000	20	351.0	699.9
✓	39	Fc=4-3	2000	20	351.0	700.0
✓	40	Fc=4-3	31.25	20	351.0	700.0

PNA2-H1

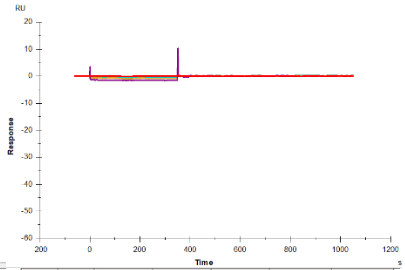

Edit	Cycle#	Curve	Conc (nM)	Flow (µl/min)	Contact Time (s)	Diss. Time (s)
✓	23	Fc=4-3	31.25	20	351.1	699.9
✓	24	Fc=4-3	62.5	20	351.1	699.9
✓	25	Fc=4-3	125	20	351.0	699.9
✓	26	Fc=4-3	250	20	351.0	700.0
✓	27	Fc=4-3	500	20	351.0	700.0
✓	28	Fc=4-3	1000	20	351.0	700.0
✓	29	Fc=4-3	2000	20	351.0	700.0
✓	30	Fc=4-3	31.25	20	351.0	699.9

Copies of the ^1H and ^{13}C NMR spectra for compounds Az-A/H/N/G/P

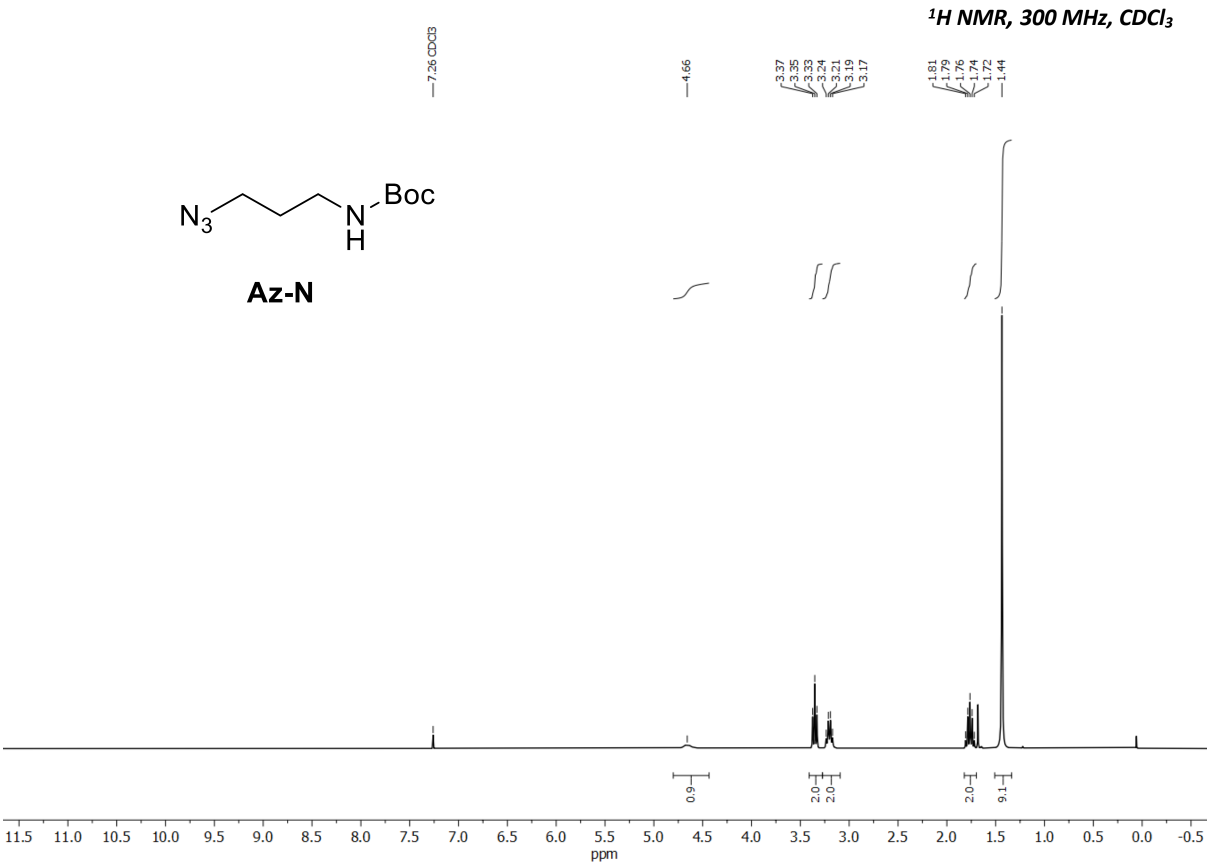

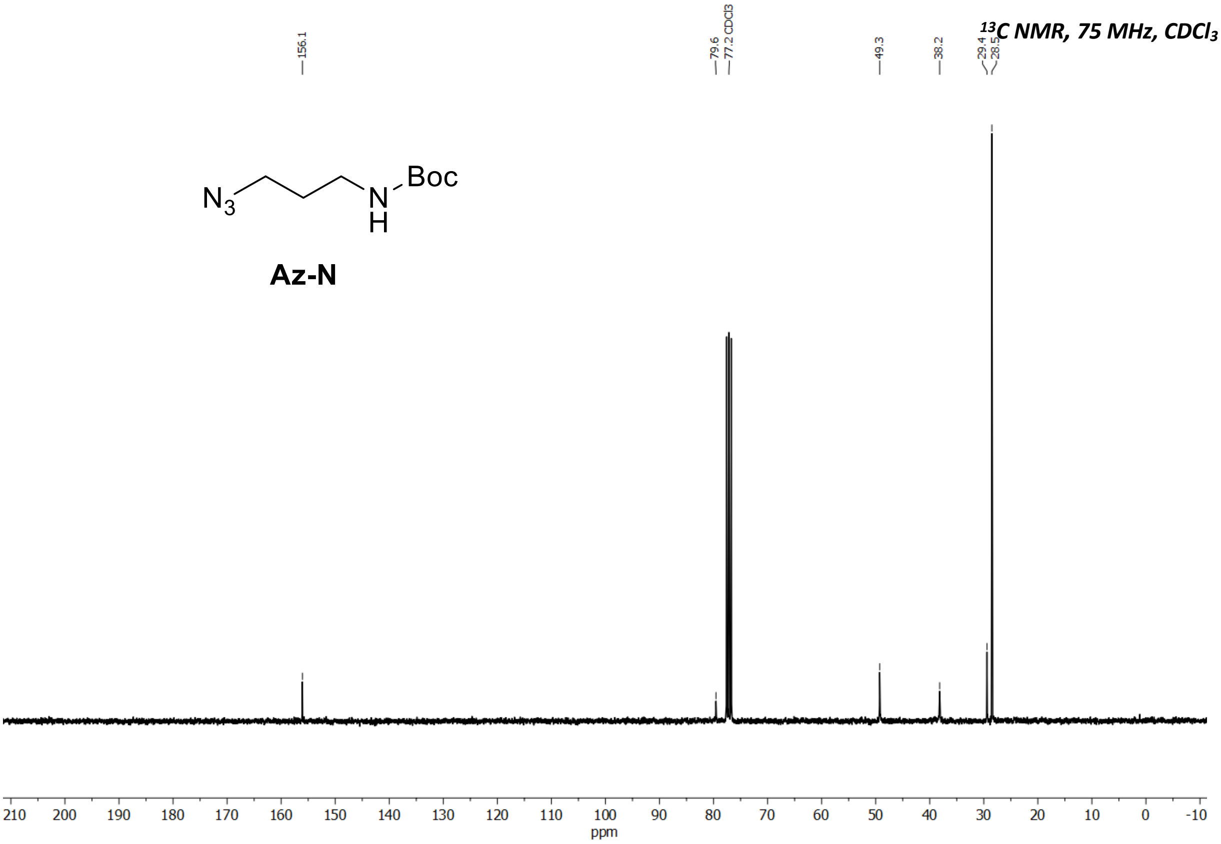

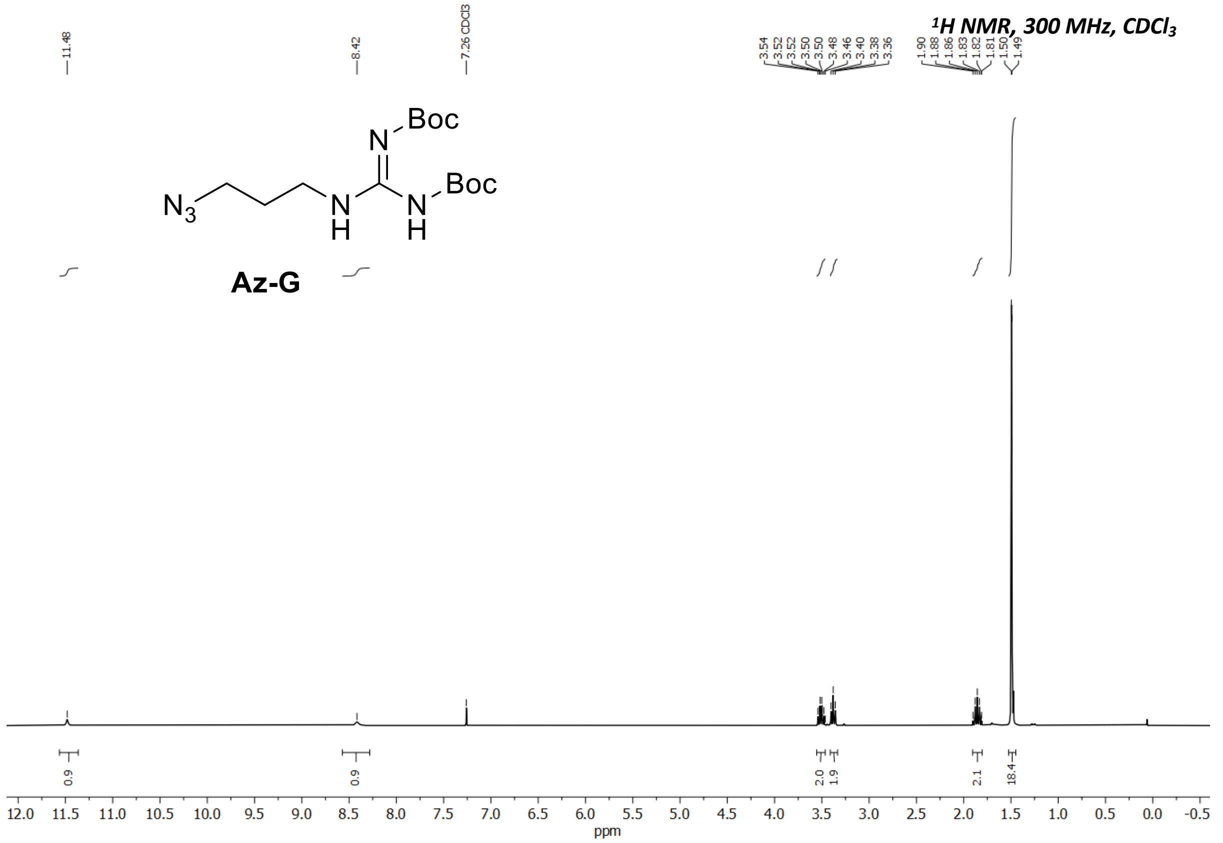
1H NMR, 300 MHz, CDCl3
11.48
8.42
7.26 CDCl3
3.54 3.52 3.52 3.50 3.50 3.48 3.46 3.40 3.38 3.36
1.90 1.88 1.86 1.83 1.82 1.81 1.50 1.49
Boc
N
Boc
N3
N H
N H
Az-G
0.9
0.9
2.0
1.9
2.1
18.4
ppm

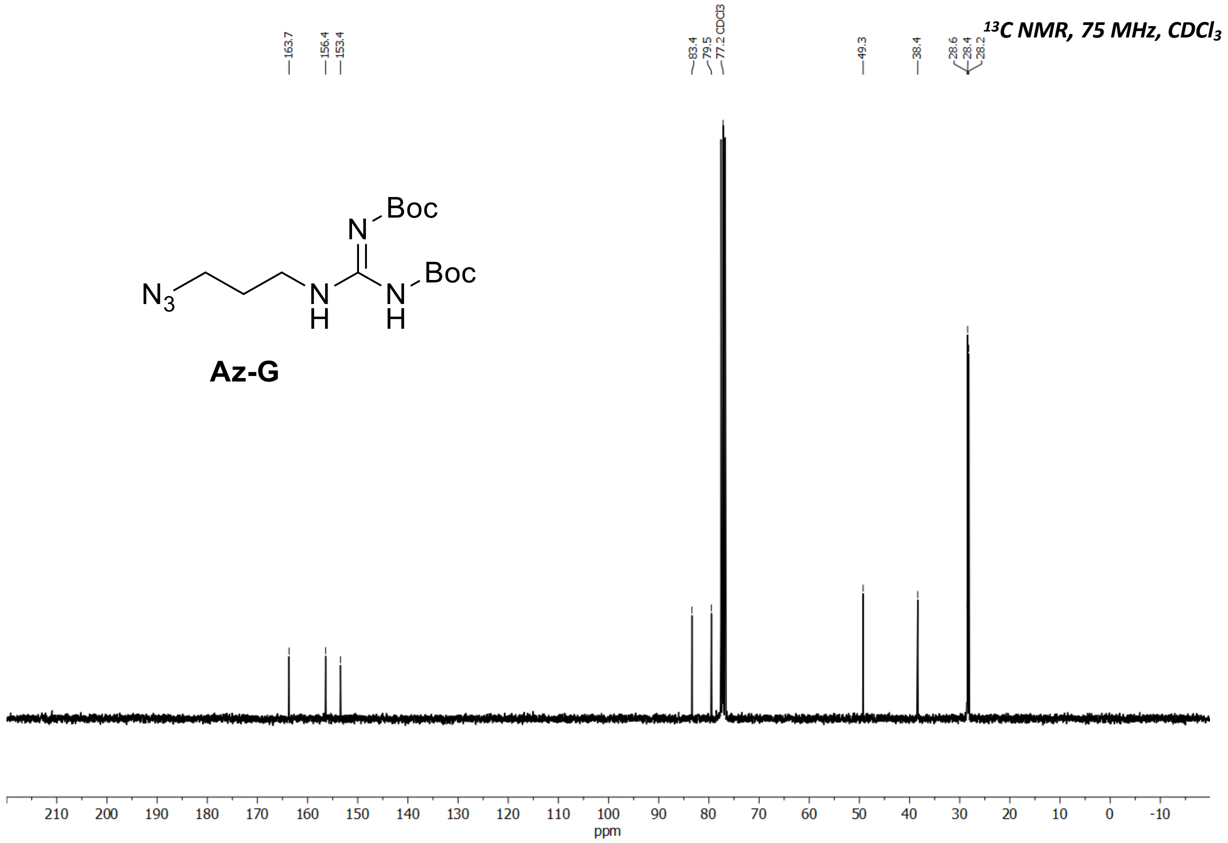
13C NMR, 75 MHz, CDCl3
163.7
156.4
153.4
83.4
79.5
77.2 CDCl3
49.3
38.4
28.6 28.4 28.2
Boc
N
Boc
N3
N H
N H
Az-G
ppm

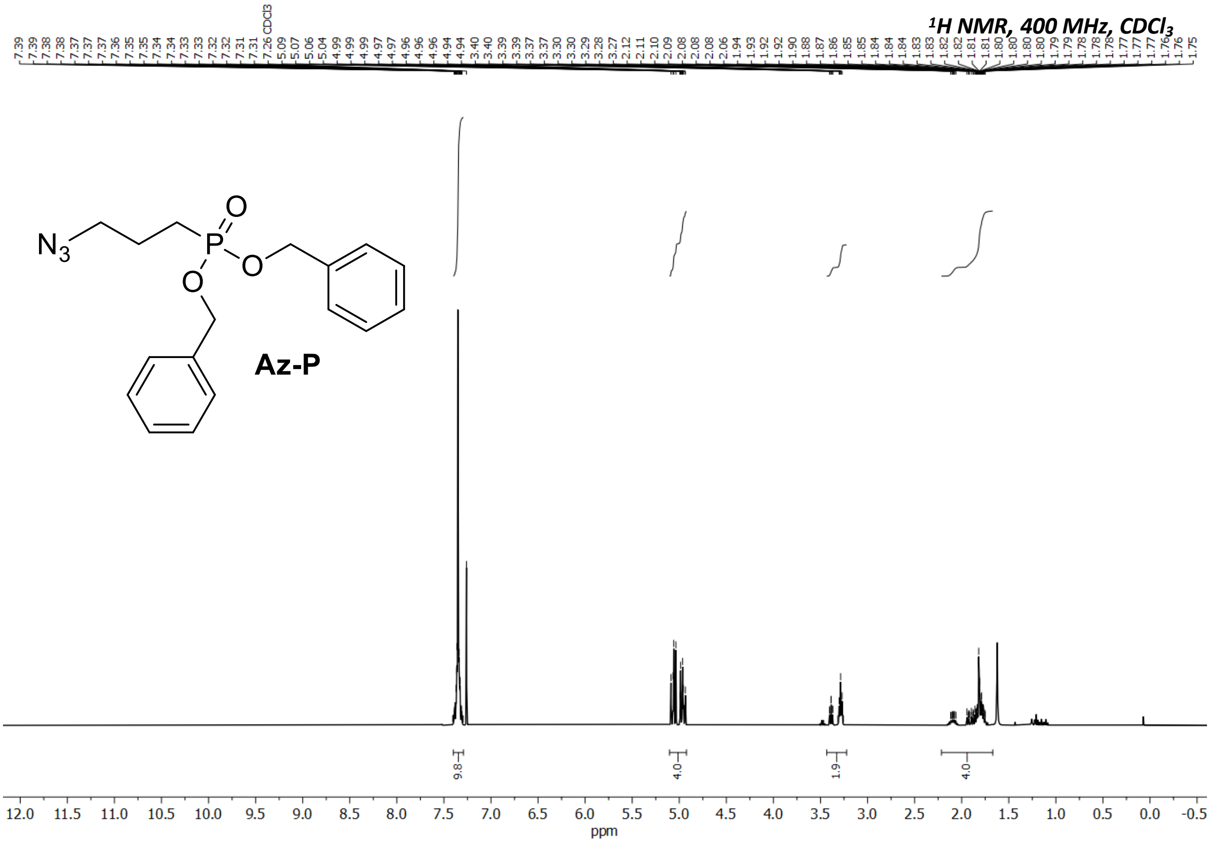
1H NMR, 400 MHz, CDCl3
Az-P
ppm

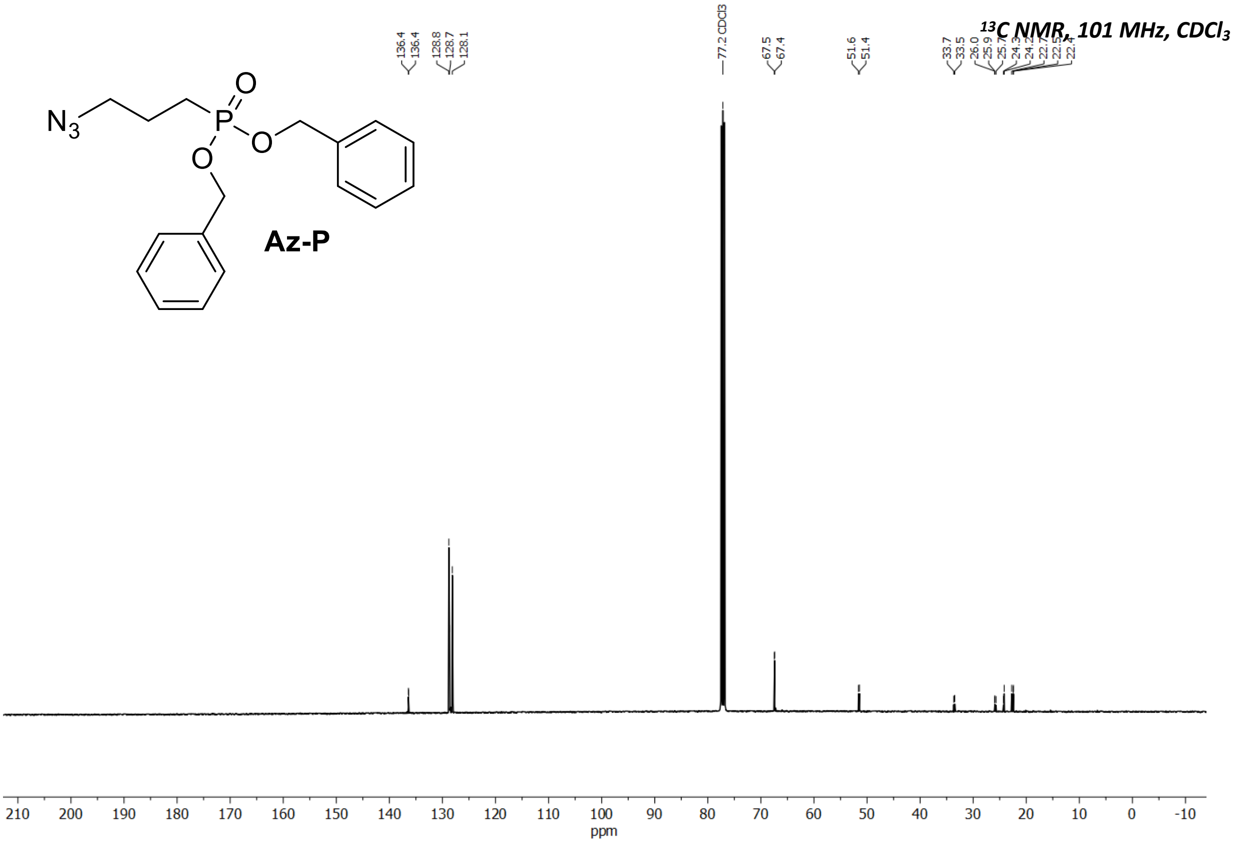
13C NMR, 101 MHz, CDCl3
Az-P
ppm

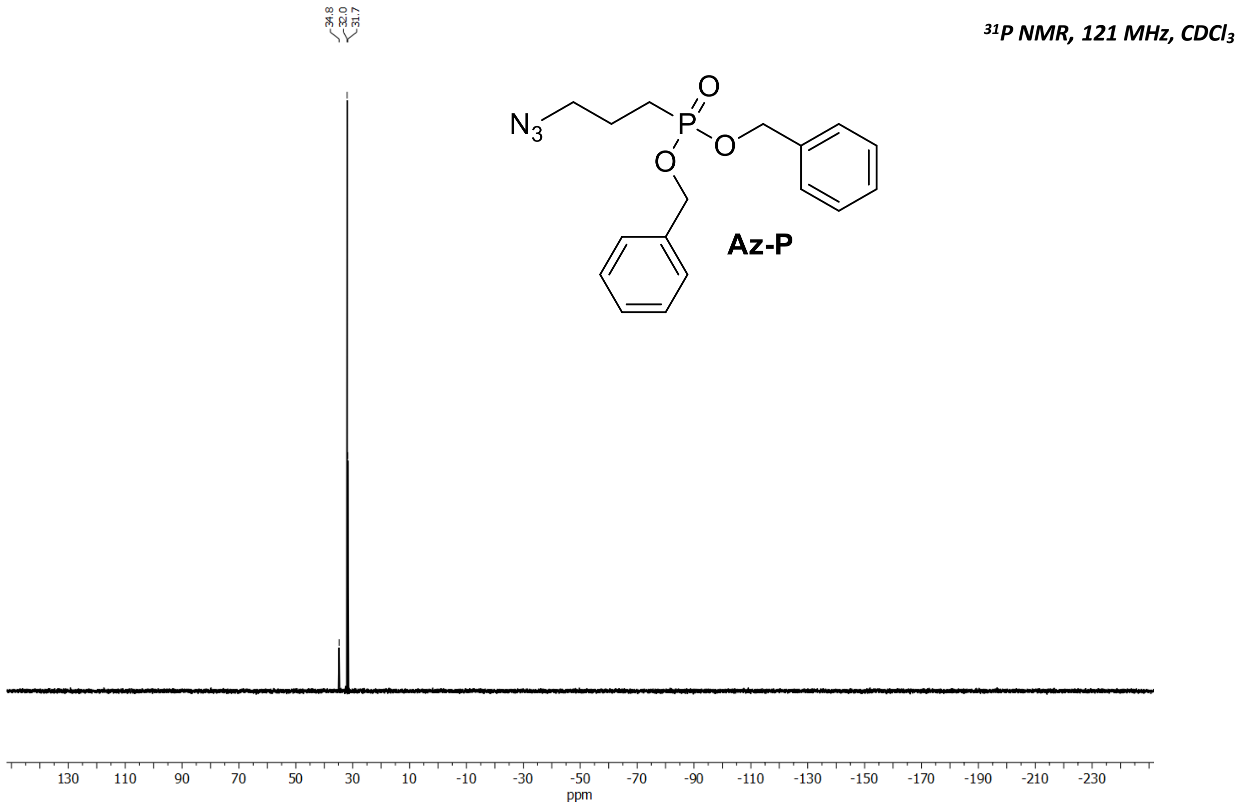
^{31}P NMR, 121 MHz, CDCl$_3$
34.8
32.0
31.7
N3
P
O
O
O
Az-P
130 110 90 70 50 30 10 -10 -30 -50 -70 -90 -110 -130 -150 -170 -190 -210 -230
ppm

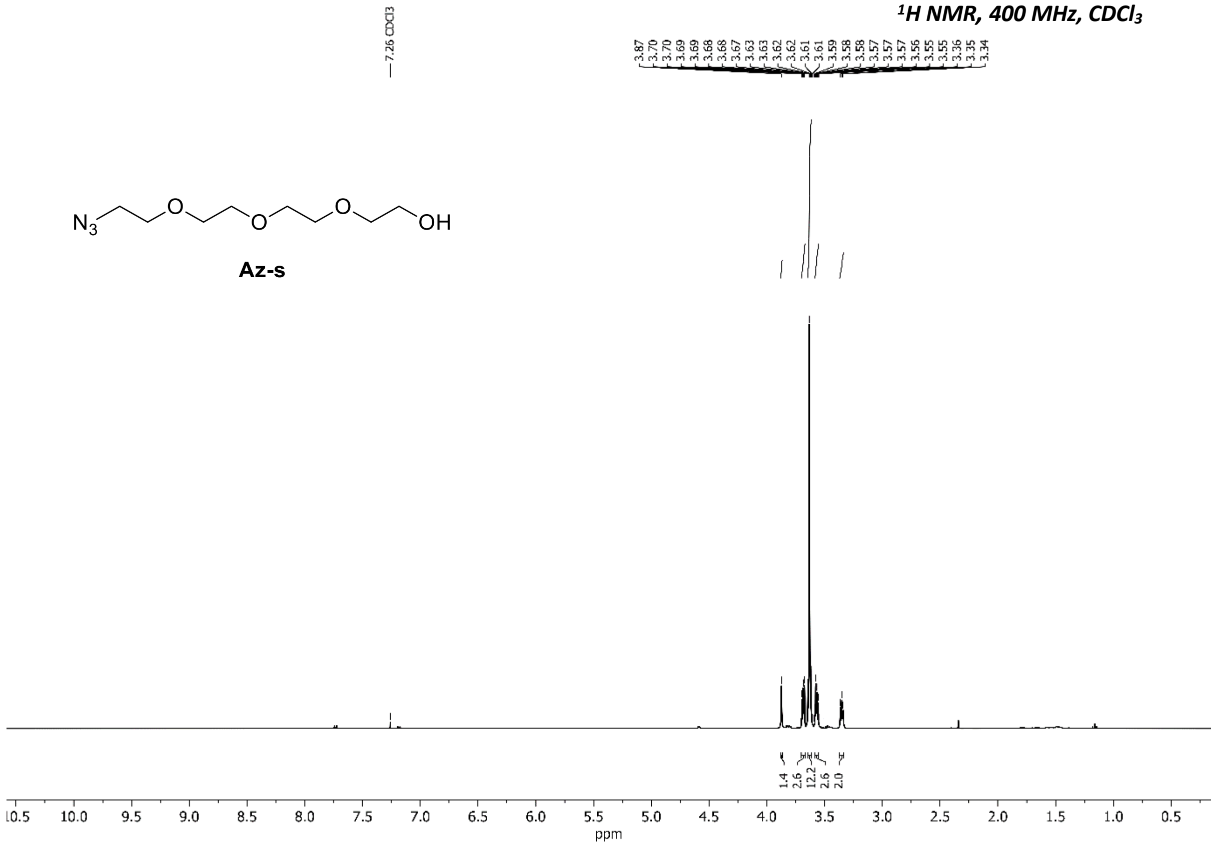
^{1}H NMR, 400 MHz, CDCl$_3$
7.26 CDCl3
3.87 3.70 3.70 3.69 3.69 3.68 3.68 3.67 3.63 3.63 3.62 3.62 3.61 3.61 3.59 3.58 3.58 3.57 3.57 3.56 3.55 3.55 3.36 3.35 3.34
N3
O
O
O
OH
Az-s
1.4 2.6 12.2 2.6 2.0
10.5 10.0 9.5 9.0 8.5 8.0 7.5 7.0 6.5 6.0 5.5 5.0 4.5 4.0 3.5 3.0 2.5 2.0 1.5 1.0 0.5
ppm

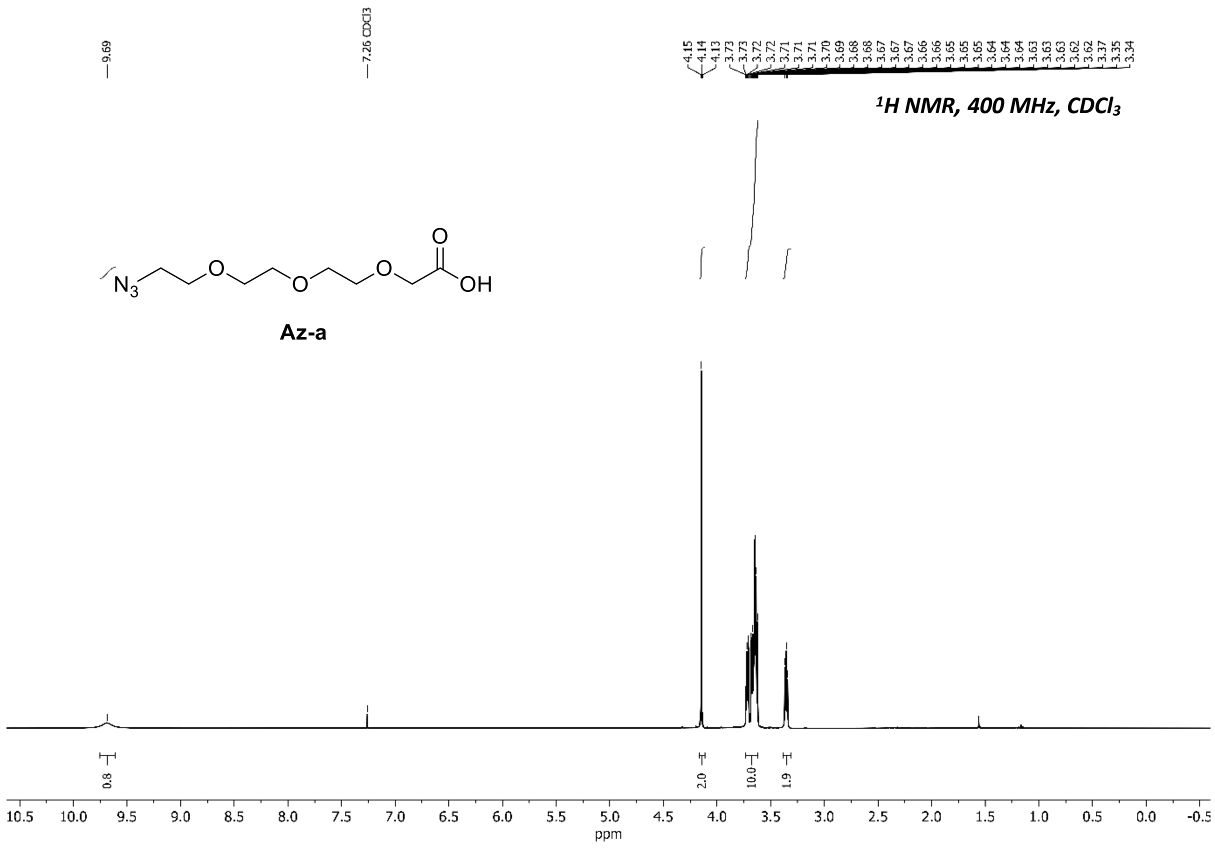

References

(1) Chouikhi, D.; Ciobanu, M.; Zambaldo, C.; Duplan, V.; Barluenga, S.; Winssinger, N. Expanding the scope of PNA-encoded synthesis (PES): Mtt-protected PNA fully orthogonal to fmoc chemistry and a broad array of robust diversity-generating reactions. *Chemistry* **2012**, *18* (40), 12698-12704.

(2) Lopez-Tena, M.; Farrera-Soler, L.; Barluenga, S.; Winssinger, N. Pseudo-Complementary G:C Base Pair for Mixed Sequence dsDNA Invasion and Its Applications in Diagnostics (SARS-CoV-2 Detection). *JACS Au* **2023**, *3* (2), 449-458.

(3) Khiar, N.; Leal, M. P.; Baati, R.; Ruhlmann, C.; Mioskowski, C.; Schultz, P.; Fernandez, I. Tailoring carbon nanotube surfaces with glyconanorings: new bionanomaterials with specific lectin affinity. *Chem Commun (Camb)* **2009**, (27), 4121-4123.

(4) Xiao, S.; Fu, N.; Peckham, K.; Smith, B. D. Efficient synthesis of fluorescent squaraine rotaxane dendrimers. *Org Lett* **2010**, *12* (1), 140-143.
